# New Cannabinoids and Chlorin-Type Metabolites from the Flowers of *Cannabis sativa* L.: A Study on Their Neuroblastoma Activity

**DOI:** 10.3390/ph18040521

**Published:** 2025-04-03

**Authors:** Tuan-Quoc Nguyen, Hyo-Shin Park, Sun-Hyeong Choi, Da-Yun Hong, Jae-Yong Cheon, Young-Mi Lee, Chul-Min Kim, Jong-Ki Hong, Seo-Jeong Oh, Man-Soo Cho, Jang-Hoon Kim, Eun-Sol Lee, Jungwon Seo, Hyun-Ju Jung

**Affiliations:** 1 Department of Oriental Pharmacy, College of Pharmacy and Wonkwang-Oriental Medicines Reseach Institute, Wonkwang University, Iksan 570-749, Republic of Korea; quoctuan301281@gmail.com (T.-Q.N.); phs4846@naver.com (H.-S.P.); subear780@gmail.com (S.-H.C.); hong_13@naver.com (D.-Y.H.); cjy21750@naver.com (J.-Y.C.); ymlee@wku.ac.kr (Y.-M.L.); 2 Korean Ministry of Food and Drug Safety, Osongsaengmyeong 2-ro 187, Cheongju 28159, Republic of Korea; 3 Department of Horticulture Industry, Wonkwang University, Iksan 54538, Republic of Korea; chumin21@wku.ac.kr; 4 College of Pharmacy, Kyung Hee University, Seoul 02447, Republic of Korea; jhong@khu.ac.kr (J.-K.H.); dhtjwjd333@naver.com (S.-J.O.); 5 Department of Smart Experience Design, Kookmin University, Seoul 02707, Republic of Korea; chomansoo@gmail.com; 6 Department of Herbal Crop Research, National Institute of Horticultural and Herbal Science, RDA, Eumseong 27709, Republic of Korea; jhkim53@korea.kr; 7 Institute of Pharmaceutical Research and Development, College of Pharmacy, Wonkwang University, Iksan 54538, Republic of Korea; chori0509@naver.com (E.-S.L.); jwseo@wku.ac.kr (J.S.)

**Keywords:** *Cannabis sativa* L., cannabinoid, chlorin-type compound, SK-N-SH cells

## Abstract

**Background/Objectives**: *Cannabis sativa* has been utilized for medical purposes for thousands of years. It continues to be recognized as a plant with an extensive variety of medicinal and nutraceutical uses today. In this study, a chemical investigation of the flowers of *C. sativa* isolated by using a variety of chromatographic techniques led to the isolation of eleven compounds. These purified compounds were evaluated for antitumor activity against SK-N-SH neuroblastoma cells. **Methods**: The compounds were isolated by using chromatographic techniques. Their structures were identified by the examination of spectroscopic methods, including 1D (^1^H, ^13^C, and DEPT) and 2D (COSY, HSQC, HMBC, and NOESY) nuclear magnetic resonance (NMR) spectra and mass spectrum, together with the comparison to those reported previously in the literature. The evaluation of toxicity on SK-N-SH cells was performed by the MTT method. **Results**: Eleven compounds were isolated from the flowers of *C. sativa*, including two new compounds, namely cannabielsoxa (**1**), 13^2^-hydroxypheophorbide c ethyl ester (**2**), and six known cannabinoids (**6**–**11**), together with the first isolation of chlorin-type compounds: pyropheophorbide A (**3**), 13^2^-hydroxypheophorbide b ethyl ester (**4**), and ligulariaphytin A (**5**) from this plant. The results also demonstrated that cannabinoid compounds had stronger inhibitory effects on neuroblastoma cells than chlorin-type compounds. **Conclusions**: The evaluation of the biological activities of compounds showed that compounds **4**–**10** could be considered as the potential compounds for antitumor effects against neuroblastomas. This is also highlighted by using docking analysis. Additionally, the results of this study also suggest that these compounds have the potential to be developed into antineuroblastoma products.

## 1. Introduction

As a member of the Cannabaceae family, *Cannabis sativa* L. is among the world’s oldest plants to be cultivated for food, fiber, and medicine [1]. This plant has a long history of being used as a medicine to treat a variety of ailments, including asthma, epilepsy, fatigue, glaucoma, insomnia, nausea, pain, and rheumatism [1]. The studies on the chemical composition of *C. sativa* displayed that it contains more than 560 potentially bioactive compounds [2], including approximately 125 compounds belonging to the cannabinoids, and others as flavonoids (34 compounds), terpenes (120 compounds), phenolics (42 compounds), and alkaloids (2 compounds) [1]. Among them, cannabinoids were found as a specific chemical class and demonstrated that producing in the glandular trichomes of the plant [1,3]. Many studies reported the interesting biological effects of cannabinoids and treatments such as reducing nausea and vomiting by cannabidiol, cannabidiolic acid, and cannabidiolic acid methyl ester [4]. The compounds belonging to the cannabinoid group include cannabidiol (CBD), cannabichromene (CBC), cannabigerol (CBG), cannabinol (CBN), tetrahydrocannabinol (THC), tetrahydrocannabivarin (THCV), tetrahydrocannabinolic acid (THCA), cannabidavarin (CBDV), and cannabidiolic acid (CBDA) that were also demonstrated as antioxidant, anti-inflammatory, anticonvulsive, antipsychotic, antifungal, anticancer, antidepressant, antiarrhythmic effects, analgesic, antibiotic, anticonvulsant, immunomodulatory, neuroprotective, antineoplastic activity, and antiemetic properties [5]. *C. sativa* is also used as a drug type in the medicine depending on the content of Δ^9^-tetrahydrocannabinol (THC) [6,7]. *C. sativa* plant extracts with rich cannabinoid content exhibited antitumor effects in human neuroblastoma cells [8]. Cannabinoids were also reported to be involved in various types of cancers, including glioma, breast cancer, colorectal cancer, leukemia, prostate cancer, lung cancer, blood cancer, etc. [9]. They also reduced the viability of bladder cancer cell lines [10]. Many studies also published the effects of cannabinoids in animal models and/or patients related to anticancer [11,12,13]. For example, CBDA prevented the migration of breast cancer cells [14]; cannabinoids have especially potential benefits in the management of symptoms for patients living with and beyond cancer [15] and demonstrated anticancer effects through various receptors and ligands as well as modulation of signaling pathways of cancer pathology [9,16]. Clinical studies have shown that cannabinoids are effective in tumor regression and improving the health of patients [9].

Chlorin and its derivatives, including chlorophyll, pheophytin, chlorophyllin, and pheophobide, were found in most higher plants. They are interesting compounds for the development of new drugs. Chlorin derivatives exhibited toxicity against cancer cells, such as purpurin-7-dimethyl ester and pheophorbide A, which have been shown to exhibit strong cytotoxicity against seven solid tumor cell lines: human lung carcinoma (A549), ileocecal carcinoma (HCT-8), kidney carcinoma (CAKI-1), breast adenocarcinoma (MCF-7), malignant melanoma (SK-MEL-2), ovarian carcinoma (1A9), and epidermoid carcinoma of the nasopharynx (KB) [17]. In another study, it was also confirmed that chlorin derivatives have displayed strong inhibition of human A375 skin malignant melanoma cells, but these were less active against OE19 esophageal adenocarcinoma and HT1376 urinary bladder carcinoma cells [18]. Chlorin-type compounds are structurally related to porphyrins, which are known for their ability to generate reactive oxygen species (ROS) and have strong light-absorbing properties for photodynamic therapy (PDT) in cancer treatment [19,20,21]. Similarly, another study also disclosed chlorin-type compounds can enhance ROS production, which leads to oxidative damage and apoptosis in cancer cells [22]. In a continuation of our investigation on the chemical constituents and biological effects of *C. sativa*, a fascinating discovery is that in addition to the classes of chemicals above-mentioned, *C. sativa* also finds a group of chlorin-type compounds that has not previously been reported to be in this plant, as well as no study has evaluated the effects of cannabinoids or chlorin-type compounds on the SK-N-SH neuroblastoma cell lines. Here, we report the isolation and identification of new cannabinoid and chlorin-type compounds for the first time from the EtOAc extract of the flowers of *C. sativa* and their evaluated biological activity on SK-N-SH neuroblastoma cells. The discovery of chlorin compounds expands the therapeutic potential of *C. sativa*, highlighting its diverse bioactive profile.

## 2. Results and Discussion

### 2.1. Identification of the Isolated Compounds

**Figure 1:**
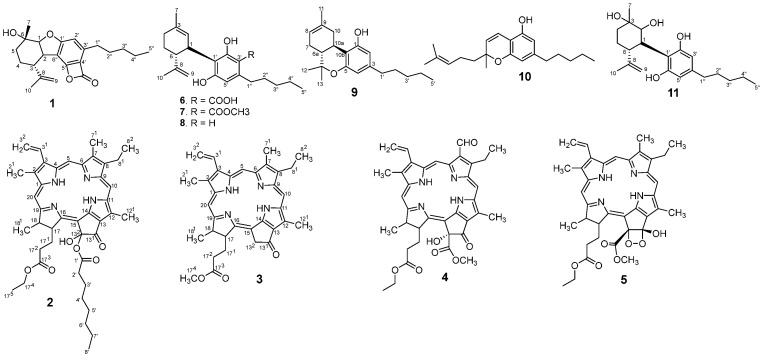
The structure of isolated compounds.

Two new compounds (**1** and **2**) and three chlorin-type compounds (**3**–**5**) for the first publication, together with six known cannabinoids, were isolated from the ethanol extract of the flowers of *C. sativa* (Figure 1). These compounds were identified by using NMR and HRESIMS data, and their identities were further confirmed through comparison with the literature.

**Figure 2:**
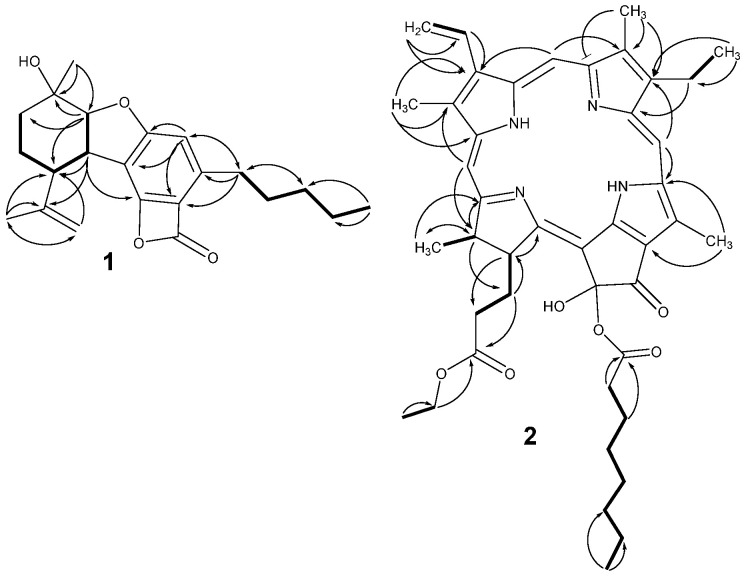
Key COSY (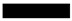) and HMBC (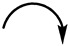) correlations of **1** and **2**.

**Figure 3:**
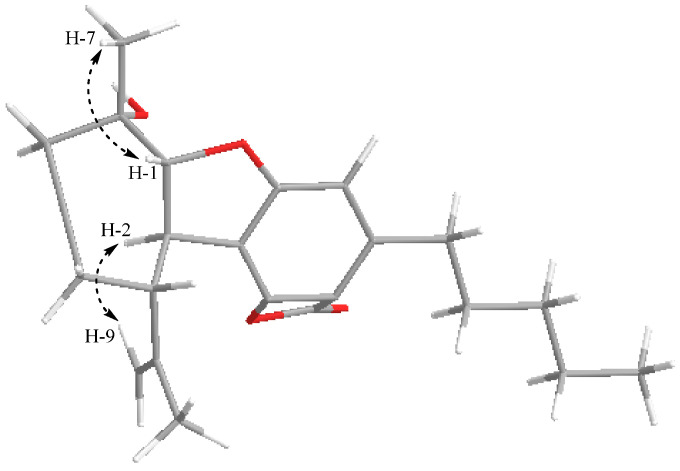
Key NOESY correlations of **1**.

Compound **1** was obtained as a colorless powder with the molecular formula C_22_H_28_O_4_ established by the peak at *m/z* 379.1896 [M + Na]^+^ [calcd. for [M + Na]^+^, *m/z* 379.1885] based on the HRESIMS spectrum. ^1^H and ^13^C NMR spectra of **1** indicated that it belongs to the cannabielsoin type with characteristic resonances at δ_H_: 1.52 (3H, *s*, H-7), 1.83 (3H, *s*, H-10), 0.88 (3H, *t*, *J* = 6.8 Hz, H-5″), 5.06 (2H, *d*, *J* = 3.6 Hz, H-9), 4.34 (1H, *d*, *J* = 5.6 Hz, H-1), and 6.41 (1H, *s*, H-2′). These signals displayed the connectivity of the protons and C-atoms in the HMQC spectrum at δ_C_ 28.2 (C-7), 22.7 (C-10), 14.2 (C-5″), 111.6 (C-9), 91.3 (C-1), and 114.1 (C-2′), respectively, and presented in Table 1. In the ^1^H-^1^H COSY spectrum exhibited correlations of protons such as H-1 (δ_H_ 4.34)/H-2 (δ_H_ 3.44); H-2 (δ_H_ 3.44)/H-3 (δ_H_ 1.89); H-3 (δ_H_ 1.89)/H-4 (δ_H_ 1.56); and H-4 (*δ_H_* 1.56)/H-5 (δ_H_ 1.74); and H-1″ (δ_H_ 2.92)/H-2″ (δ_H_ 1.57); H-2″ (δ_H_ 1.57)/H-3″ (δ_H_ 1.33); H-3″ (δ_H_ 1.33)/H-4″ (δ_H_ 1.32); and H-4″ (δ_H_ 1.32)/H-5″ (δ_H_ 0.88). Compound **1** was confirmed by the analysis of the HMBC spectrum showing correlations from H-7 (δ_H_ 1.52) to C-6 (δ_C_ 68.8), C-1 (δ_C_ 91.3), and C-5 (δ_C_ 34.7); H-1 (δ_H_ 4.34) to C-6 (δ_C_ 68.8), C-3 (δ_C_ 48.2), and C-5 (δ_C_ 34.7); H-2 (δ_H_ 3.44) to C-8 (δ_C_ 152.9) and C-6′ (δ_C_ 160.2); H-10 (δ_H_ 1.83) to C-3 (δ_C_ 48.2), C-8 (δ_C_ 152.9), and C-9 (δ_C_ 111.6); H-2′ (δ_H_ 6.41) to C-1′ (δ_C_ 155.4), C-4′ (δ_C_ 105.6), and C-6′ (δ_C_ 117.0); H-1″ (δ_H_ 2.92) to C-2′ (δ_C_ 114.1), C-3′ (δ_C_ 149.6), and C-4′ (δ_C_ 105.6) as shown in Figure 2. In addition, in the ^13^C NMR spectrum appeared a signal resonance at δ_C_ 164.8 ppm. This signal is not observed in the interacted HMBC spectrum. Therefore, a suggestion that a ring-closing ester for positions 4′ and 5′ to match the molecular formula. Regarding the configuration of **1**, the NOESY spectrum was analyzed for observations such as H-7 (δ_H_ 1.52) correlated to H-1 (δ_H_ 4.34), suggesting that these protons are the same *β*-orientation. While the opposite *α*-orientation of H-2 (δ_H_ 3.44) correlated to H-9 (δ_H_ 5.06) and H-10 (δ_H_ 1.83) (Figure 3). From these data above, the structure of **1** was defined as hexahydro-1,6-dihydroxy-6-methyl-9-(1-methylethenyl)-3′-pentyl-dibezenzofuran-4′-oxo-5′-oxabicyclo and named cannabielsoxa, a new cannabinoid.

Compound **2** was obtained as a black amorphous powder. The molecular formula of **2** was determined as C_43_H_52_N_4_O_6_ by observing the HREIMS spectrum with a peak at *m/z* 721.3988 (calcd [M + H]^+^ 721.3960). The ^1^H and ^13^C NMR data of **2** (Table 2) were compared to those of compounds **3**, **4**, and **5**, suggesting that **2** belongs to chlorin derivatives. The methyl proton signals at 1.69 (3H, *t*, *J* = 7.6 Hz), 1.81 (3H, *d*, *J* = 7.6 Hz), 3.24 (3H, *s*), 3.41 (3H, *s*), and 3.69 (3H, *s*), together with methine resonances at 4.30 (1H, *m*), 4.50 (1H, *dd*, *J* = 7.6, 1.6 Hz), 8.01 (1H, *dd*, *J* = 18.0, 12.0 Hz), 8.55 (1H, *s*), 9.38 (1H, *s*, H-5), and 9.49 (1H, *s*), were assigned to the chlorin skeleton. The proton signals at 1.16 (3H, *t*, *J* = 6.8 Hz), 2.28 (2H, *m*), 2.54 (2H, *m*), 4.04 (2H, *dd*, *J* = 6.8, 2.4 Hz), and a carbonyl carbon at δ_C_ 173.2 were assigned to the butyric acid, ethyl ester moiety. Similarly, the resonance signals at 0.88 (3H, *t*, *J* = 6.4 Hz), 1.24–1.30 (*m*), 1.64 (2H, *m*), 2.35 (2H, *t*, *J* = 7.6 Hz), and a carbonyl group at δ_C_ 178.3 were assigned to the octanoic acid moiety. The structure of **2** is also determined by 2D NMR (HMQC, COSY, and HMBC spectra) as shown in Figure 2. The HMBC correlation between the methylene proton (δ_H_ 2.28) and methine carbon (δ_C_ 51.8) along with COSY correlations confirmed that the butyric acid, ethyl ester moiety is attached at the C-17 position. The octanoic acid moiety is suggested as the connection to the C-13^2^ position. This matches the molecular formula as well as an ion peak at *m/z* 721.3988 observed by the HREIMS spectrum. In addition, the absence of the carbon signal at the C-13^2^ position may be affected by the shielding of the chain of the octanoic acid moiety. From the above evidence, compound **2** was identified as 17-phorbinebutyric acid, 3-ethenyl-8-ethyl-13^2^-octanoic acid-13^2^-hydroxy-2,7,12,18-tetramethyl-13-oxo, ethyl ester, designated 13^2^-hydroxypheophorbide c ethyl ester, a new derivative of chlorin-type compounds.

Compound **3** was afforded as a black amorphous powder. The HREIMS spectrum gave a peak at *m/z* 570.4541 [M + Na]^+^, corresponding to the molecular formula C_34_H_36_N_4_O_3_. The ^1^H, ^13^C NMR and DEPT spectra of **3** displayed five methyl resonances at δ_H_: 1.69 (3H, *t*, *J* = 7.6 Hz, H-8^2^), 1.81 (3H, *d*, *J* = 7.6 Hz, H-18^1^), 3.24 (3H, *s*, H-7^1^), and 3.41 (3H, *s*, H-2^1^); five methylenes at δ_H_: 2.25–2.34 (2H, *m*, H-17^2^); 2.56 (1H, *m*) and 2.69 (1H, *m*), H-17^1^; 3.69 (2H, *q*, *J* = 7.6 Hz, H-8^1^); 5.13 (1H, *d*, *J* = 20.0 Hz) and 5.24 (1H, *d*, *J* = 20.0 Hz, H-13^2^); 6.18 (1H, *dd*, *J* = 11.6; 1.2 Hz) and 6.30 (1H, *dd*, *J* = 18.0; 1.2 Hz). six methines at 4.29 (1H, *dd*, *J* = 8.4, 2.4 Hz, H-17); 4.48 (1H, *dd*, *J* = 7.2, 1.6 Hz, H-18); 8.01 (1H, *dd*, *J* = 18.0, 12.0 Hz, H-3^1^), 8.55 (1H, *s*, H-20); 9.38 (1H, *s*, H-5); and 9.50 (1H, *s*, H-10). The structure of **3** was identified by 2D NMR (HMQC, COSY, and HMBC spectra). The HMBC data showed cross-peaks from H-2^1^ (δ_H_ 3.41) to carbons at C-1 (141.7), C-2 (131.7), and C-3 (136.0); from H-7^1^ (δ_H_ 3.24) to carbons at C-6 (155.4), C-7 (136.4), and C-8 (145.2); from H-8^2^ (δ_H_ 1.69) to carbons at C-8^1^ (19.6) and C-8 (145.2); and from H-12^2^ (δ_H_ 3.67) to carbons at C-11 (138.0), C-12 (130.6), and C-13 (128.5), which suggested methyl group positions in the structure of **3**. In addition, the HMBC spectrum also exhibited correlations from H-5 (δ_H_ 9.38) to carbons at C-3 (136.0) and C-7 (136.4); H-10 (δ_H_ 9.50) to C-8 (145.2) and C-11 (138.0); H-20 (δ_H_ 8.55) to carbons at C-2 (131.7) and C-18 (50.1), which leads to confirming a chlorine skeleton. One methoxy was attached at the C-17^3^ (173.6) position by observed HMBC cross-peaks from the signal at δ_H_ 3.61 to carbon at C-17^3^ (173.6). Similarly, at the C-3 position, it was also determined to attach a vinyl group based on HMBC and COSY correlations (see in Supplementary Materials). From these data above, the structure of **3** was defined as methyl pyropheophorbide A [23].

Compound **4** was obtained as a dark green powder. The molecular formula was determined as C_37_H_38_N_4_O_7_ based on HRESIMS analysis (obsd. [M + H]^+^, *m/z* 651.2808; calc. [M + H]^+^, *m/z* 615.2813). The ^1^H and ^13^C NMR spectra of **4** were very similar to those of **3** (Table 2), except that the presence of a CHO group at C-7 was detected by HMQC and HMBC correlations. The difference in the NMR data of **4** and **3** also indicated the disappearance of the proton signals of the methylene group and a change in the nature of the hydroxy and methyl ester groups in **4** compared to **3**. Similarly, the ethyl ester signals instead of a methoxy signal at C-17^3^. In addition, the structure of **4** was also thoroughly analyzed using 2D NMR (COSY, HMQC, and HMBC (see in Supplementary Materials)) and compared to the previous report [24]. Therefore, compound **4** was identified as 13^2^-hydroxypheophorbide b ethyl ester.

Compound **5** was isolated as a dark green amorphous solid. The HRESIMS spectrum revealed that compound **5** had a molecular formula of C_37_H_40_N_4_O_7_ with an observed *m*/*z* of 653.2979 [M + H]^+^, calc. for a [M + H]^+^ of 653.2970). A comparison of the NMR data of compound **5** with those of compounds **3** and **4** indicated structural similarity; however, there was a minor difference observed at positions C-13^1^ and C-13^2^. The change in significant shifts of C-13^1^ from δ_C_ 196.4 to 102.1 and C-13^2^ from δ_C_ 89.0 to 100.6 suggested that both positions must be oxygenated, likely forming a C-O-O-C functionality to match the molecular formula. The structure of **5** was also analyzed by 2D NMR (COSY, HMQC, and HMBC (see in Supplementary Materials)) and compared to ligulariaphytin A, which was isolated from *Ligularia knorringiana* [25], leading to the conclusion that the structure of **5** is ligulariaphytin A.

Similarly, compounds **6**–**11** were also identified by using NMR data and HRESIMS spectra (see in Supplementary Materials). Their structures were determined as cannabidiolic acid [26], cannabidiolic acid methyl ester [27], cannabidiol [26], delta-8-tetrahydrocannabinol [28], cannabichromene [29], and dihydroxy-cannabidiol [30]. These identifications were made by comparing their experimental spectroscopic and physicochemical data with previously reported literature. The corresponding structures are presented in Figure 1.

Cannabidiolic acid (**6**): ^1^H NMR (400 MHz, CDCl_3_):δ_H_ (ppm): 4.13 (1H, *dd*, *J* = 14.0, 6.8 Hz, H-1), 5.57 (1H, *s*, H-2), 2.09–2.20 (2H, *m*, H-4), 1.82 (2H, *m*, H-5), 2.38 (1H, *m*, H-6), 1.79 (3H, *s*, H-7), 4.40 (1H, *s*, H-9a) and 4.54 (1H, *t*, *J* = 3.0 Hz, H-9b), 1.72 (3H, *s*, H-10), 6.26 (1H, *s*, H-5′), 2.83–2.92 (2H, *m*, H-1″), 1.58 (2H, *m*, H-2″), 1.33 (2H, *m*, H-3″), 1.32 (2H, *m*, H-4″), 0.89 (3H, *t*, *J* = 6.8 Hz, H-5″); ^13^C NMR (100 MHz, CDCl_3_): δ_C_ (ppm): 35.5 (C-1), 124.0 (C-2), 140.7 (C-3), 30.3 (C-4), 27.9 (C-5), 46.8 (C-6), 23.9 (C-7), 147.3 (C-8), 111.5 (C-9),19.0 (C-10), 114.6 (C-1′), 164.3 (C-2′), 102.7 (C-3′), 147.7 (C-4′), 112.1 (C-5′), 161.1 (C-6’), 36.7 (C-1″), 31.4 (C-2″), 32.1 (C-3″), 22.7 (C-4″), 14.2 (C-5″), 176.4 (CO); ESI-MS: *m/z* at 359.2228 [M + H]^+^.

Cannabidiolic acid methyl ester (**7**): ^1^H NMR (400 MHz, CDCl_3_):δ_H_ (ppm): 4.10 (1H, *d*, *J* = 6.0 Hz, H-1), 5.57 (1H, *s*, H-2), 2.09–2.23 (2H, *m*, H-4), 1.82 (2H, *m*, H-5), 2.40 (1H, *m*, H-6), 1.79 (3H, *s*, H-7), 4.40 (1H, *s*, H-9a) and 4.54 (1H, *t*, *J* = 2.0 Hz, H-9b), 1.72 (3H, *s*, H-10), 6.26 (1H, *s*, H-5’), 2.83–2.92 (2H, *m*, H-1″), 1.58 (2H, *m*, H-2″), 1.33 (2H, *m*, H-3″), 1.32 (2H, *m*, H-4″), 0.90 (3H, *t*, *J* = 6.8 Hz, H-5″), 3.50 (3H, *s*, -OCH_3_); ^13^C NMR (100 MHz, CDCl_3_): δ_C_ (ppm): 35.5 (C-1), 124.0 (C-2), 140.5 (C-3), 30.3 (C-4), 27.9 (C-5), 46.8 (C-6), 23.8 (C-7), 147.3 (C-8), 111.4 (C-9), 19.0 (C-10), 114.6 (C-1’), 164.4 (C-2’), 102.9 (C-3’), 147.7 (C-4’), 112.1 (C-5’), 161.0 (C-6’), 36.7 (C-1″), 31.4 (C-2″), 32.1 (C-3″), 22.6 (C-4″), 14.2 (C-5″), 176.4 (CO), 50.8 (OCH_3_); ESI-MS: *m/z* at 373.2368 [M + H]^+^.

Cannabidiol (**8**): ^1^H NMR (400 MHz, CD_3_OD):δ_H_ (ppm): 3.92 (1H, *m*, H-1), 5.28 (1H, *s*, H-2), 1.27 and 1.73 (2H, *m*, H-4), 2.00 and 2.18 (2H, *m*, H-5), 2.89 (1H, *m*, H-6), 1.67 (3H, *s*, H-7), 4.20 (1H, *m*, H-9a) and 4.45 (1H, *d*, *J* = 2.0 Hz, H-9b), 1.62 (3H, *s*, H-10), 6.08 (2H, *s*, H-3’ and 5’), 2.37 (2H, *m*, H-1″), 1.56 (2H, *quintet*, H-2″), 1.28 (2H, *m*, H-3″), 1.30 (2H, *m*, H-4″), 0.88 (3H, *t*, *J* = 6.8 Hz, H-5″); ^13^C NMR (100 MHz, CD_3_OD): δ_C_ (ppm): 37.5 (C-1), 127.3 (C-2), 134.2 (C-3), 30.7 (C-4), 31.7 (C-5), 46.3 (C-6), 23.7 (C-7), 150.4 (C-8), 110.5 (C-9), 19.5 (C-10), 115.9 (C-1′), 157.3 (C-2′), 108.3 (C-3′), 142.7 (C-4′), 108.3 (C-5′), 157.5 (C-6′), 36.6 (C-1″), 32.0 (C-2″), 32.6 (C-3″), 23.6 (C-4″), 14.4 (C-5″); ESI-MS: *m/z* at 315.2326 [M + H]^+^.

Delta-8-tetrahydrocannabinol (**9**): ^1^H NMR (400 MHz, CDCl_3_):δ_H_ (ppm): 6.26 (1H, *s*, H-2), 6.28 (1H, *s*, H-4), 1.90 (1H, *m*, H-6a), 1.67 (2H, *m*, H-7), 5.65 (1H, *brs*, H-8), 2.09 (2H, *m*, H-10), 3.85 (1H, *m*, H-10a), 1.80 (3H, *s*, H-11), 1.24 (6H, *s*, H-12 and H-13), 2.43 (2H, *t*, *J* = 7.2 Hz, H-1′), 1.56 (2H, *m*, H-2′), 1.32 (2H, *m*, H-3′), 1.31 (2H, *m*, H-4′), 0.87 (3H, *t*, *J* = 6.8 Hz, H-5′); ^13^C NMR (100 MHz, CDCl_3_): δ_C_ (ppm): 154.2 (C-1), 109.7 (C-2), 143.6 (C-3), 109.4 (C-4), 156.2 (C-5), 75.1 (C-6), 48.5 (C-6a), 23.4 (C-7), 123.9 (C-8), 140.0 (C-9), 28.5 (C-10), 33.0 (C-10a), 115.1 (C-10b), 23.8 (C-11), 26.0 (C-12), 29.7 (C-13), 35.6 (C-1′), 30.8 (C-2′), 31.7 (C-3′), 22.7 (C-4′), 14.2 (C-5′); ESI-MS: *m/z* at 315.2233 [M + H]^+^.

Cannabichromene (**10**): ^1^H NMR (400 MHz, CDCl_3_):δ_H_ (ppm): 6.12 (1H, *s*, H-2), 6.25 (1H, *s*, H-4), 5.50 (1H, *d*, *J* = 10.2 Hz, H-7), 6.63 (1H, *d*, *J* = 10.2 Hz, H-8), 1.71 (2H, *m*, H-10), 2.08 (2H, *m*, H-11), 5.09 (1H, *t*, *J* = 7.6 Hz, H-12), 1.58 (3H, *s*, H-14), 1.66 (3H, *s*, H-15), 1.38 (3H, *s*, H-16), 2.45 (2H, *t*, *J* = 7.8 Hz, H-1′), 1.56 (2H, *m*, H-2′), 1.31 (2H, *m*, H-3′), 1.30 (2H, *m*, H-4′), 0.87 (3H, *t*, *J* = 6.8 Hz, H-5′); ^13^C NMR (100 MHz, CDCl_3_): δ_C_ (ppm): 107.2 (C-1), 107.8 (C-2), 144.9 (C-3), 109.2 (C-4), 154.2 (C-5), 78.3 (C-6), 127.3 (C-7), 117.0 (C-8), 151.2 (C-9), 41.2 (C-10), 22.8 (C-11), 124.4 (C-12), 131.8 (C-13), 17.7 (C-14), 25.8 (C-15), 26.4 (C-16), 36.0 (C-1′), 30.7 (C-2′), 31.6 (C-3′), 22.7 (C-4′), 14.2 (C-5′); ESI-MS: *m/z* at 315.2305 [M + H]^+^.

Dihydroxy-cannabidiol (**11**): ^1^H NMR (400 MHz, CDCl_3_):δ_H_ (ppm): 3.34 (1H, *dd*, *J* = 10.8, 6.0, H-1), 4.11 (1H, *d*, *J* = 6.0, H-2), 1.49 (2H, *m*, H-4), 1.70 (2H, *m*, H-5), 1.88 (1H, *m*, H-6), 1.48 (3H, *s*, H-7), 5.06 (2H, *d*, *J* = 12.4, H-9), 1.82 (3H, *s*, H-10), 6.27 (1H, *s*, H-3′), 6.29 (1H, *s*, H-5’), 2.49 (2H, *m*, H-1″), 1.57 (2H, *m*, H-2″), 1.32 (2H, *m*, H-3″), 1.31 (2H, *m*, H-4″), 0.88 (3H, *t*, *J* = 7.2 Hz, H-5″); ^13^C NMR (100 MHz, CDCl_3_): δ_C_ (ppm): 42.1 (C-1), 89.4 (C-2), 69.4 (C-3), 29.8 (C-4), 34.8 (C-5), 48.5 (C-6), 28.3 (C-7), 152.1 (C-8), 111.5 (C-9), 22.8 (C-10), 116.9 (C-1′), 160.1 (C-2′), 109.8 (C-3′), 144.9 (C-4′), 108.3 (C-5′), 160.1 (C-6′), 36.2 (C-1″), 31.1 (C-2″), 31.6 (C-3″), 22.8 (C-4″), 14.2 (C-5″); ESI-MS: *m/z* at 349.2370 [M + H]^+^.

### 2.2. Bioactivity Results

**Figure 4:**
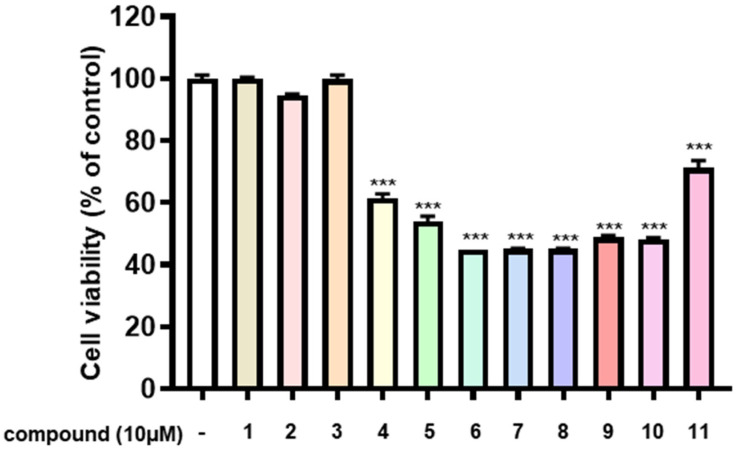
Antitumor effects of compounds from *C. sativa* on SK-N-SH neuroblastoma cells. SK-N-SH cells were seeded into 96-well plates at a density of 5 × 10^4^ cells/mL and treated with the concentration of the compounds at 10 μM for 24 h. Cell viability was determined using the MTT assay, as described in the ‘Methods’ section. Data represent the means ± standard deviation (S.D.) (*n* = 3). Statistical significance was determined by one-way ANOVA with Tukey’s post hoc test, and differences were statistically significant at *** *p* < 0.001 compared to DMSO-treated controls.

**Figure 5:**
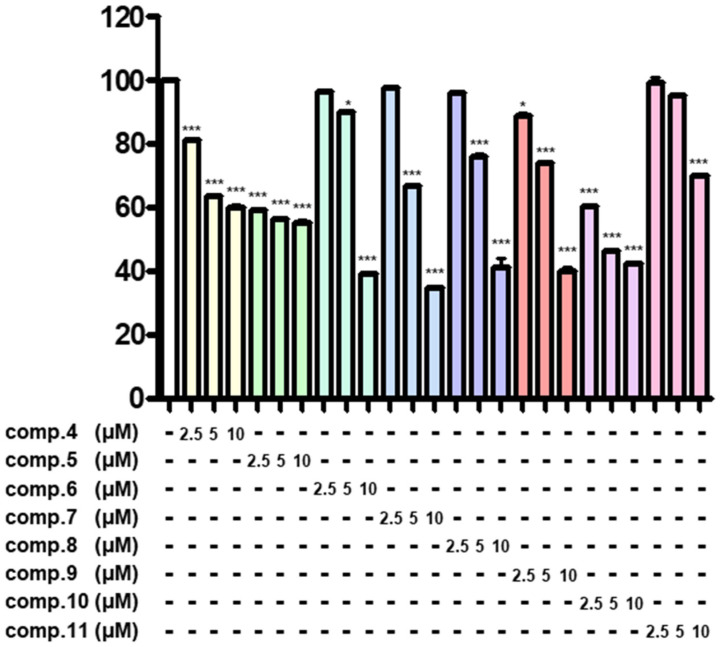
Antitumor effects of compounds on SK-N-SH neuroblastoma cells. SK-N-SH cells were seeded into 96-well plates at a density of 5 × 10^4^ cells/mL and treated with compounds at concentrations of 2.5, 5, and 10 μM for 24 h. Cell viability was assessed using the MTT assay, as described in the ‘Methods’ section. Data represent the means ± standard deviation (S.D.) (*n* = 3). Statistical significance was determined by one-way ANOVA with Tukey’s post hoc test, and differences were statistically significant at * *p* < 0.05 and *** *p* < 0.001 compared to DMSO-treated controls.

**Figure 6:**
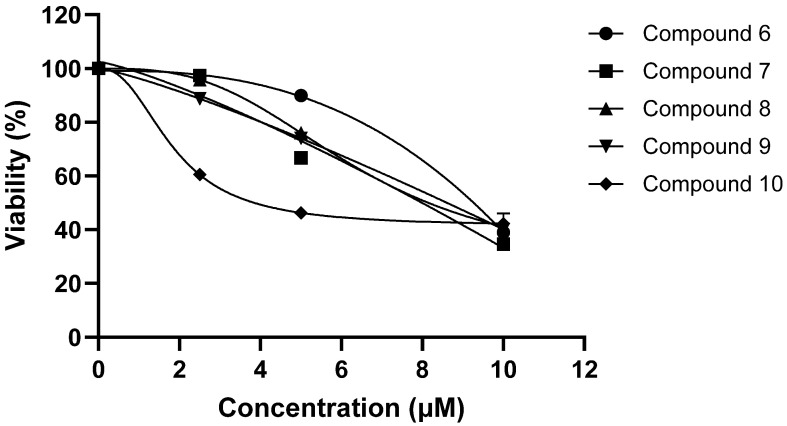
IC_50_ values of compounds **6**–**10**.

Neuroblastomas, a solid tumor in the nervous system, are the most common neoplasms in children [31,32,33]. To test whether the purified compounds (**1**–**11**) from the flowers of *C. sativa* (Figure 1) could have antitumor effects against neuroblastomas, we measured their inhibitory activity on cell viability in SK-N-SH neuroblastoma cells using the MTT assay. The results of the initial preliminary evaluation revealed the compounds **2** and **4**–**10** suppressed the proliferation of SK-N-SH cells at concentrations of 10 μM (Figure 4), whereas compounds **1**, **3,** and **11** were found inactive under the same experimental conditions. Here, we found an interesting discovery: cannabinoids (**6**–**10**) that have double bonds at the hexane ring in their structure have stronger inhibitory activity against neuroblastoma cells than the remaining compounds that are missing double bonds (**1** and **11**). Similarly, the chlorin-type compounds **2** and **3**, which lack an acetate substituent at position C-13^2^ exhibit a reduced inhibitory effect on neuroblastoma cells compared to compounds **4** and **5**. The cannabinoids (**6**–**10**) have stronger effects than chlorin-type compounds (**4**, **5**). These compounds exhibit the most potent inhibition against the proliferation of SK-N-SH cells. Therefore, we further evaluated the antitumor effects of these compounds at lower concentrations. The results showed that except for compound **11**, the remaining compounds (**4**–**10**) displayed a significant inhibition at 5 μM and maximal inhibitory activity by 10 μM (Figure 5). The data provided in Figure 6 also indicate the IC_50_ values of cannabinoids (**6**–**10**) on neuroblastoma cells at lower than 10 μM.

### 2.3. Molecular Docking Studies

**Figure 7:**
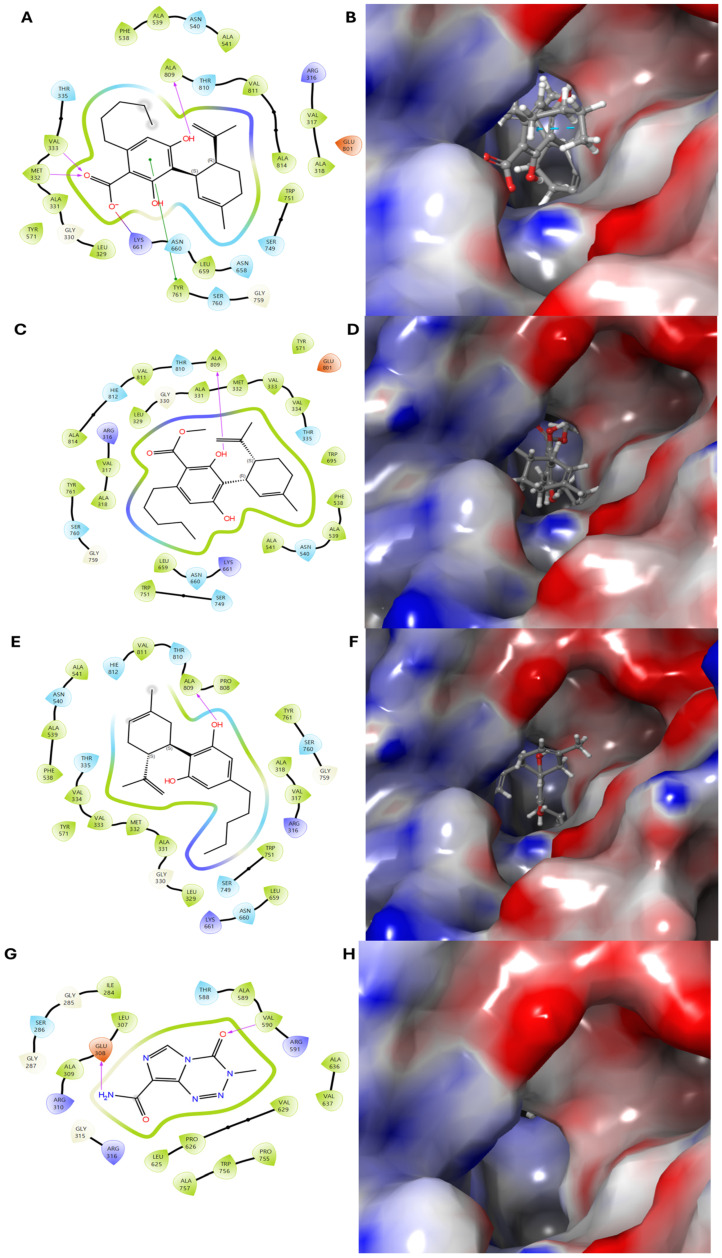
Molecular docking simulation of compounds **6**–**8** and the positive control with the human neuroblastoma SK-N-SH protein (PDB ID: 4KUM). (**A**,**B**) Two-dimensional and three-dimensional binding interactions of compound **6** with 4KUM. (**C**–**F**) Structural and interaction analyses of compounds **7** and **8**, respectively. (**G**,**H**) Binding interactions of the positive control with 4KUM. Compounds **6**–**8** have transparent surfaces on their ligand-binding pockets (left panels). The right panels depict interactions between the ligands and the protein’s amino acid residues, such as hydrogen bonds (pink) and pi–pi stacking interactions (green).

To investigate the mechanisms underlying the anticancer activity of the isolated compounds against human neuroblastoma, molecular docking simulations were performed using Maestro v.13.4.134 (Schrödinger software, MMshare v.6.0.134) [34,35,36]. The crystal structure of the neuroblastoma target protein (PDB ID: 4KUM) was obtained from the Protein Data Bank [37,38]. Among the tested compounds, compounds **6**, **7**, and **8**, which displayed the highest bioactivity, were docked into the active site of 4UKM. The in silico results are in strong agreement with the in vitro experiments. In particular, the docking results revealed that compounds **6**–**8** exhibited strong binding affinity, with docking scores of −5.878, −5.878, and −6.185 kcal/mol, suggesting significant interaction stability compared to the positive control, temozolomide. Key residues, including ALA809 and VAL333, were found to play critical roles in stabilizing the active conformation of 4UKM (Figure 7). Moreover, based on their high binding affinity, hydrogen bond interactions, and efficient occupation of the ligand-binding pocket, compounds **6**–**8** demonstrate strong potential as inhibitors with anticancer activity against human neuroblastoma, comparable to temozolomide, an approved drug [39].

### 2.4. ADMET Studies

ADMET studies (Absorption, Distribution, Metabolism, Excretion, and Toxicity) assess the pharmacokinetic and toxicological properties of compounds, which are crucial for drug development [40]. SwissADME, an online tool, provides in silico predictions of physicochemical properties, lipophilicity, solubility, drug-likeness, and pharmacokinetics, facilitating the early-stage screening of drug candidates [41]. Compounds **6**, **7**, and **8** exhibited acceptable drug-like properties and other drug-likeness parameters based on standard rules derived from in vitro and in silico studies. The log S values for compounds **6, 7**, and **8** were −5.93, −6.15, and −5.54, respectively, indicating that these compounds exhibit acceptable solubility as drug-like molecules [42]; they can be considered suitable for physiological environments [43]. Additionally, the TPSA values suggested promising potential for effective intestinal absorption. Furthermore, compounds with nRotB values below 10 indicate favorable stereochemical characteristics, enhancing their potential for oral bioavailability. In Table 3, compounds **6**–**8** exhibit high GI absorption, suggesting these compounds are likely to be well absorbed when administered orally. A comparison with the BBB-permeable drug Gabapentin suggests that compound **8** is expected to cross the blood–brain barrier (BBB) and be absorbed in the gastrointestinal tract [44]. None of the compounds are substrates of P-gp (P-glycoprotein), an efflux membrane transporter that can limit drug accumulation in cells, including those in the brain [45].

Cytochrome P450 (CYP) enzymes play a crucial role in human drug metabolism of numerous medications and natural compounds [46,47]. Inhibiting these enzymes can lead to significant clinical consequences, primarily through drug–drug interactions (DDIs). When a CYP enzyme is inhibited, the metabolism of co-administered drugs that are substrates of the same enzyme may be reduced [48]. This metabolic decrease can result in elevated plasma concentrations of the affected drugs, potentially leading to increased toxicity or enhanced pharmacological effects [49]. CYP2C9 is responsible for metabolizing various therapeutic agents. Inhibition of CYP2C9 can decrease the clearance of drugs, leading to elevated plasma levels and an increased risk of adverse effects such as bleeding or hypoglycemia [50,51]. Inhibition of CYP3A4 can result in higher plasma concentrations of its substrates, potentially causing toxicity [52,53]. CYP2D6 is involved in the metabolism of various drugs, including beta-blockers like metoprolol. Inhibition of CYP2D6 can lead to increased plasma concentrations of drugs, potentially causing adverse effects such as bradycardia or hypotension [54]. The SwissADME prediction indicated that compounds **6** and **7** inhibit CYP2C9 and CYP3A4, while compound **8** also inhibits CYP2D6. High concentrations of these compounds may be harmful to the human body, as reflected in their “Caution” classification (Table 3 and Table 4).

*C. sativa* is known as a herbal medicine with the chemical constituents found for the classes of secondary metabolites as cannabinoid phenols, non-cannabinoid phenols, flavonoids, terpenoids, steroids, and alkaloids [5]. However, so far there have been no reports of chlorin-type compounds isolated from this plant. In this work, we have presented further details on a method for separating chlorin-type compounds together with a preliminary evaluation for antitumor effects against neuroblastomas. In addition, the previous studies also disclosed that biologically effective studies focused mainly on the cannabinoids group, such as cannabidiol (CBD) for anticonvulsive, anti-inflammatory, antioxidant, and antipsychotic effects; tetrahydrocannabinol (THC) for antioxidant, antipruritic, and anti-inflammatory; cannabichromene (CBC) for anti-inflammatory and analgesic; cannabigerol (CBG) for antifungal effects, anticancer, antidepressant, analgesic, and antiarrhythmic effects; cannabinol (CBN) for anticonvulsant, anti-inflammatory, antibiotic, and anti-MRSA activity; cannabidiolic acid (CBDA) for antiemetic effects; cannabidavarin (CBDV) for anticonvulsant properties and antiemetic properties; tetrahydrocannabinoilic acid (THCA) for immunomodulatory, anti-inflammatory, neuroprotective, antineoplastic activity, and antiemetic effects; tetrahydrocannabivanrin (THCV) for anticonvulsant [5]. These findings highlight the broad therapeutic potential of cannabinoids. However, prior scientific studies on *C. sativa* have been limited, particularly concerning its antitumor effects. Specifically, neuroblastoma remains an underexplored area where new treatment strategies are urgently needed. In this study, the compounds isolated from the flower of *C. sativa* were investigated for their potential cytotoxic effects on neuroblastoma cells. A chemical investigation resulted in the isolation of one new cannabinoid (**1**) and one new chlorin-type metabolite (**2**), along with six known cannabinoids (**6**–**11**), and the first isolation of chlorin-type compounds: pyropheophorbide A (**3**), 13^2^-hydroxypheophorbide b ethyl ester (**4**), and ligulariaphytin A (**5**). Their structures were elucidated using spectroscopic data. The isolated compounds were evaluated for their antitumor activity against SK-N-SH neuroblastoma cells. Furthermore, the underlying mechanisms of action against human neuroblastoma cancer cells were investigated through in silico methods using molecular docking simulations. These results provide valuable scientific data that will help the research orientations of the next studies on *C. sativa*.

Neuroblastoma is the most common solid tumor in children and the most frequent malignancy in the first year of life [55]. Most primary tumors detected in the abdomen are caused by the adrenal medulla, with a 65% ratio [56]. The death rate for children with severe illness is still significant, and there are few effective therapeutic options available. Standard therapy for advanced diseases includes chemotherapy, surgery, and radiation. However, many patients who do not respond to these modalities will find it difficult in treatment [55]. Therefore, the development of a new drug and safety is necessary. In this study, we demonstrated cytotoxicity in neuroblastoma cells by cannabinoids (**6**–**11**) and chlorin derivatives (**4** and **5**). This study is an initial step toward developing a product for the treatment of neuroblastoma because CBD and CBDA are major compounds with high content in *C. sativa* [57]. A part of our results is consistent with previous reports indicating that CBD has potential as an inhibitor of neuroblastoma cell proliferation in both in vitro and in vivo studies [58]. This reinforces confidence that a *cannabis* extract enriched in cannabinoids has the potential to be a promising candidate for neuroblastoma treatment [8,58]. However, we also suggest that in vivo and clinical studies should be conducted to confirm the therapeutic potential of the remaining cannabinoids and chlorin derivatives in neuroblastoma cancer. In South Korea, strict regulations surrounding *cannabis* limited our ability to conduct additional in vivo activity studies. Nevertheless, in the era of artificial intelligence, molecular docking has emerged as a powerful tool for investigating and predicting the pharmacological effects of compounds, offering valuable insights even in the absence of extensive experimental validation.

## 3. Materials and Methods

### 3.1. General Experimental Procedures

Column chromatography was carried out employing normal phase (Kiesel gel 60, 70–230 mesh, Merck, Darmstadt, Germany) and reversed phase (C18). Nuclear magnetic resonance (NMR) spectra (1D and 2D) were recorded by using a JEOL JNM ECP-400 spectrometer (JEOL Ltd., Tokyo, Japan). HR-MS data were recorded by using an electrospray ionization (ESI) quadrupole time-of-flight (Q-TOF) tandem mass spectrometry (MS/MS) system (6530 accurate-mass Q-TOF MS, Agilent Technologies, Burladinge, Germany).

### 3.2. Plant Materials

The flowers of *C. Sativa* were collected by Nongboo mind Co. Ltd. Goyang-si, Korea in 2023, identified by Prof. Chul Min Kim (Division of Horticulture Industry, Wonkwang University). A voucher specimen (WKU-25) was deposited at the Pharmacognosy Laboratory of the College of Oriental Medicine, Wonkwang University, Chon-bukllo, Korea.

### 3.3. The Isolation of Compounds

**Scheme 1:**
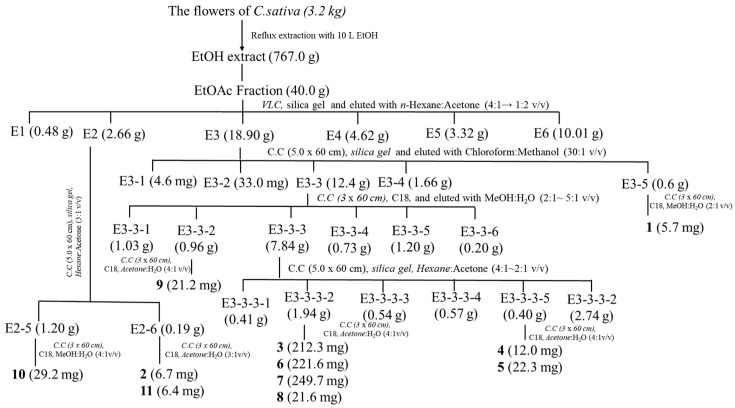
Extraction and isolation for compounds **1**–**11**.

The isolation of compounds was carried out as follows: dried flowers of *C. sativa* (3.2 kg) were extracted twice with ethanol (10 L × 2) at room temperature, and the extract was concentrated to yield a brownish slurry (767.0 g). The ethanol extract was then suspended in water (1 L) and partitioned with *n*-hexane (3 L × 3) and ethyl acetate (3 L × 3), resulting in four extracts totaling 311.4 g and 40.0 g, respectively. The ethyl acetate fraction (40.0 g) was further subjected to silica gel column *vacuum* liquid chromatography (*VLC*) and eluted with a gradient of *n*-hexane: acetone (4:1 → 1:2) to obtain seven fractions (Fr. E1–E6). The detailed isolation of the compounds is shown in Scheme 1.

### 3.4. Cell Culture

Human neuroblastoma SK-N-SH cells were obtained from the Korean Cell Line Bank (Seoul, Korea). The cells were cultured in Minimal Essential Medium (MEM; Gibco, Grand Island, NY, USA) supplemented with 10% heat-inactivated fetal bovine serum (FBS; Gibco, Grand Island, NY, USA), 100 units/mL penicillin G, and 100 mg/mL streptomycin (Gibco, Grand Island, NY, USA) at 37 °C in a humidified atmosphere containing 5% CO_2_. Cells were seeded into 96-well plates at the density of 5 × 10^4^ cells/mL. All experiments were conducted 24 h after cell seeding.

### 3.5. Cell Viability Assay

Cell viability was assessed in a 96-well plate using a colorimetric 3-[4,5-dimethylthiazol-2-yl]-2,5-diphenyltetrazolium bromide (MTT; Duchefa Biochemie, BH, Haarlem, The Netherlands) assay, which measures mitochondrial activity in living cells. Briefly, SK-N-SH cells were incubated with various concentrations of the compounds (2.5–10 μM) or dimethyl sulfoxide (DMSO; Sigma-Aldrich, St. Louis, MO, USA) for 24 h, followed by incubation with MTT (1 mg/mL) for 3 h at 37 °C. After incubation, the MTT solution was gently removed, and 100 μL of DMSO was added to each well to dissolve the dark-blue formazan crystals formed by viable cells. Absorbance was measured at 540 nm using a microplate ELISA reader (BioTek Epoch, Winooski, VT, USA). Cell viability in DMSO-treated cells was defined as 100%.

### 3.6. Statistical Analysis

Data were analyzed using GraphPad Prism 4 software, and significant differences were analyzed using Tukey’s one-way analysis of variance. Data are presented as the mean and variance of at least three independent experiments. Differences were statistically significant at * *p* < 0.05 and *** *p* < 0.001 compared to DMSO-treated controls.

### 3.7. Molecular Docking Simulations

The three-dimensional (3D) structure of the human neuroblastoma SK-N-SH protein (PDB ID: 4KUM) was obtained from the Protein Data Bank (https://www.rcsb.org/ (accessed on 15 December 2024)) for in silico analysis [59]. The protein structure was optimized using the OPLS4 force field until the root-mean-square deviation (RMSD) of the heavy atoms averaged 0.3 Å. Two-dimensional (2D) ligand structures were prepared and converted into optimized 3D geometries at pH 7.0 ± 2.0 using the LigPrep tool, which also established ligand chirality based on their 3D configurations. The final stage of LigPrep involved energy minimization of the 3D conformers with the OPLS4 force field. Molecular docking and energy calculations were conducted using Glide in extra precision (XP) mode.

## 4. Conclusions

A chromatographic method was used for the isolation of the compounds from the EtOAc extract of the flowers of *C. sativa*. This study successfully isolated a new cannabinoid (**1**) and six known cannabinoid compounds (**6**–**11**), along with a new chlorin-type compound (**2**) and three additional chlorine-type compounds (**3**–**5**), which were reported for the first time from the flowers of *C. sativa*. All compounds were also investigated for their antitumor effects against SK-N-SH neuroblastoma cells as measured by the MTT assay. Among them, compounds **4**–**10** revealed strong inhibitory activity at concentrations of 5 and 10 μM. These results provided the elementary data for further research into the antitumor mechanisms of cannabinoids and chlorin derivatives in neuroblastoma models that are both in vitro and in vivo.

## Figures and Tables

**Table 1:** ^1^H and ^13^C NMR data of compound **1** [*δ*_H,_ mult. (*J* in Hz)].

Position	^1^H [*δ*_H,_ Mult. (*J* in Hz)]	^13^C (*δ*_C_)
**1**	4.34, *d* (5.6)	91.3
**2**	3.44, *dd* (11.2, 5.6)	41.6
**3**	1.89, *m*	48.2
**4**	1.56, *m*	31.2
**5**	1.74, *m*	34.7
**6**	-	68.8
**7**	1.52, *s*	28.2
**8**	-	152.9
**9**	5.06, *d* (3.6)	111.6
**10**	1.83, *s*	22.7
**1′**	-	155.4
**2′**	6.41, *s*	114.1
**3′**	-	149.6
**4′**	-	105.6
**5′**	-	160.2
**6′**	-	117.0
**7′**	-	164.8
**1″**	2.92, *m*; 3.03, *m*	34.8
**2″**	1.57, *m*	25.7
**3″**	1.33, *m*	32.1
**4″**	1.32, *m*	22.6
**5″**	0.88, *t* (6.8)	14.2

Measured in CDCl_3_. The assignments were determined by DEPT, COSY, HSQC, HMBC, and NOESY experiments.

**Table 2:** ^1^H and ^13^C NMR data of compounds **2**–**5** (Recorded in CDCl_3_).

No.	2		3		4		5	
	*δ*_H,_ mult. (*J* in Hz)	*δ*_C_	*δ*_H,_ mult. (*J* in Hz)	*δ*_C_	*δ*_H,_ mult. (*J* in Hz)	*δ*_C_	*δ*_H,_ mult. (*J* in Hz)	*δ*_C_
**1**	-	141.7	-	141.7	-	143.8	-	141.4
**2**	-	131.7	-	131.7	-	133.1	-	131.7
**2^1^**	3.41, *s*	12.2	3.41, *s*	12.2	3.39, *s*	12.2	3.45, *s*	12.2
**3**	-	136.0	-	136.0	-	137.0	-	136.2
**3^1^**	8.01, *dd* (18.0, 12.0)	129.4	8.01, *dd* (18.0, 12.0)	129.4	8.01, *dd* (17.6, 11.6)	132.3	8.02, *dd* (18.0, 12.0)	129.1
**3^2^**	6.18, *dd* (11.6, 1.6)	122.7	6.18, *dd* (11.6, 1.2)	122.7	6.24, *d* (12.0)	123.8	6.17, *d* (10.8)	122.9
	6.30, *dd* (18.0, 1.2)		6.30, *dd* (18.0, 1.2)		6.35, *d* (18.0)		6.32, *d* (18.0)	
**4**	-	136.2	-	136.2	-	138.1	-	136.2
**5**	9.38, *s*	97.4	9.38, *s*	97.4	10.38, *s*	102.2	9.76, *s*	104.3
**6**	-	155.4	-	155.4	-	159.5	-	155.9
**7**	-	136.4	-	136.4	-	137.9	-	136.7
**7^1^**	3.24, *s*	11.4	3.24, *s*	11.4	11.10, *s*	187.9	3.27, *s*	11.4
**8**	-	145.2	-	145.2	-	147.3	-	145.7
**8^1^**	3.69, *q* (7.6)	19.6	3.69, *q* (7.6)	19.6	4.02, *q* (7.6)	19.6	3.74, *q* (7.2)	19.7
**8^2^**	3.69, *q* (7.6)	17.4	1.69, *t* (7.6)	17.6	1.81, *t* (7.6)	19.2	1.72, *t* (7.2)	17.7
**9**	-	150.9	-	150.9	-	151.0	-	150.2
**10**	9.49, *s*	104.2	9.50, *s*	104.3	9.67, *s*	106.8	9.51, *s*	99.8
**11**	-	138.0	-	138.0	-	138.1	-	138.9
**12**	-	130.6	-	130.6	-	127.9	-	131.6
**12^1^**	3.69, *s*	12.0	3.67, *s*	12.2	3.64, *s*	12.6	3.90, *s*	12.6
**13**	-	128.5	-	128.5	-	128.8	-	111.5
**13^1^**	-	196.4	-	196.4	-	192.1	-	102.1
**13^2^**	-	ND	5.13, *d* (20.0)	48.2	-	89.0	-	100.6
			5.24, *d* (20.0)					
**13^3^**	-	-	-	-	-	173.7	-	171.3
**13^4^**	-	-	-	-	3.72, *s*	53.7	3.78, *s*	54.3
**14**	-	149.2	-	149.2	-	151.1	-	161.2
**15**	-	106.2	-	106.2	-	107.6	-	134.6
**16**	-	160.5	-	160.4	-	165.4	-	166.5
**17**	4.30, *m*	51.8	4.29, *d* (8.4, 2.4)	51.7	4.14, *m*	52.2	4.08, *d* (8.8)	53.9
**17^1^**	2.54, *m*	30.0	2.56, *m*; 2.69, *m*	30.0	2.59, *m*; 2.96, *m*	31.1	2.16, *m*	32.2
**17^2^**	2.28, *m*	31.3	2.25–2.34, *m*	31.1	2.48, *m*	31.8	2.44, *m*	31.4
**17^3^**	-	173.2	-	173.6	-	174.4	-	173.4
**17^4^**	4.08, dd (6.8, 2.4)	60.3	3.61, *s*	51.8	4.13, *m*	60.6	3.95, *m*	60.6
**17^5^**	1.16, *t* (6.8)	14.3	-	-	1.17, *t* (6.8)	14.3	1.07, *t* (7.6)	14.2
**18**	4.50, *dd* (7.2, 1.6)	50.1	4.48, *dd* (7.2, 1.6)	50.1	4.49, *dd* (7.6, 7.2)	50.5	4.45, *d* (6.8)	50.3
**18^1^**	1.81, *d* (7.6)	23.3	1.81, *d* (7.6)	23.3	1.62, *d* (7.2)	22.8	1.60, *d* (7.2)	22.4
**19**	-	171.6	-	171.5	-	172.6	-	171.1
**20**	8.55, *s*	93.2	8.55, *s*	93.2	8.61, *s*	94.0	8.71, *s*	94.0
**1**″	-	178.3						
**2**″	2.35, *t* (7.6)	33.9						
**3**″	1.64, *m*	24.8						
**4**″	1.24–1.30, *m*	29.8						
**5**″	1.24–1.30, *m*	29.8						
**6**″	1.24–1.30, *m*	29.5						
**7**″	1.24–1.30, *m*	22.8						
**8**″	0.88, *t* (6.4)	14.3						

ND: not determined. The assignments were determined by DEPT, COSY, HSQC, and HMBC experiments.

**Table 3:** ADME predictions of bioactive compounds computed by SwissADME ^a^.

Compound	6	7	8	Gabapentin
GI abs	High	High	High	High
BBB per	No	No	Yes	Yes
P-gp substrate	No	No	No	No
CYP1A2 inhibitor	No	No	No	No
CYP2C19 inhibitor	No	No	Yes	No
CYP2C9 inhibitor	Yes	Yes	Yes	No
CYP2D6 inhibitor	No	No	Yes	No
CYP3A4 inhibitor	Yes	Yes	Yes	No

^a^ Log *K*_p_: log of skin permeability; GI abs: gastro-intestinal absorption; BBB per: blood–brain barrier permeability; P-gp: P-glycoprotein; CYP: cytochrome-P.

**Table 4:** Drug properties of isolated compounds analyzed with SwissADME ^a^.

Compound	6	7	8
MW (g mol^−1^)	358.47	372.50	314.46
Log *P*	3.45	4.38	2.17
nHBD	0	0	0
nHBA	4	4	2
TPSA	77.76	66.76	40.46
MR	106.81	111.13	99.85
Lipinski violation	0	0	0
Log *S*	−5.93	−6.15	−5.54
nRotB	7	8	6

^a^ MW: molecular weight; log *P*: log of octanol/water partition coefficient; nHBA: number of hydrogen bond acceptor(s); nHBD: number of hydrogen bond donor(s); MR: molar refractivity; nRotB: number of rotatable bonds; log *S*: log of solubility; TPSA: total polar surface area.

## Data Availability

All data are presented in the article.

## Supplementary Materials

The following supporting information can be downloaded at https://www.mdpi.com/article/10.3390/ph18040521/s1, Figure S1–S57: 1D NMR, 2D NMR, and HRESIMS spectra of isolated compounds (**1**–**11**).
