# Supplementary material for: New Cannabinoids and Chlorin-Type Metabolites from the Flowers of Cannabis sativa L.: A Study on Their Neuroblastoma Activity
Source: Pharmaceuticals (Basel). 2025 Apr 3;18(4):521. doi: 10.3390/ph18040521 (PMC12030031; doi:10.3390/ph18040521)
Supplement: Supplementary file 1 [file pharmaceuticals-18-00521-s001.zip › pharmaceuticals-3542000-supplementary.pdf]

Supplementary Materials

New Cannabinoids and Chlorin-Type Metabolites From the Flowers of *Cannabis sativa* L.: A study on Their Neuroblastoma Activity

Tuan-Quoc Nguyen [1], Jae-Yong Cheon[1], Hyo-Shin Park[1], Sun-Hyeong Choi[1], Hong-Da Yun[1,2], Young-Mi Lee[1], Chul-Min Kim[3], Jong-Ki Hong[4], Seo Jeong[4], Man-Soo Cho[5], Jang-Hoon Kim[6], Eun-Sol Lee[7], Jung-Won Seo[7], and Hyun-Ju Jung[1,*]

[1]Department of Oriental Pharmacy, College of Pharmacy and Wonkwang-Oriental Medicines Reseach Institute, Wonkwang University, Sinyong-Dong, Iksan 570-749, Republic of Korea; quoctuan301281@gmail.com (T.N.Q); cjy21750@naver.com (J.-Y.C.); phs4846@naver.com (H.-S.P.); subear780@gmail.com (S.-H.C.); ymlee@wku.ac.kr (Y.-M.L.);

[2]Korean Ministry of Food and Drug Safety, Osongaengmyeong 2-ro 187, Osong-eup, Heungdeok-gu, Cheongju-si, Chungcheongbuk-do, Republic of Korea; hong13@naver.com (H.D.Y.);

[3]Department of Horticulture Industry, Wonkwang University, Iksan 54538, Republic of Korea; chumin21@wku.ac.kr (C.M.K.);

[4]College of Pharmacy, Kyung Hee university, Seoul 02447, Republic of Korea; jhong@khu.ac.kr (J.H.)

[5]Department of Smart Experience Design, Kookmin University 77, Jeongneung-ro, Seongbuk-gu, Seoul 02707, Republic of Korea; chomansoo@gmail.com (M.S.C.)

[6]Department of Herbal Crop Reasearch, National Institute of Horticultural and Herbal Science, RDA, Enmseong 27709, Republic of Korea; jhkim53@korea.kr (J.H.K.);

[7]Institute of Pharmaceutical Research and Development, College of Pharmacy, Wonkwang University, Iksan 54538, Republic of Korea; chori0509@naver.com (E.-S.L.); jwseo@wku.ac.kr (J.S.);

**Correspondence: hyun104@wku.ac.kr; Tel.: +82-63-850-6814*

List of supplementary data

Figure S1. MS spectrum of **1** 1
Figure S2. ^{1}H NMR spectrum of **1** (Recorded in $CDCl_3$) 2
Figure S3. ^{13}C NMR spectrum of **1** (Recorded in $CDCl_3$) 3
Figure S4. DEPT spectrum of **1** (Recorded in $CDCl_3$) 4
Figure S5. COSY spectrum of **1** (Recorded in $CDCl_3$) 5
Figure S6. HSQC spectrum of **1** (Recorded in $CDCl_3$) 6
Figure S7. HMBC spectrum of **1** (Recorded in $CDCl_3$) 7
Figure S8. NOESY spectrum of **1** (Recorded in $CDCl_3$) 8
Figure S9. MS spectrum of **2** 9
Figure S10. ^{1}H NMR spectrum of **2** (Recorded in $CDCl_3$) 10
Figure S11. ^{13}C NMR spectrum of **2** (Recorded in $CDCl_3$) 11
Figure S12. DEPT spectrum of **2** (Recorded in $CDCl_3$) 12
Figure S13. HMQC spectrum of **2** (Recorded in $CDCl_3$) 13
Figure S14. COSY spectrum of **2** (Recorded in $CDCl_3$) 14
Figure S15. HMBC spectrum of **2** (Recorded in $CDCl_3$) 15
Figure S16. MS spectrum of **3** 16
Figure S17. ^{1}H NMR spectrum of **3** (Recorded in $CDCl_3$) 17
Figure S18. ^{13}C NMR spectrum of **3** (Recorded in $CDCl_3$) 18
Figure S19. DEPT spectrum of **3** (Recorded in $CDCl_3$) 19
Figure S20. HMQC spectrum of 3 (Recorded in $CDCl_3$) 20
Figure S21. COSY spectrum of **3** (Recorded in $CDCl_3$) 21
Figure S22. HMBC spectrum of **3** (Recorded in $CDCl_3$) 22
Figure S23. MS spectrum of **4** 23
Figure S24. ^{1}H NMR spectrum of **4** (Recorded in $CDCl_3$) 24
Figure S25. ^{13}C NMR spectrum of **4** (Recorded in $CDCl_3$) 25
Figure S26. DEPT spectrum of **4** (Recorded in $CDCl_3$) 26
Figure S27. HMQC spectrum of **4** (Recorded in $CDCl_3$) 27
Figure S28. COSY spectrum of **4** (Recorded in $CDCl_3$) 28
Figure S29. HMBC spectrum of **4** (Recorded in $CDCl_3$) 29

Figure S30. MS spectrum of **5** 30
Figure S31. ^{1}H NMR spectrum of **5** (Recorded in $CDCl_3$) 31
Figure S32. ^{13}C NMR spectrum of **5** (Recorded in $CDCl_3$) 32
Figure S33. DEPT spectrum of **5** (Recorded in $CDCl_3$)........ 33
Figure S34. COSY spectrum of **5** (Recorded in $CDCl_3$) 34
Figure S35. HMQC spectrum of **5** (Recorded in $CDCl_3$)........ 35
Figure S36. HMBC spectrum of **5** (Recorded in $CDCl_3$)........ 36
Figure S37. MS spectrum of **6** 37
Figure S38. ^{1}H NMR spectrum of **6** (Recorded in $CDCl_3$) 38
Figure S39. ^{13}C NMR spectrum of **6** (Recorded in $CDCl_3$) 39
Figure S40. DEPT spectrum of **6** (Recorded in $CDCl_3$)........ 40
Figure S41. MS spectrum of **7** 41
Figure S42. ^{1}H NMR spectrum of **7** (Recorded in $CDCl_3$) 42
Figure S43. ^{13}C NMR spectrum of **7** (Recorded in $CDCl_3$) 43
Figure S44. MS spectrum of **8** 44
Figure S45. ^{1}H NMR spectrum of **8** (Recorded in CD_3OD) 45
Figure S46. ^{13}C NMR spectrum of **8** (Recorded in CD_3OD) 46
Figure S47. MS spectrum of **9** 47
Figure S48. ^{1}H NMR spectrum of **9** (Recorded in $CDCl_3$) 48
Figure S49. ^{13}C NMR spectrum of **9** (Recorded in $CDCl_3$) 49
Figure S50. MS spectrum of **10** 50
Figure S51. ^{1}H NMR spectrum of **10** (Recorded in $CDCl_3$) 51
Figure S52. ^{13}C NMR spectrum of **10** (Recorded in $CDCl_3$) 52
Figure S53. DEPT spectrum of **10** (Recorded in $CDCl_3$)........ 53
Figure S54. MS spectrum of **11**........ 54
Figure S55. ^{1}H NMR spectrum of **11** (Recorded in $CDCl_3$)........ 55
Figure S56. ^{13}C NMR spectrum of **11** (Recorded in $CDCl_3$) 56
Figure S57. DEPT spectrum of **11** (Recorded in $CDCl_3$)........ 57

1. Spectroscopic data for compound 1

Elemental composition	Theoretical m/z	Observed m/z	Error (ppm / mDa)
$C_{22}H_{28}O_4$	357.2060	357.2069	-2.42 / -0.86
	379.1880	379.1896	-4.27 / -1.62

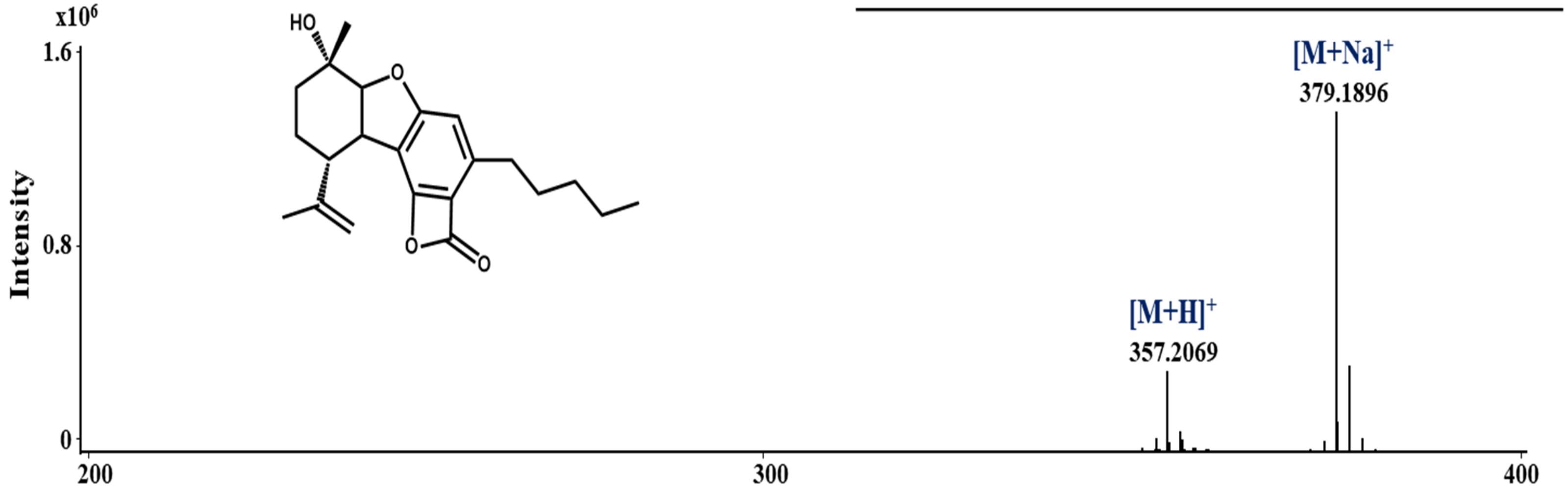

Figure S1. MS spectrum of **1**

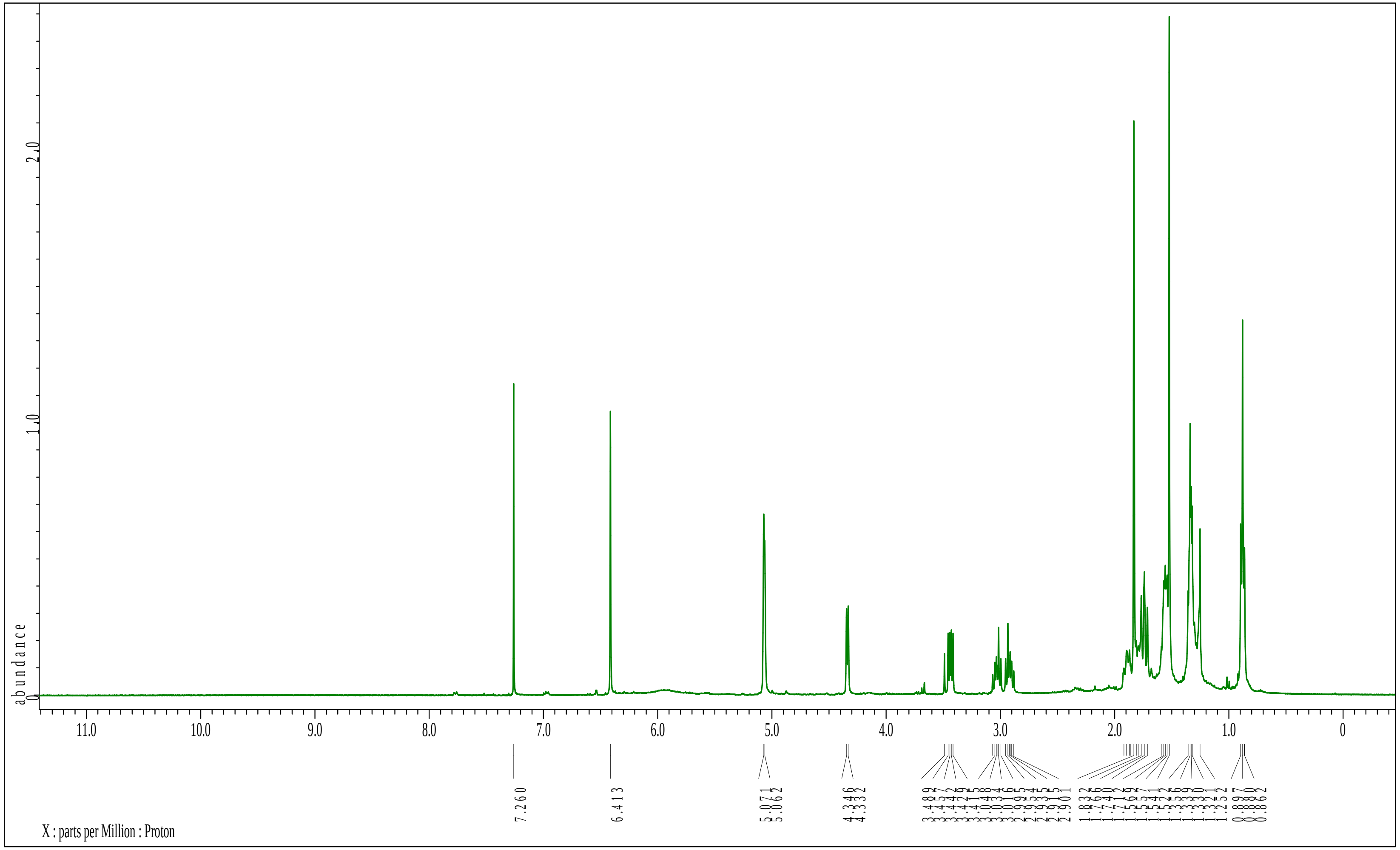

Figure S2. ^{1}H NMR spectrum of **1** (Recorded in $CDCl_3$)

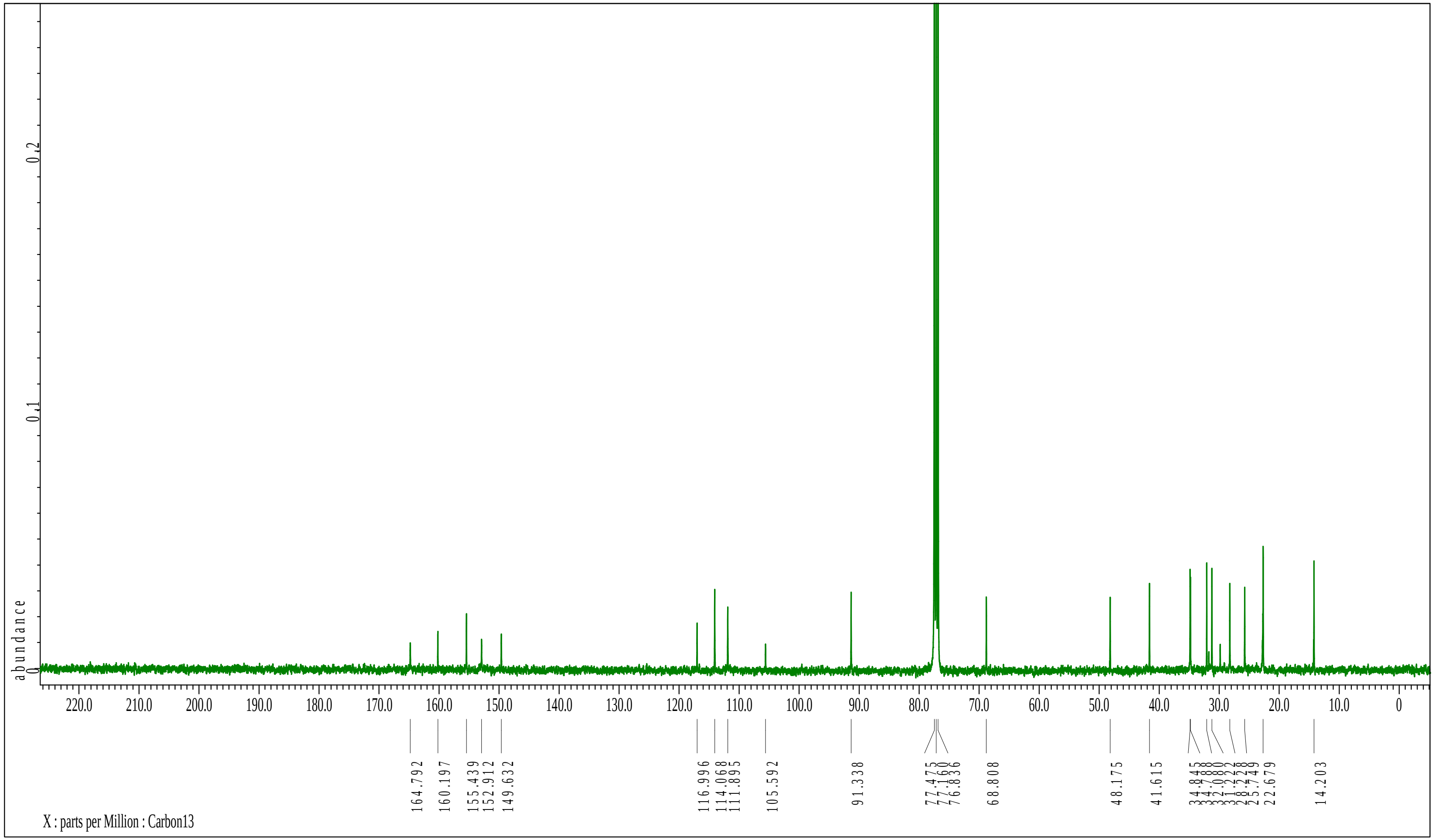

Figure S3. ^{13}C NMR spectrum of **1** (Recorded in $CDCl_3$)

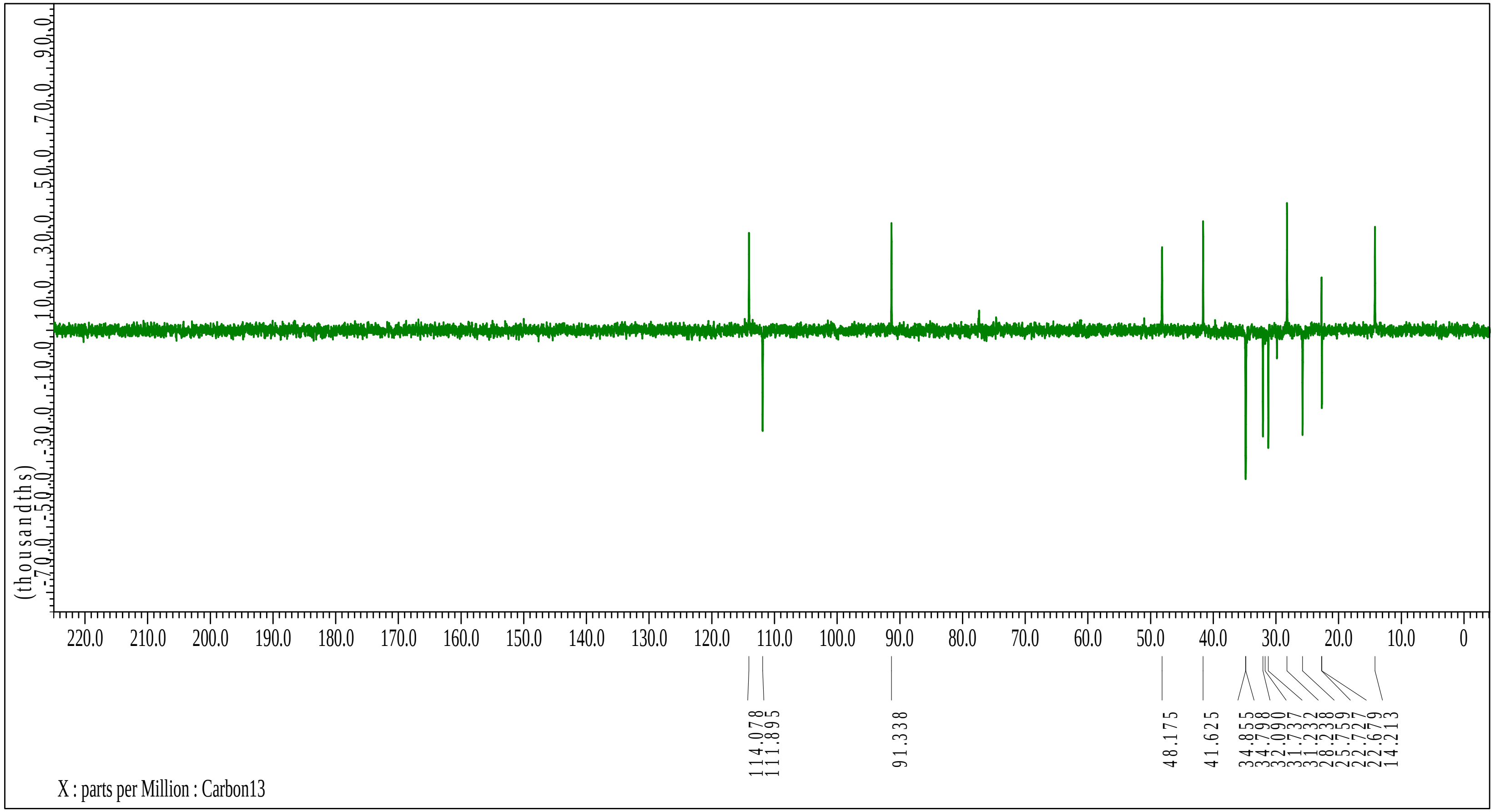

Figure S4. DEPT spectrum of **1** (Recorded in $CDCl_3$)

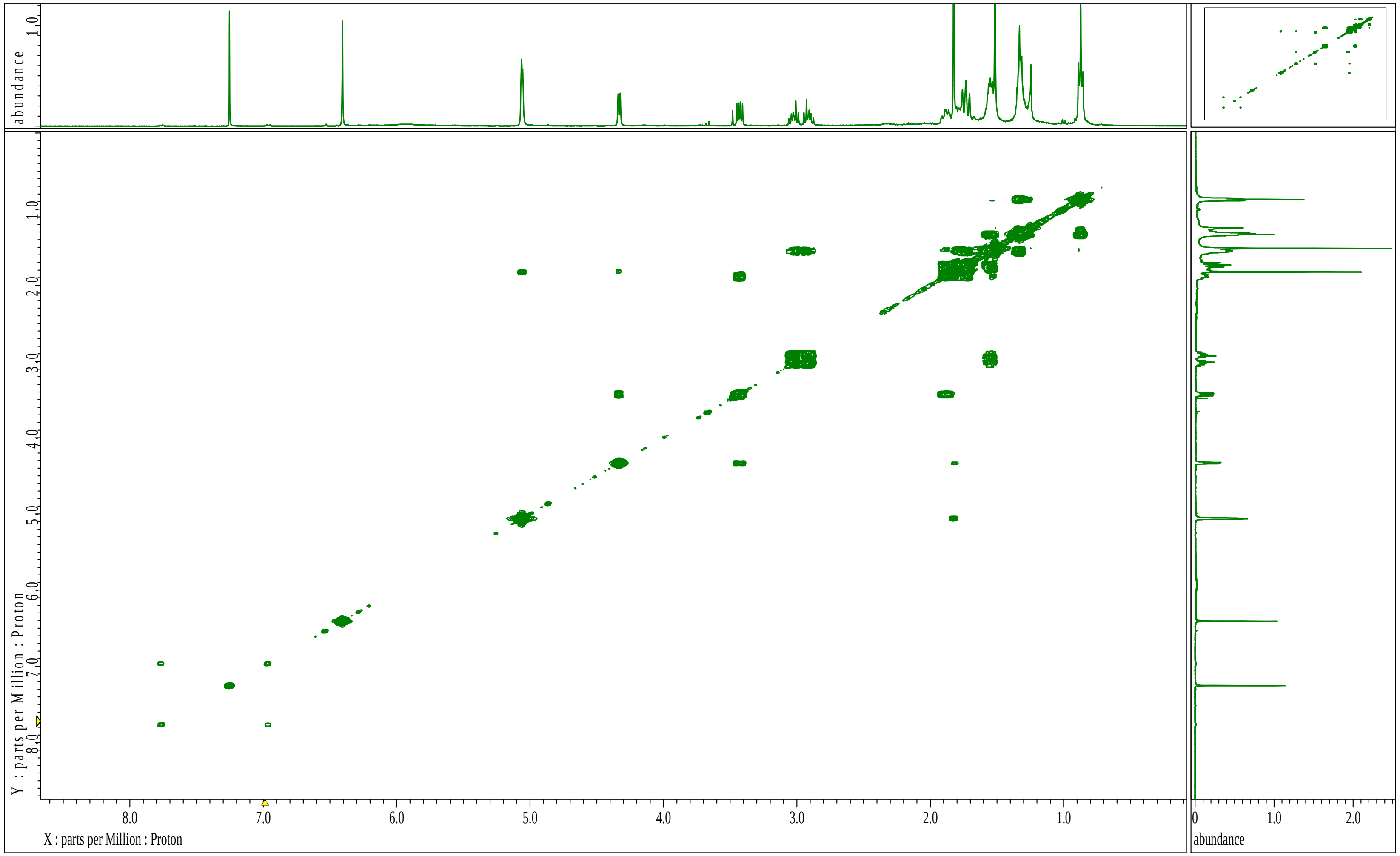

Figure S5. COSY spectrum of **1** (Recorded in $CDCl_3$)

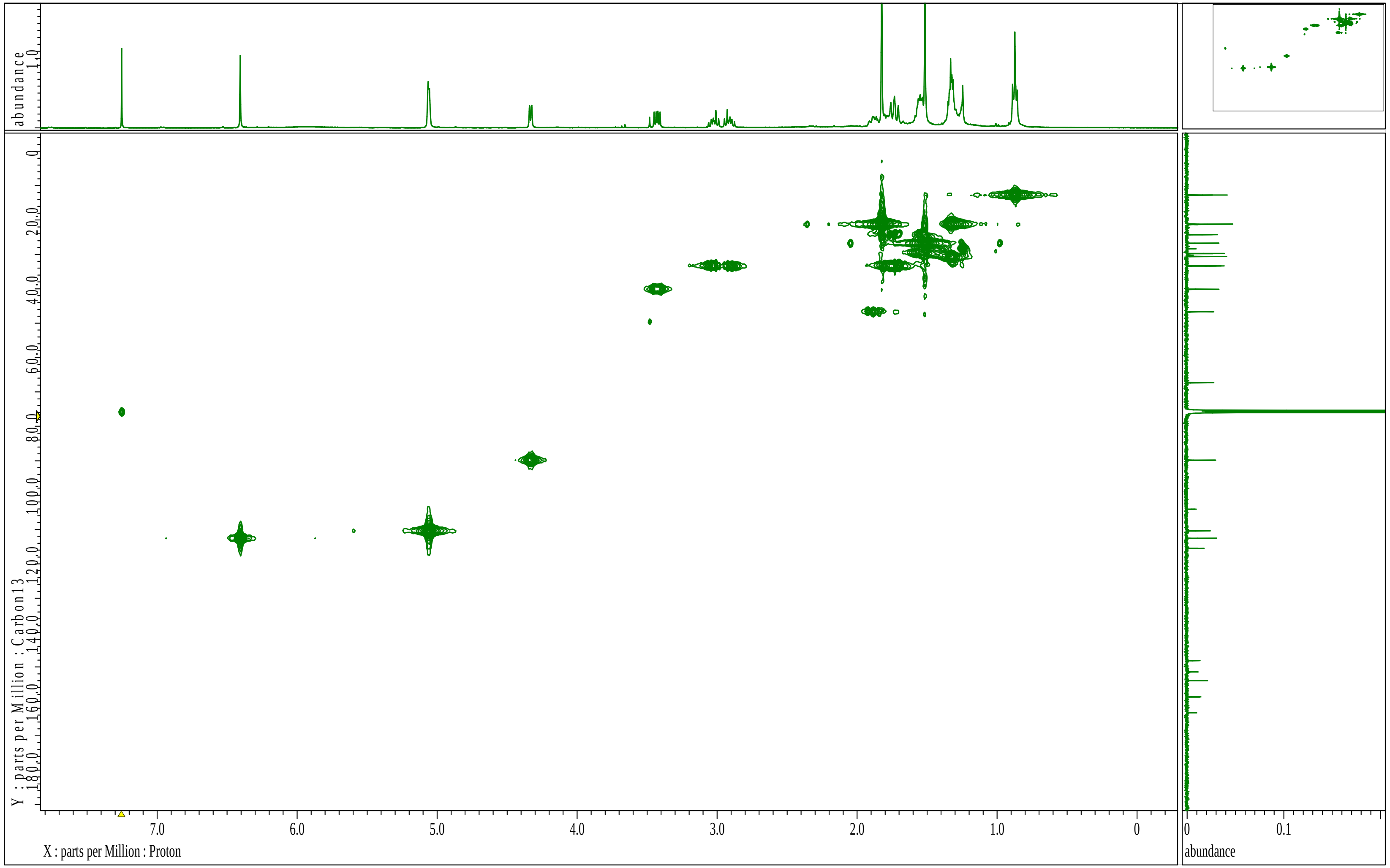

Figure S6. HSQC spectrum of **1** (Recorded in $CDCl_3$)

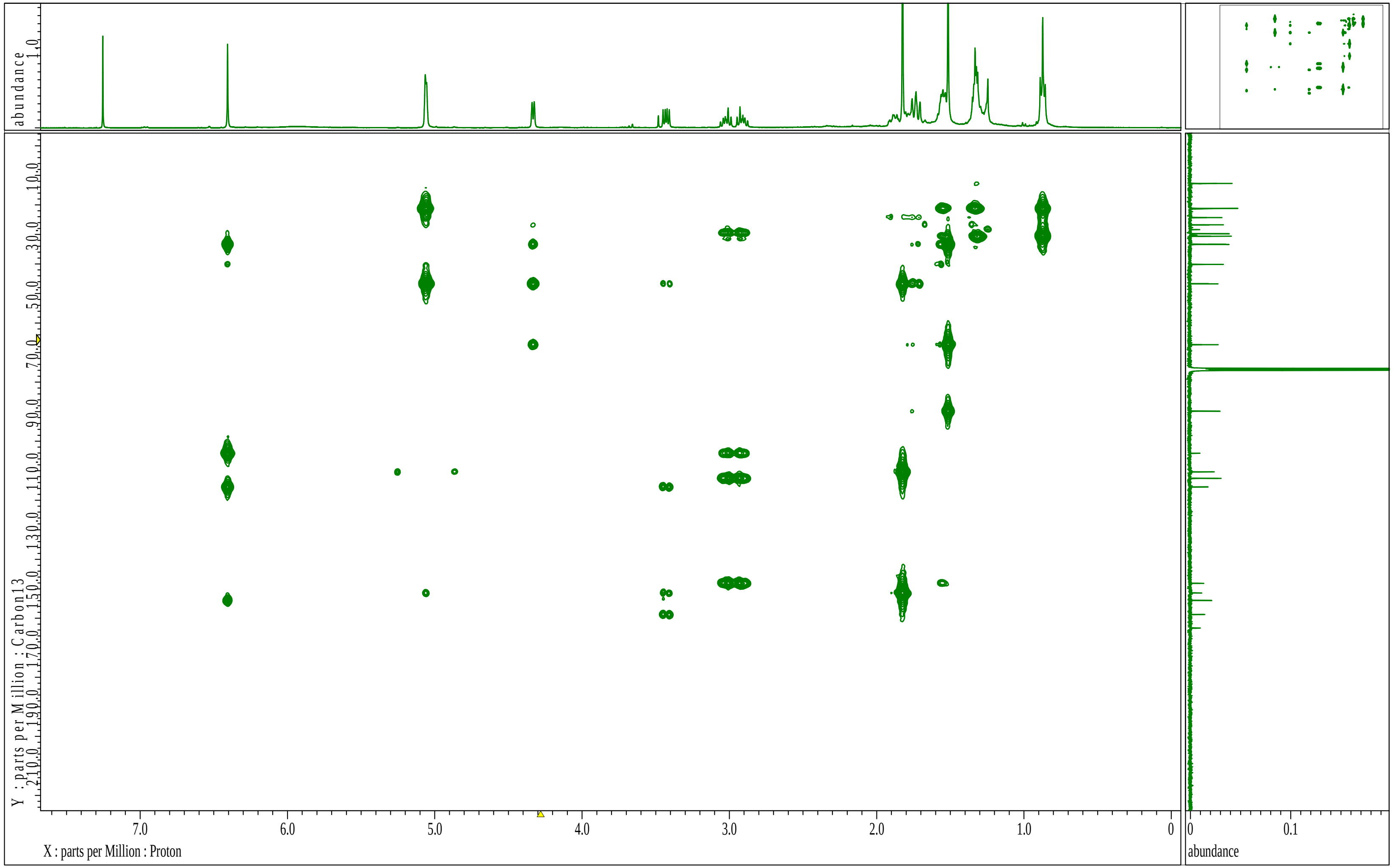

Figure S7. HMBC spectrum of **1** (Recorded in $CDCl_3$)

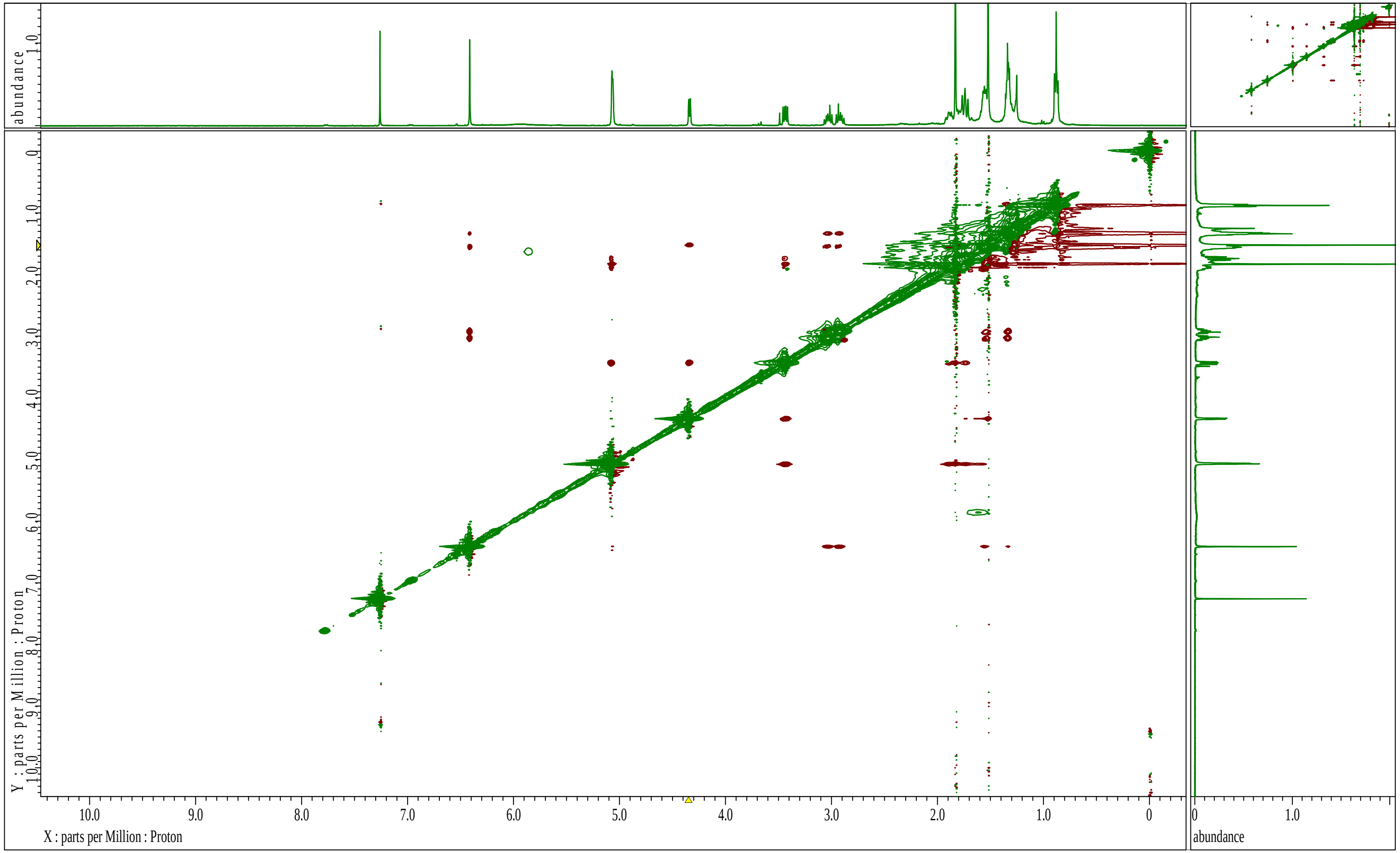

Figure S8. NOESY spectrum of **1** (Recorded in $CDCl_3$)

2. Spectroscopic data for compound 2

Elemental composition	Theoretical m/z	Observed m/z	Error (ppm / mDa)
$C_{43}H_{52}N_4O_6$	721.3960	721.3988	- 3.93 / - 2.84

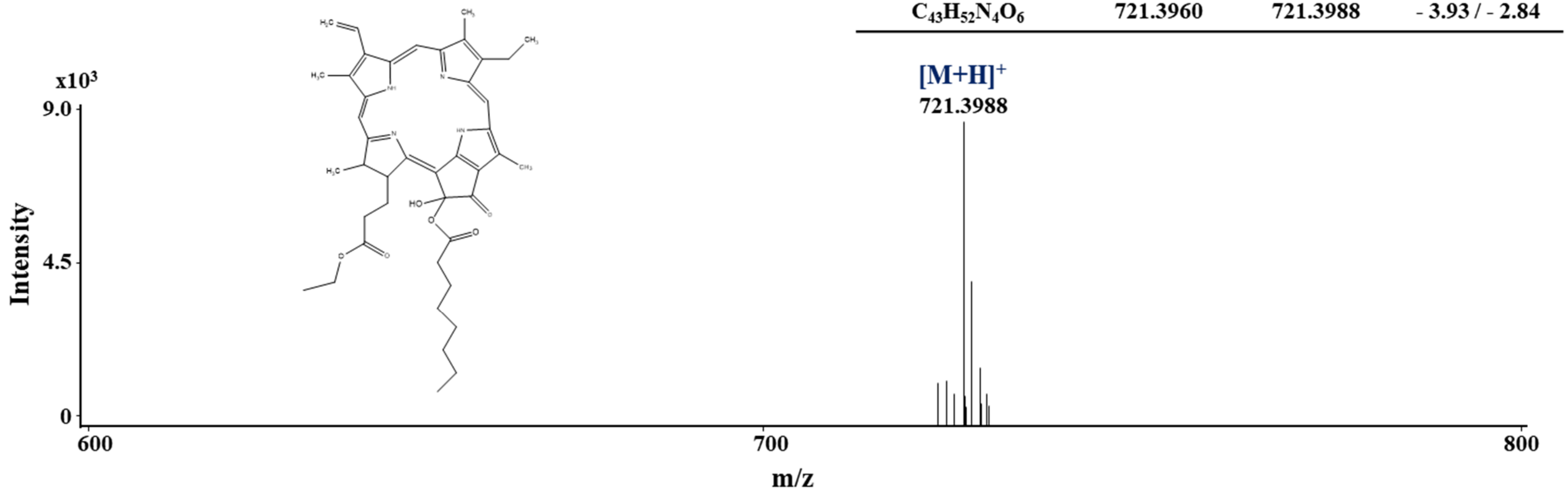

Figure S9. MS spectrum of **2**

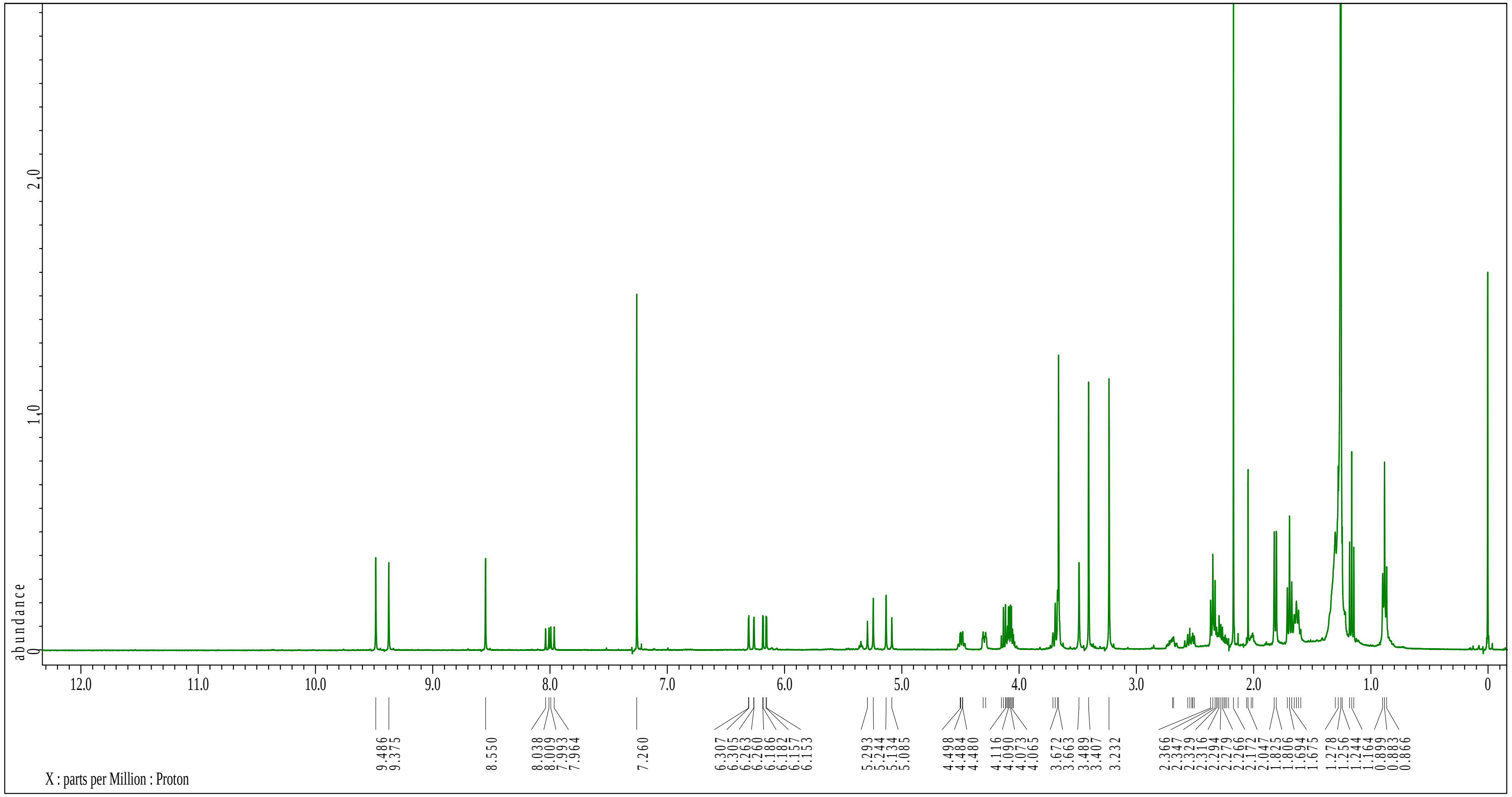

Figure S10. ^{1}H NMR spectrum of **2** (Recorded in $CDCl_3$)

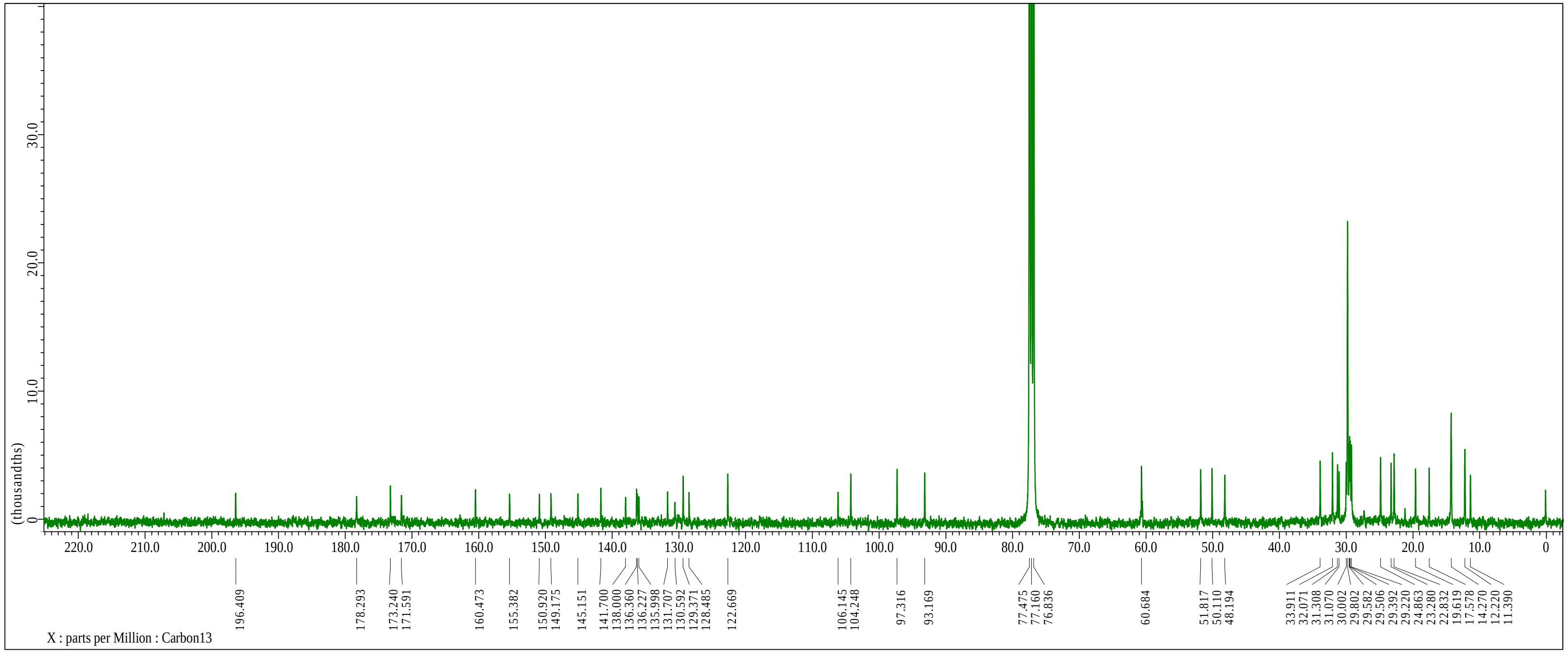

Figure S11. ^{13}C NMR spectrum of **2** (Recorded in $CDCl_3$)

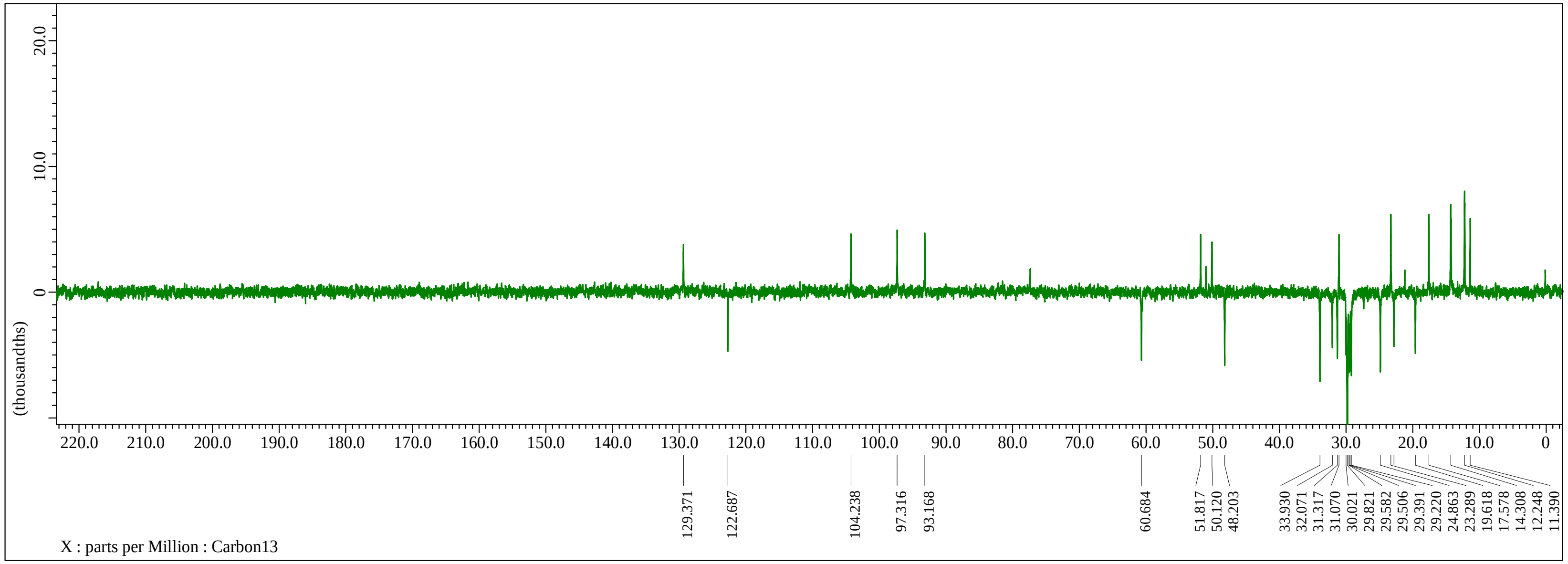

Figure S12. DEPT spectrum of **2** (Recorded in $CDCl_3$)

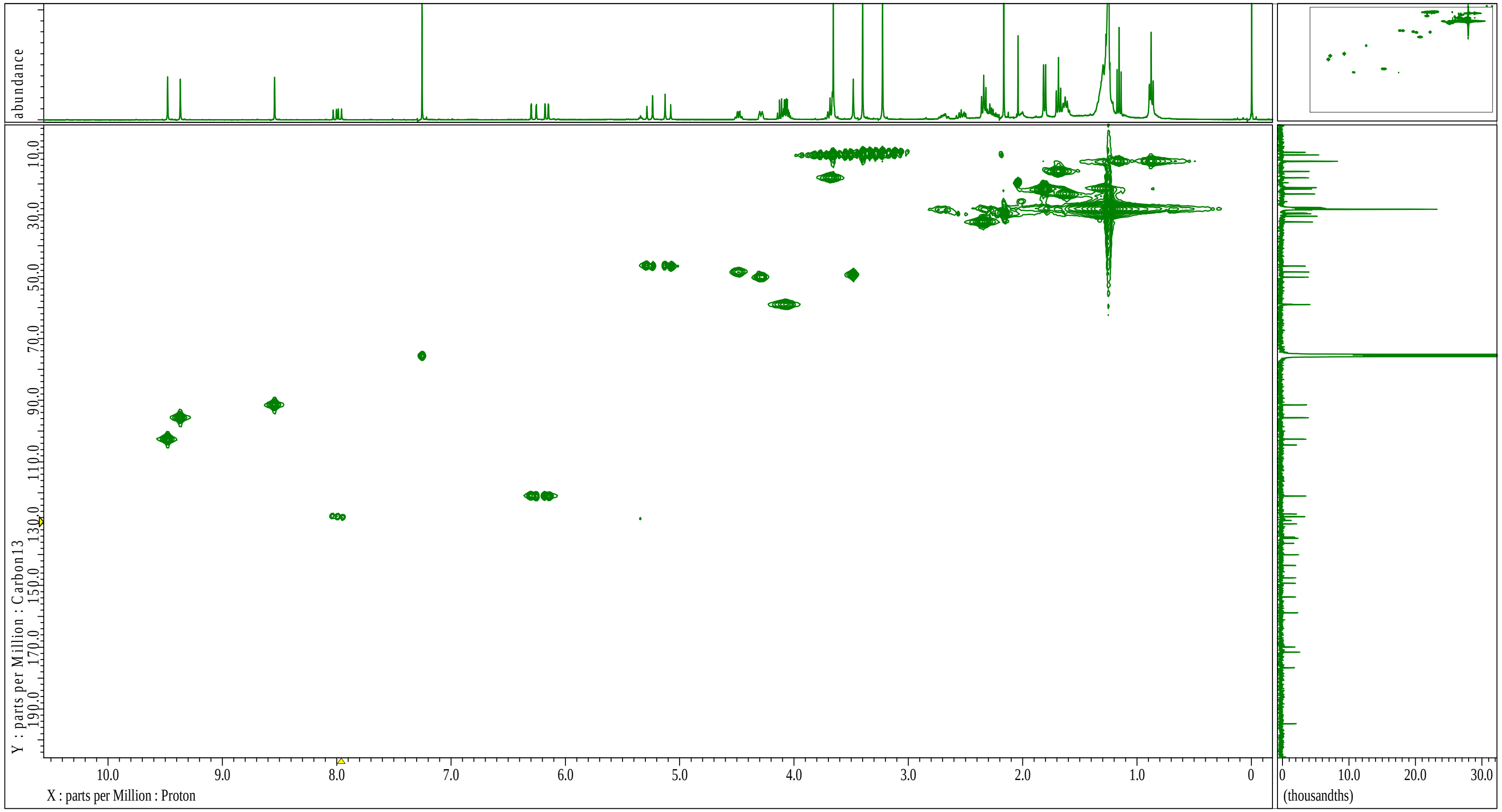

Figure S13. HMQC spectrum of **2** (Recorded in $CDCl_3$)

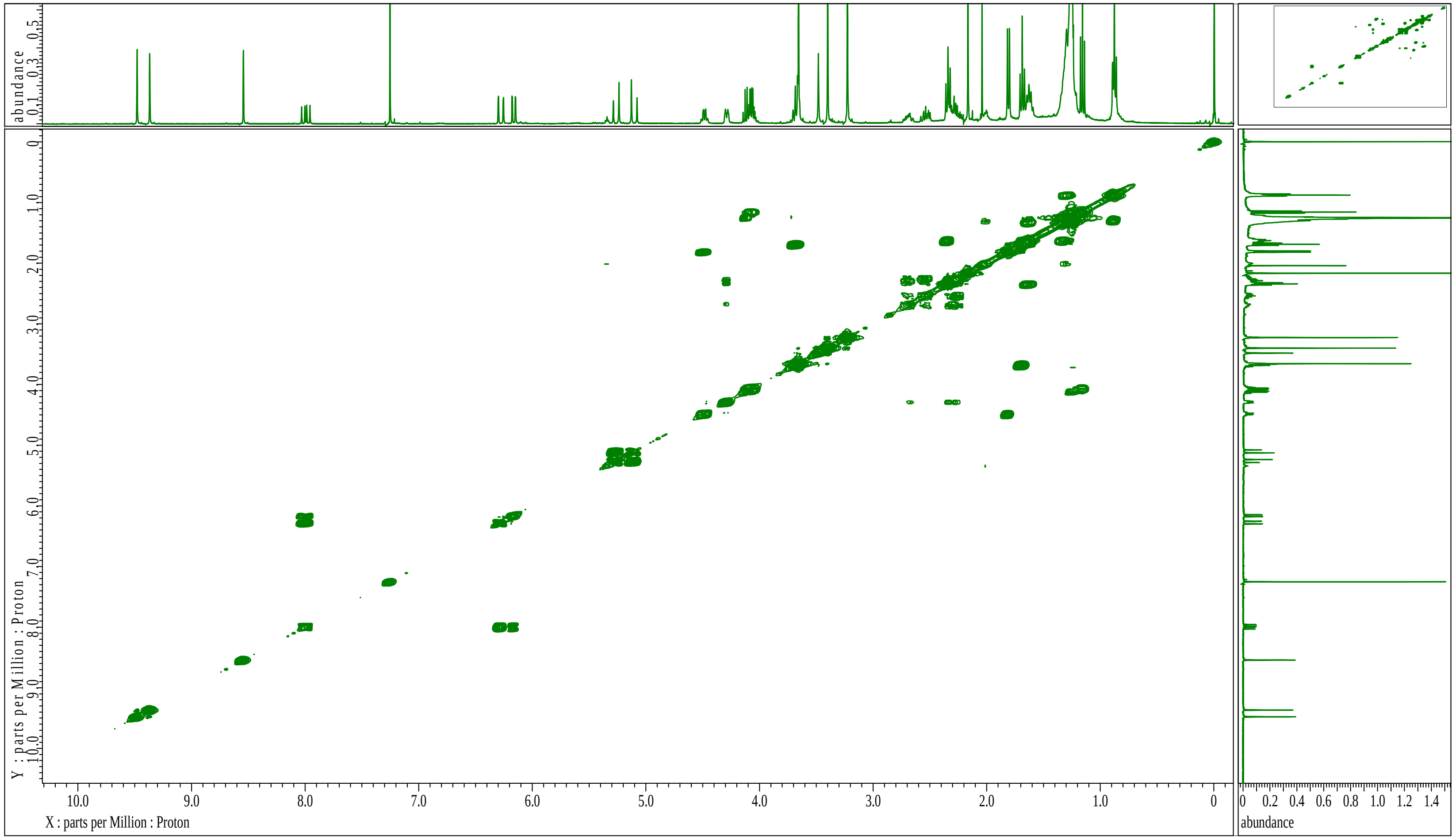

Figure S14. COSY spectrum of **2** (Recorded in $CDCl_3$)

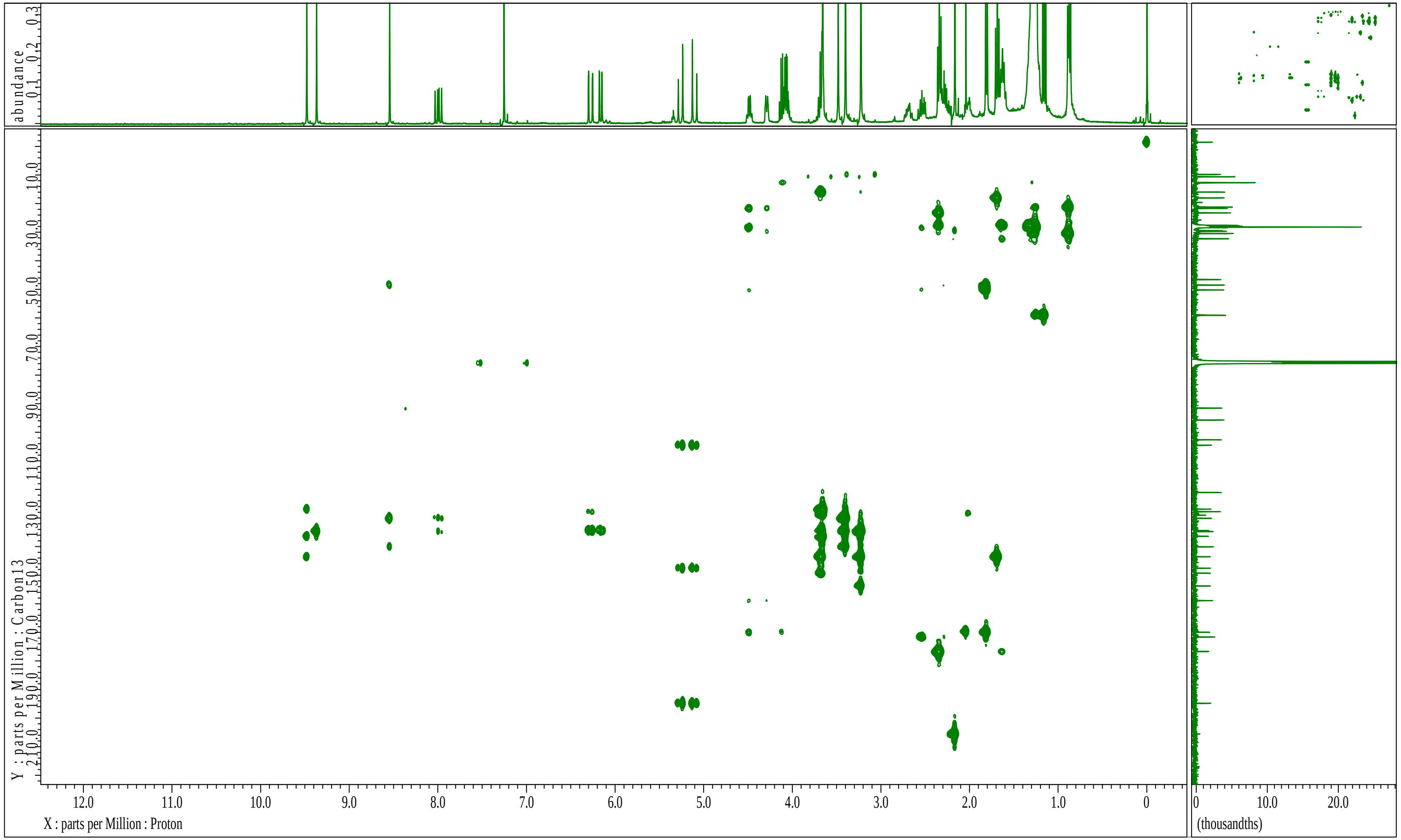

Figure S15. HMBC spectrum of **2** (Recorded in $CDCl_3$)

3. Spectroscopic data for compound 3

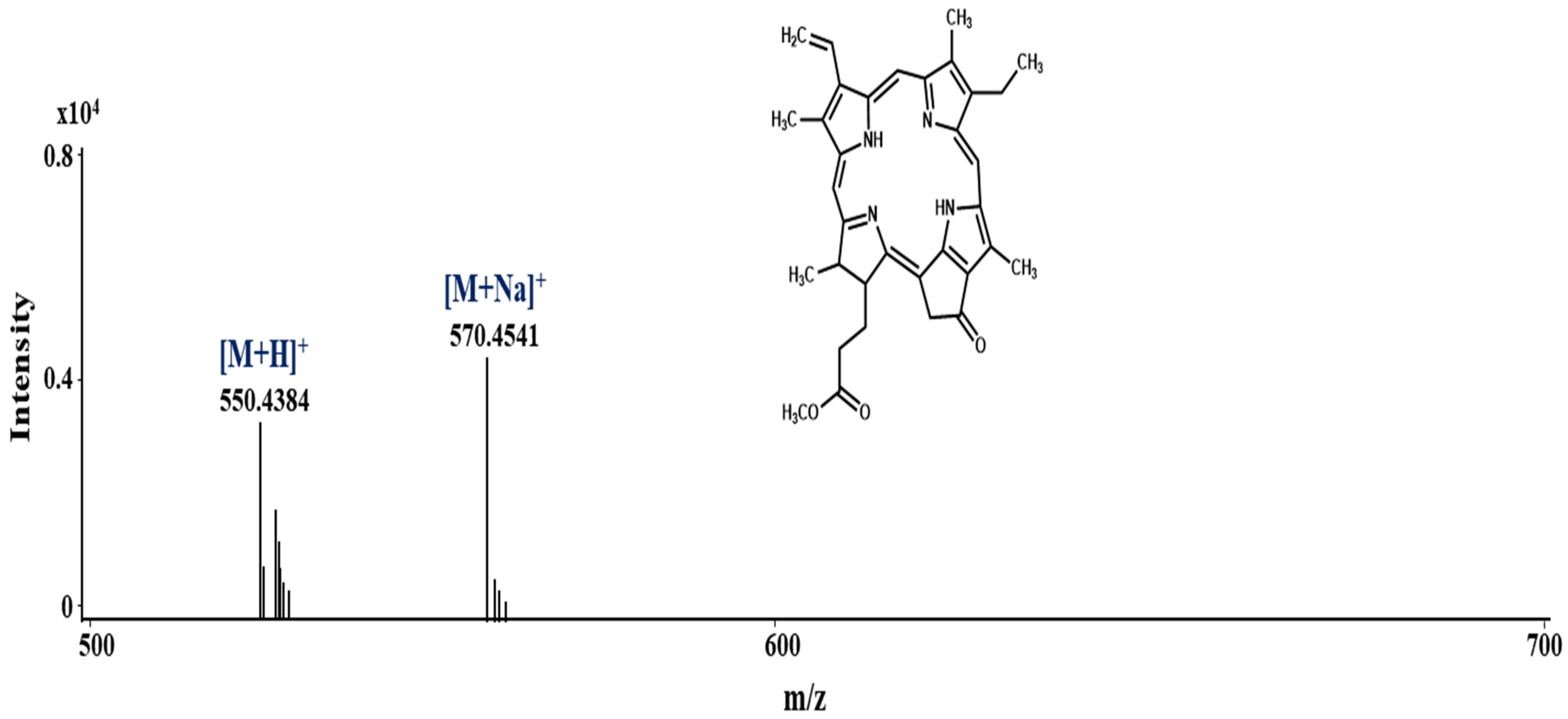

Figure S16. MS spectrum of **3**

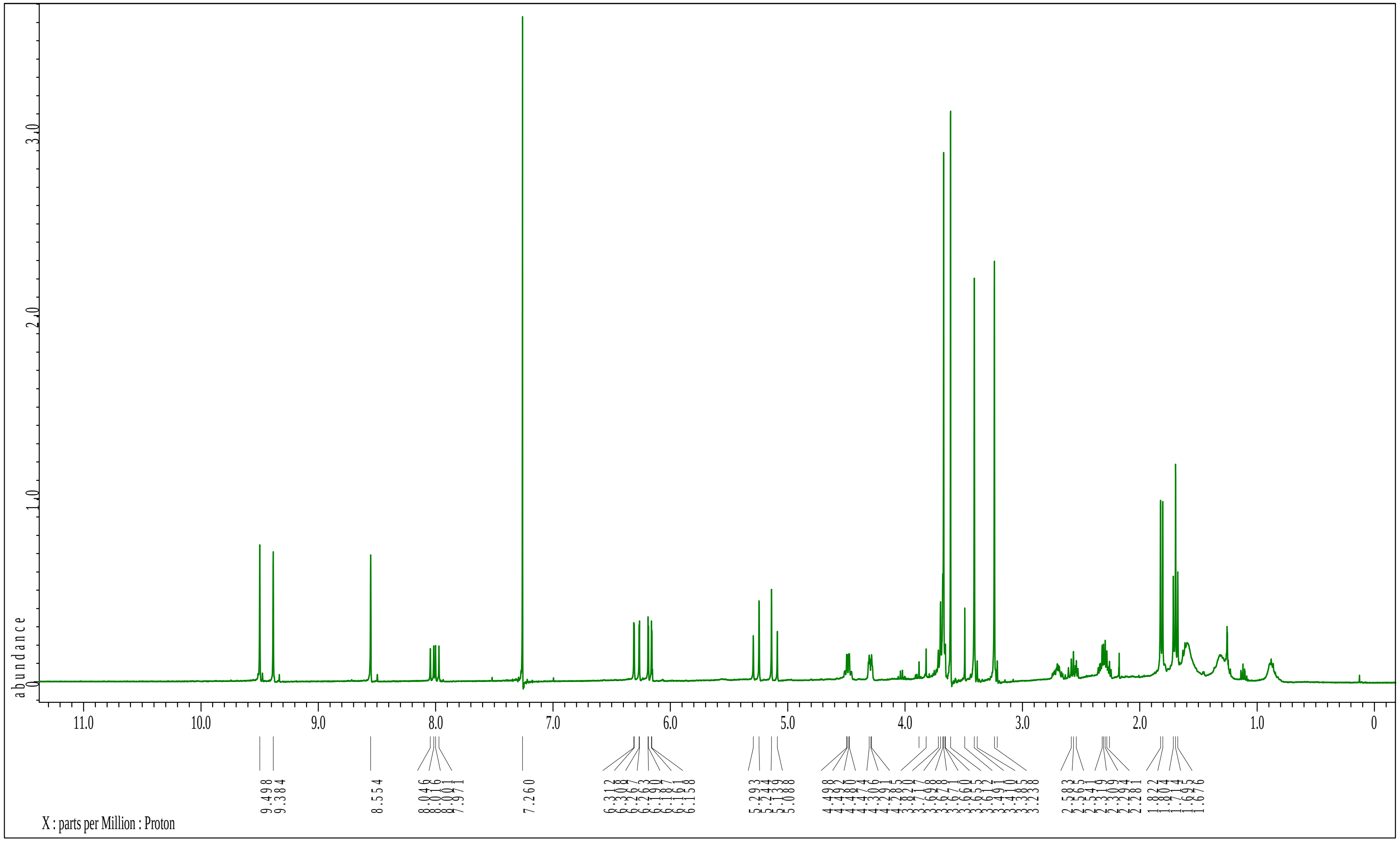

Figure S17. ^{1}H NMR spectrum of **3** (Recorded in $CDCl_3$)

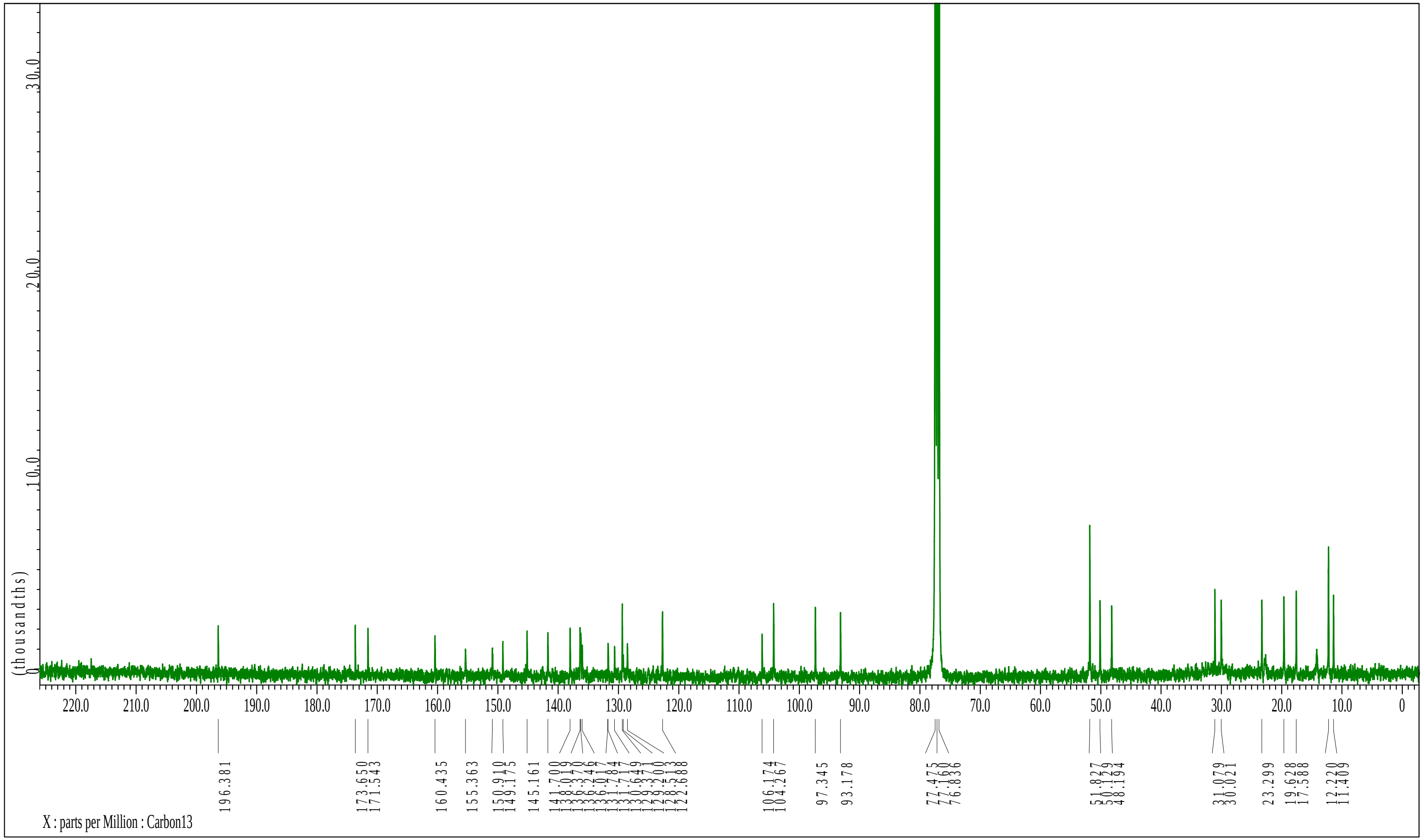

Figure S18. ^{13}C NMR spectrum of **3** (Recorded in $CDCl_3$)

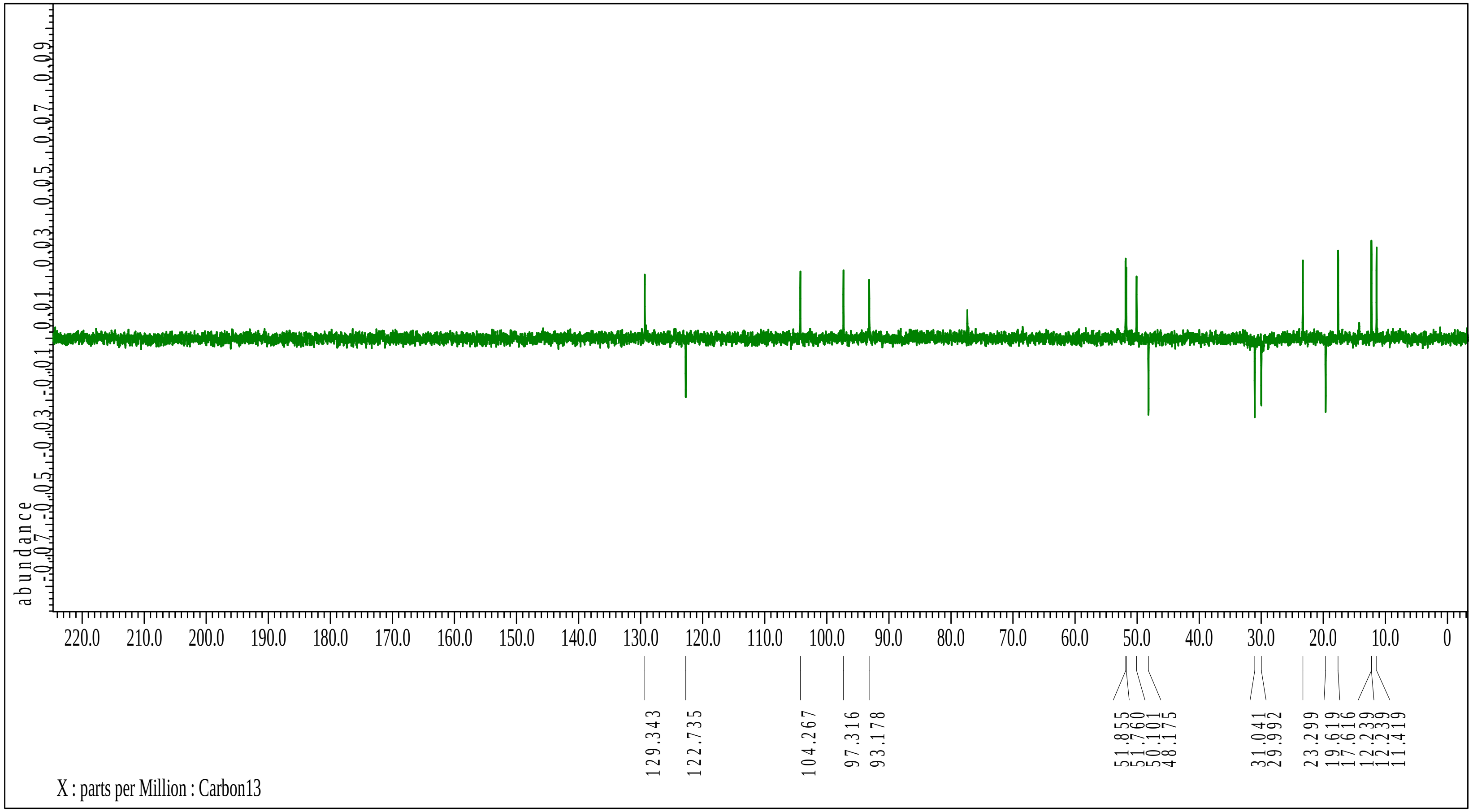

Figure S19. DEPT spectrum of **3** (Recorded in $CDCl_3$)

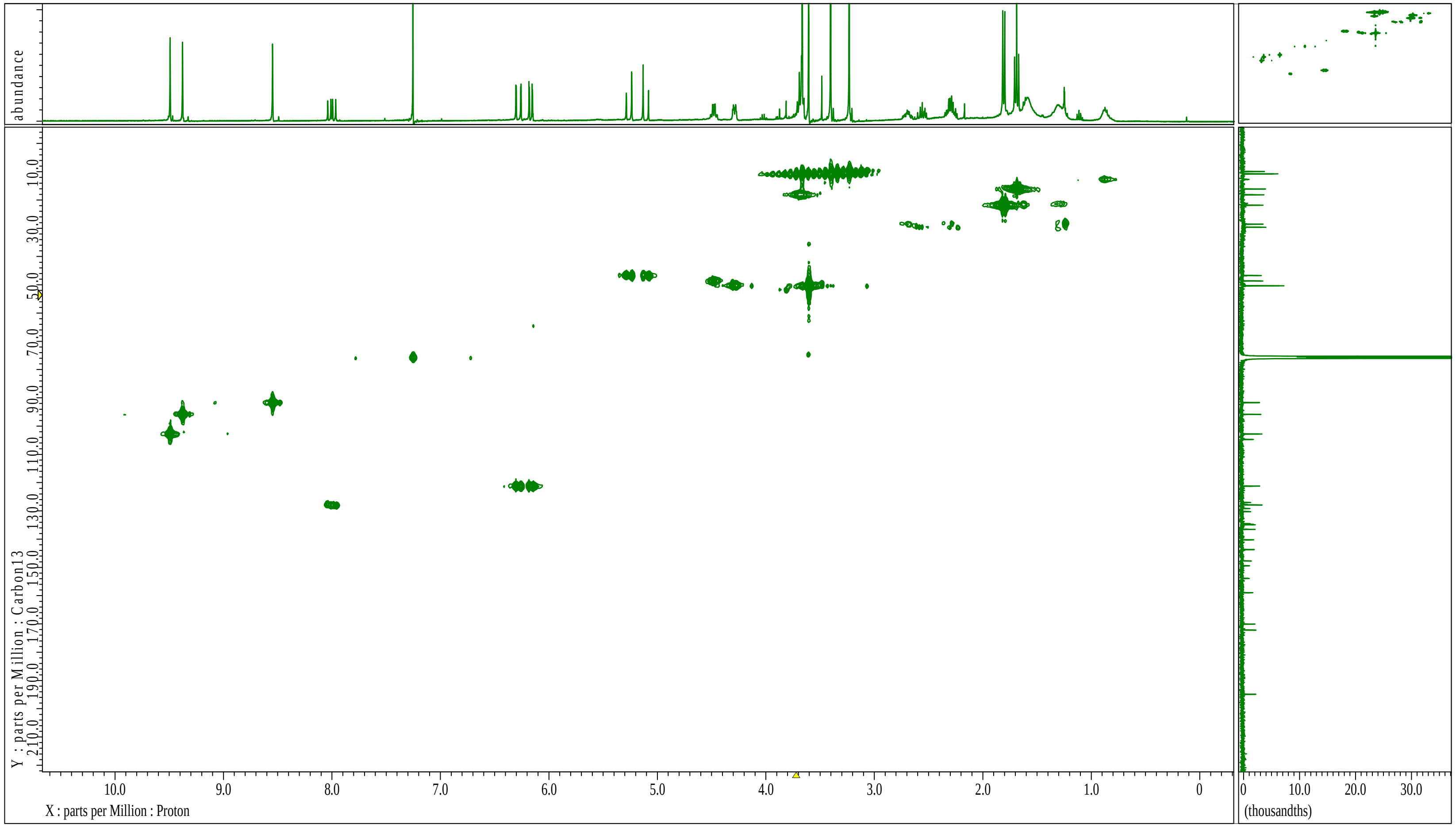

Figure S20. HMQC spectrum of 3 (Recorded in $CDCl_3$)

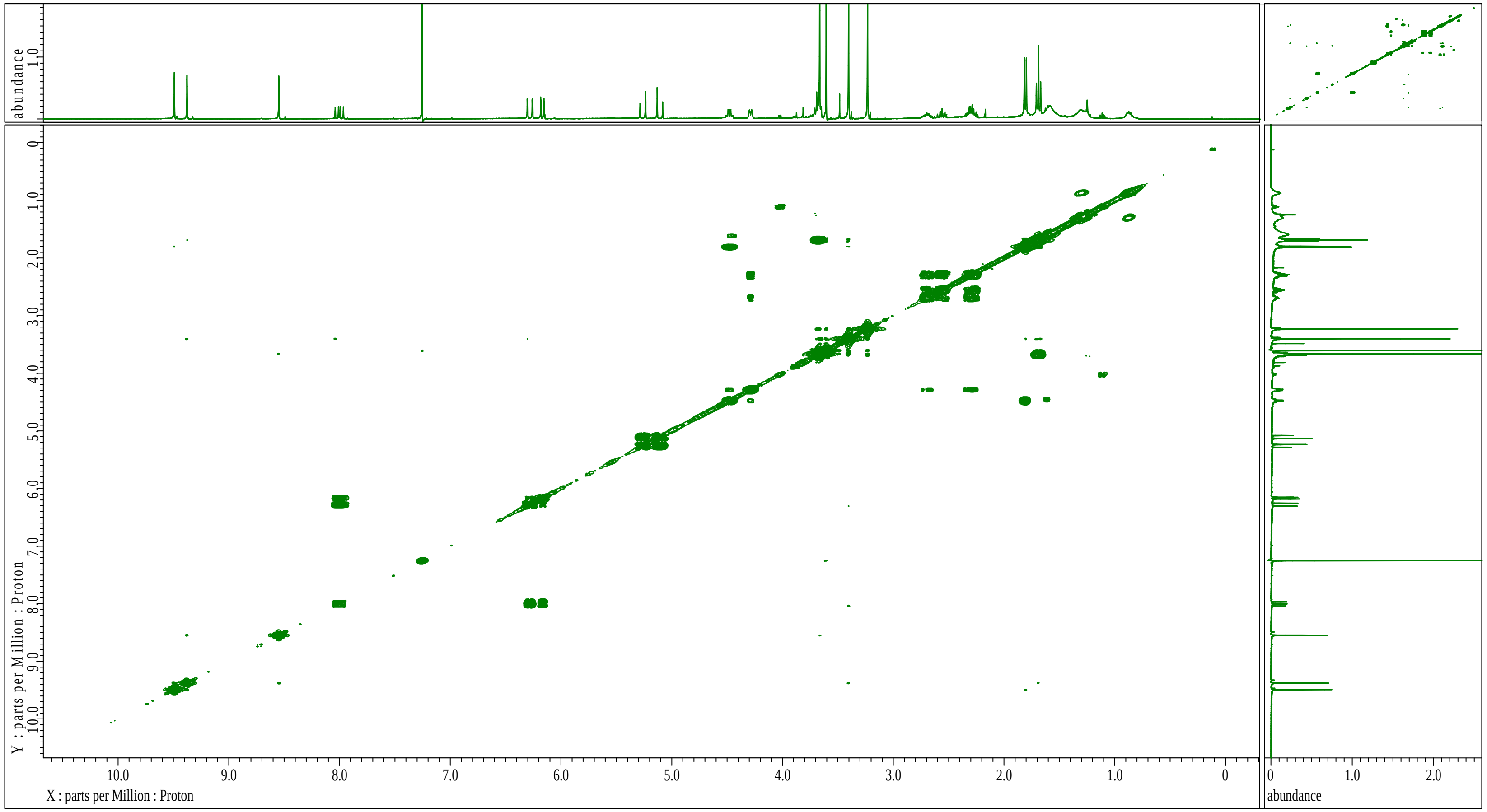

Figure S21. COSY spectrum of **3** (Recorded in $CDCl_3$)

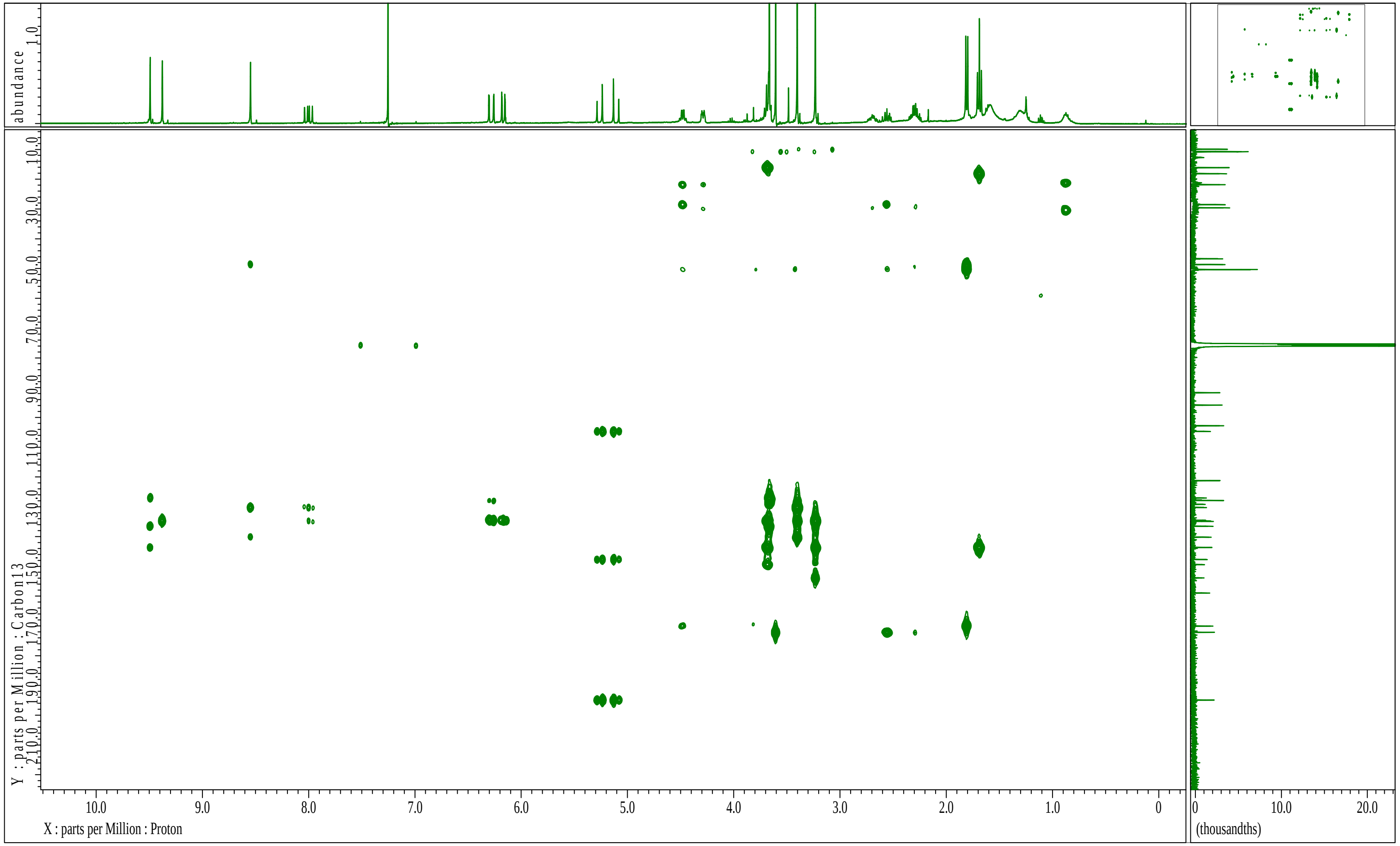

Figure S22. HMBC spectrum of **3** (Recorded in $CDCl_3$)

4. Spectroscopic data for compound 4

Elemental composition	Theoretical m/z	Observed m/z	Error (ppm / mDa)
$C_{37}H_{38}N_4O_7$	651.2813	651.2808	0.04 / 0.03

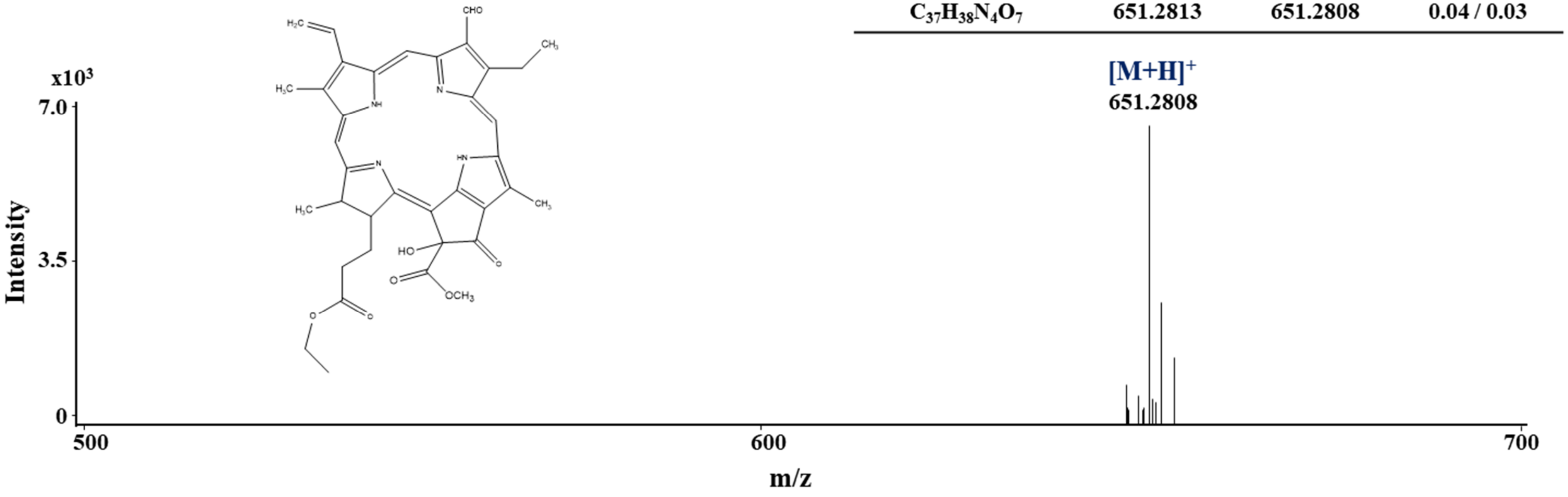

Figure S23. MS spectrum of **4**

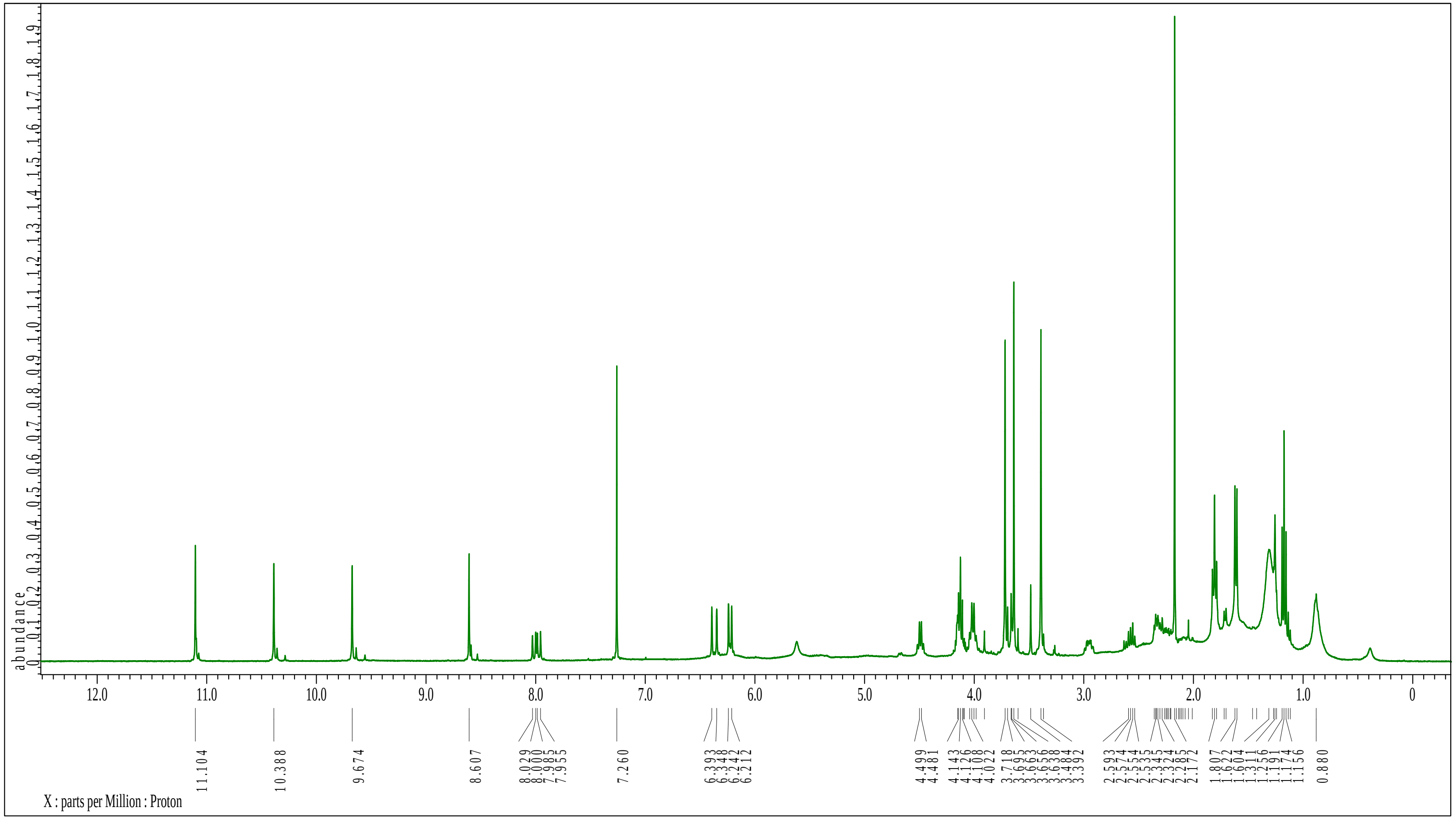

Figure S24. ^{1}H NMR spectrum of **4** (Recorded in $CDCl_3$)

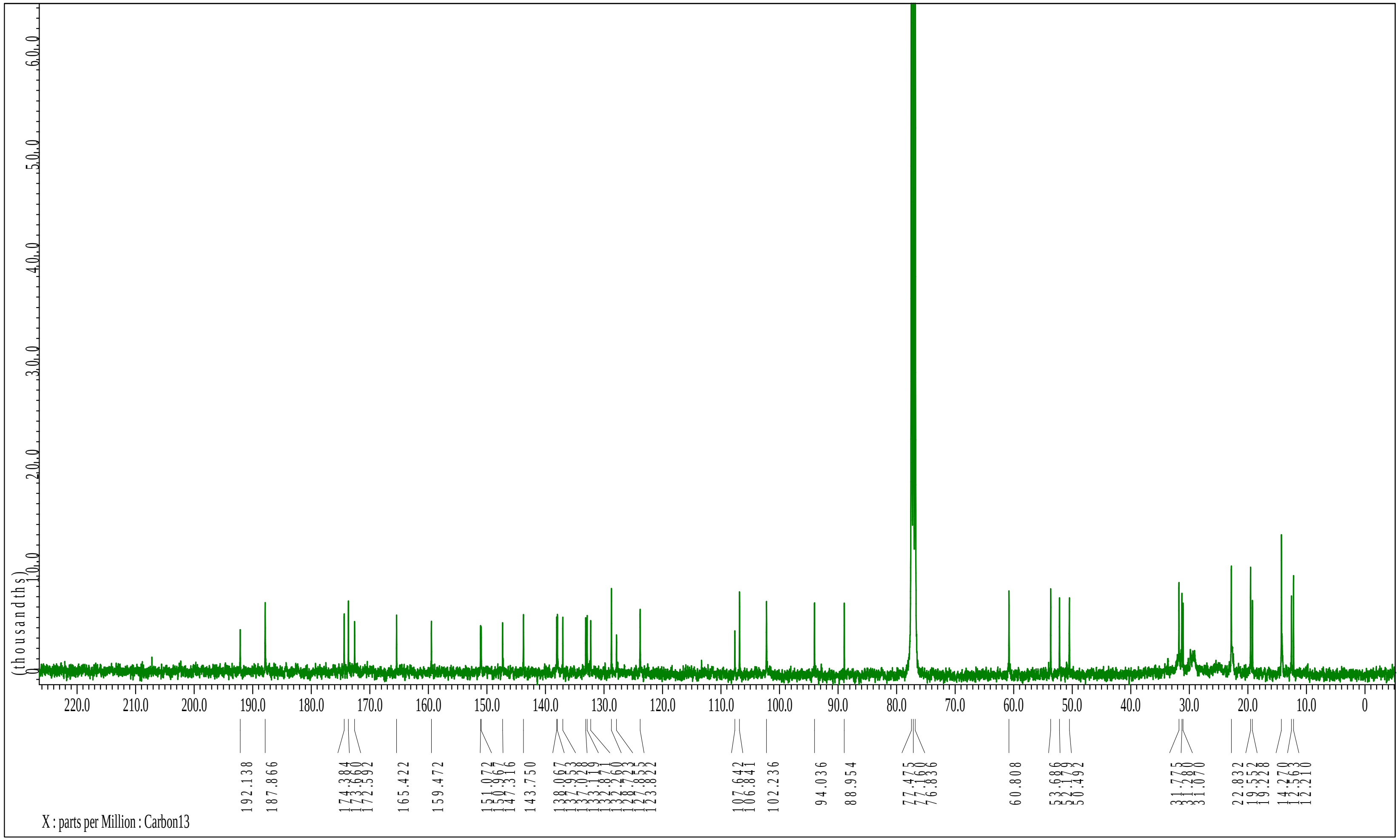

Figure S25. ^{13}C NMR spectrum of **4** (Recorded in $CDCl_3$)

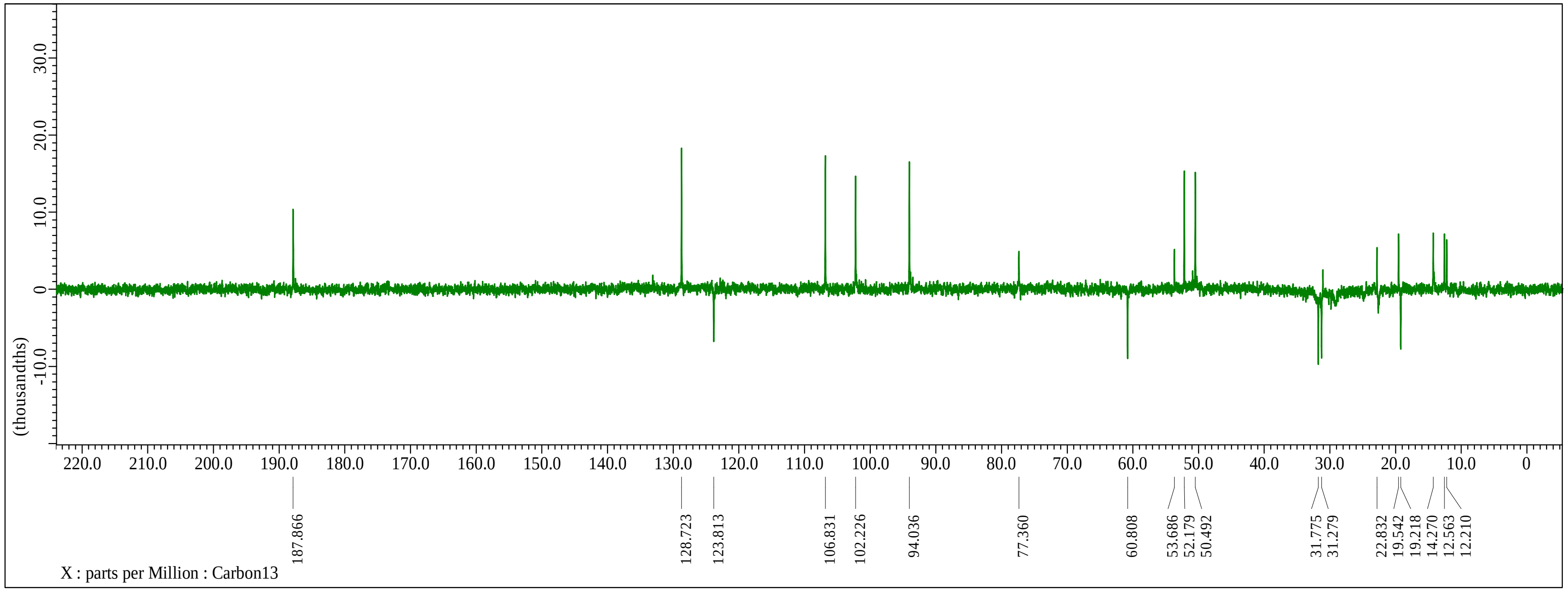

Figure S26. DEPT spectrum of **4** (Recorded in $CDCl_3$)

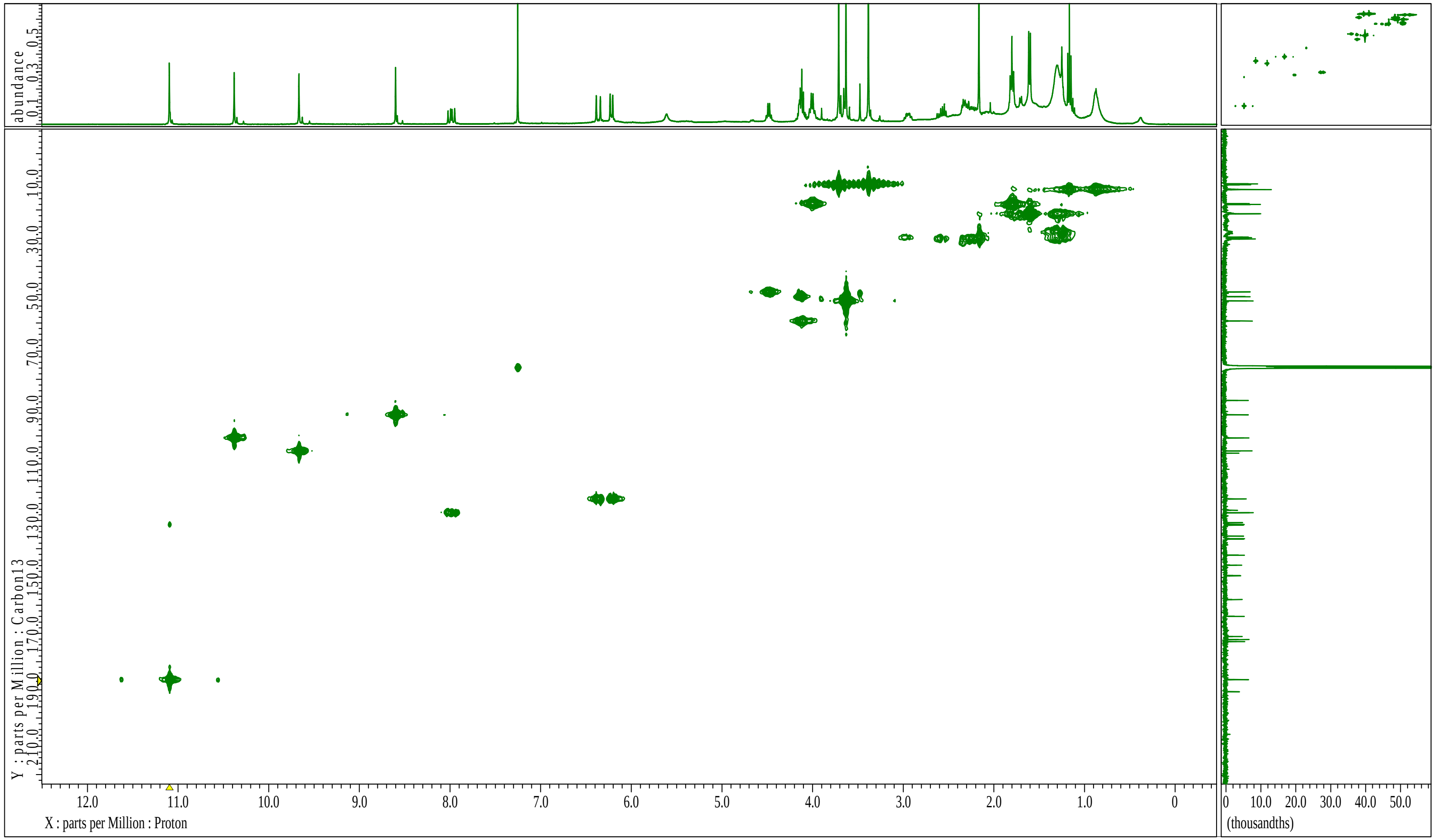

Figure S27. HMQC spectrum of **4** (Recorded in $CDCl_3$)

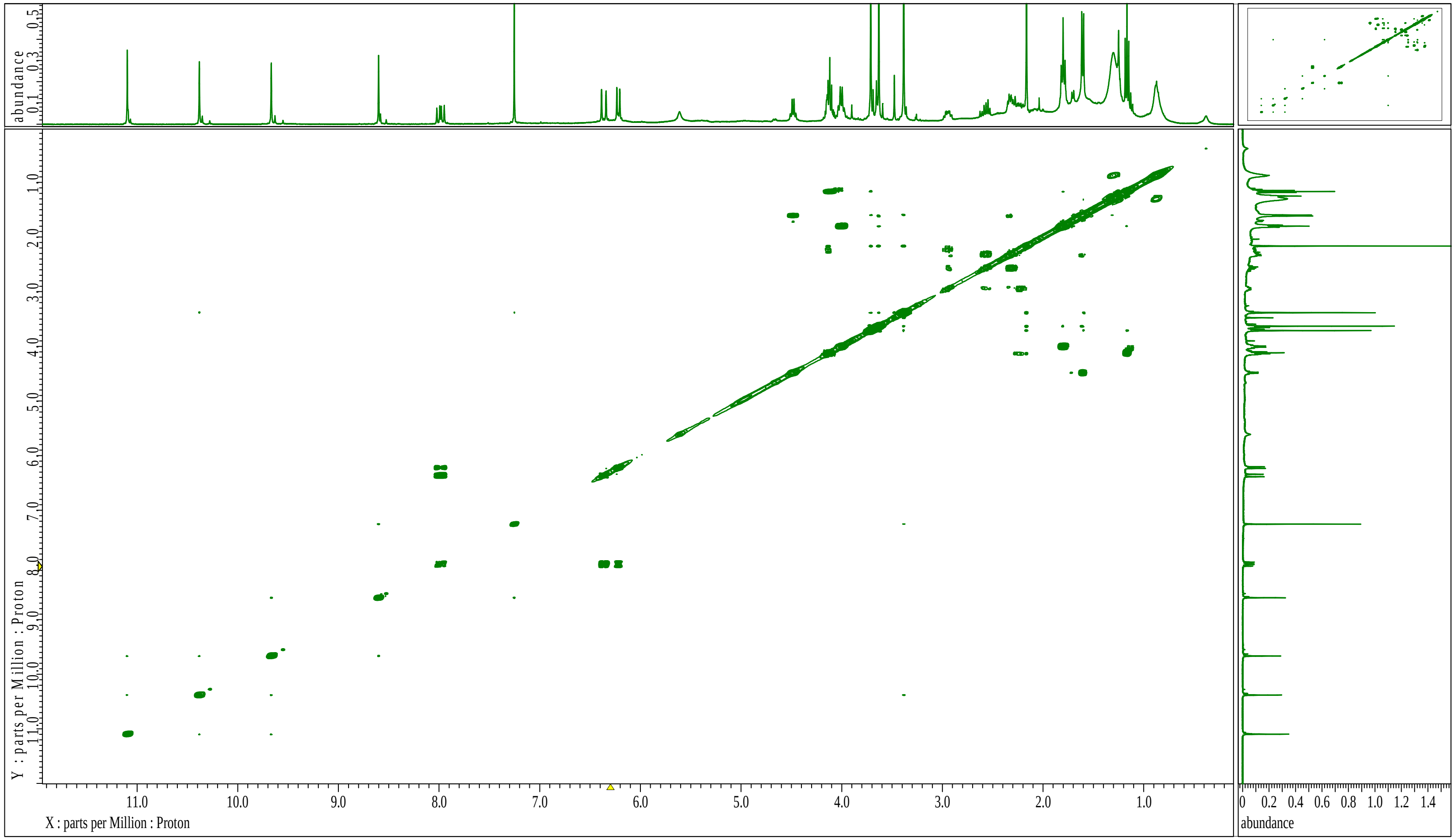

Figure S28. COSY spectrum of **4** (Recorded in $CDCl_3$)

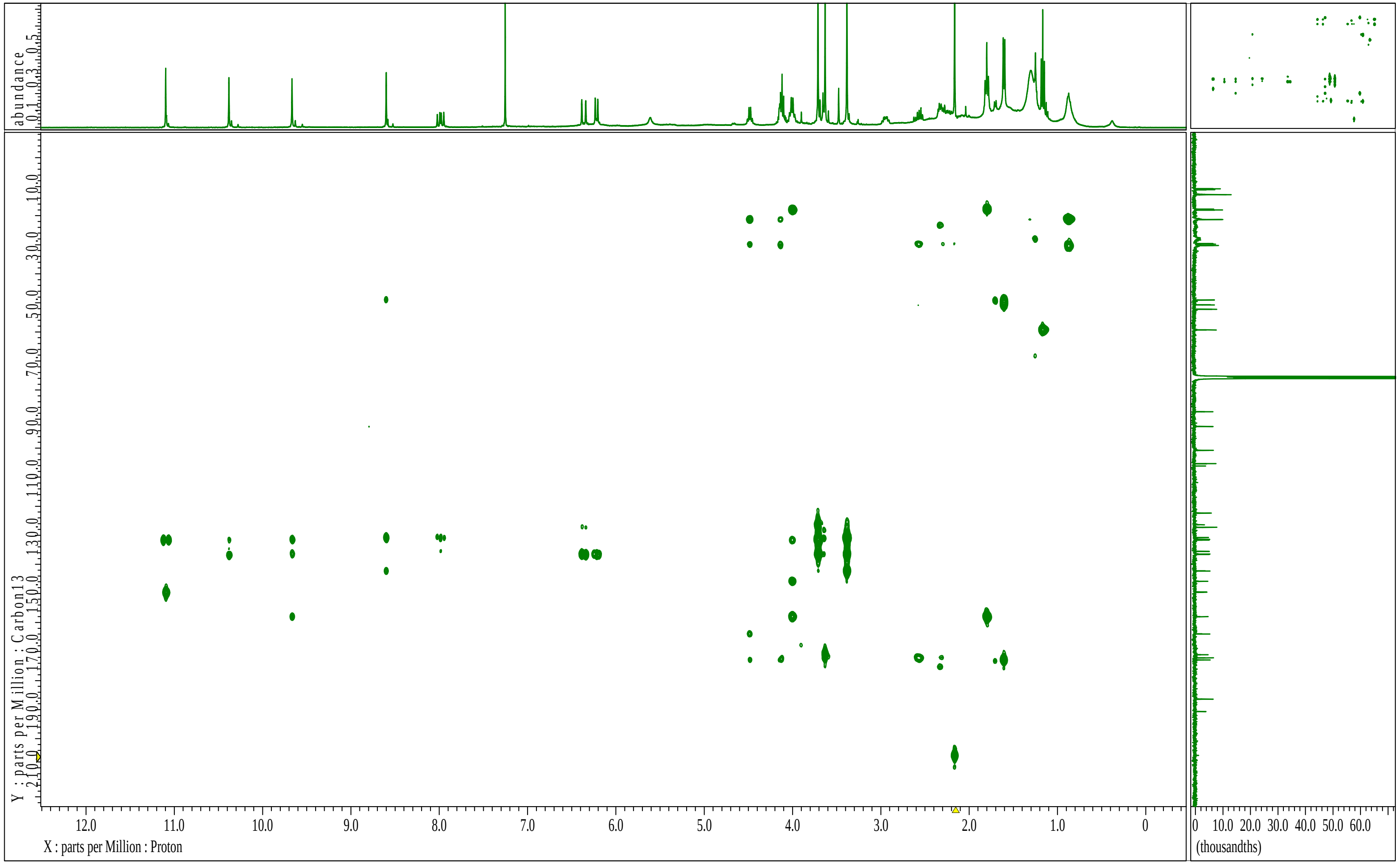

Figure S29. HMBC spectrum of **4** (Recorded in $CDCl_3$)

5. Spectroscopic data for compound 5

Elemental composition	Theoretical m/z	Observed m/z	Error (ppm / mDa)
$C_{37}H_{40}N_4O_7$	653.2970	653.2979	- 1.41 / - 0.92

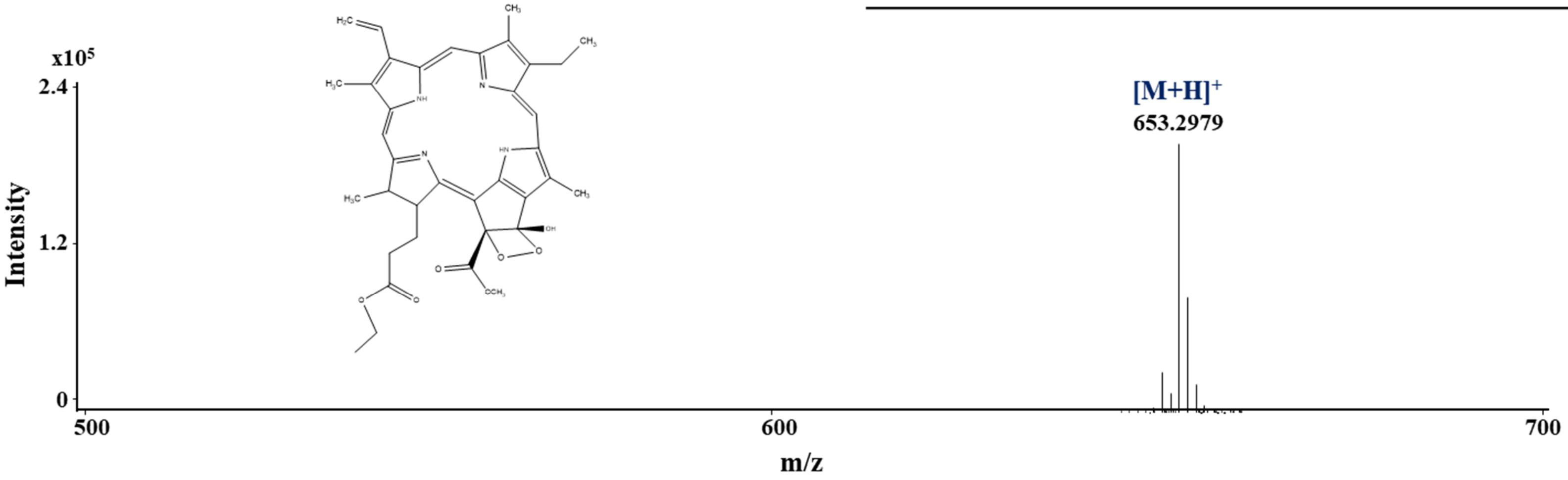

Figure S30. MS spectrum of **5**

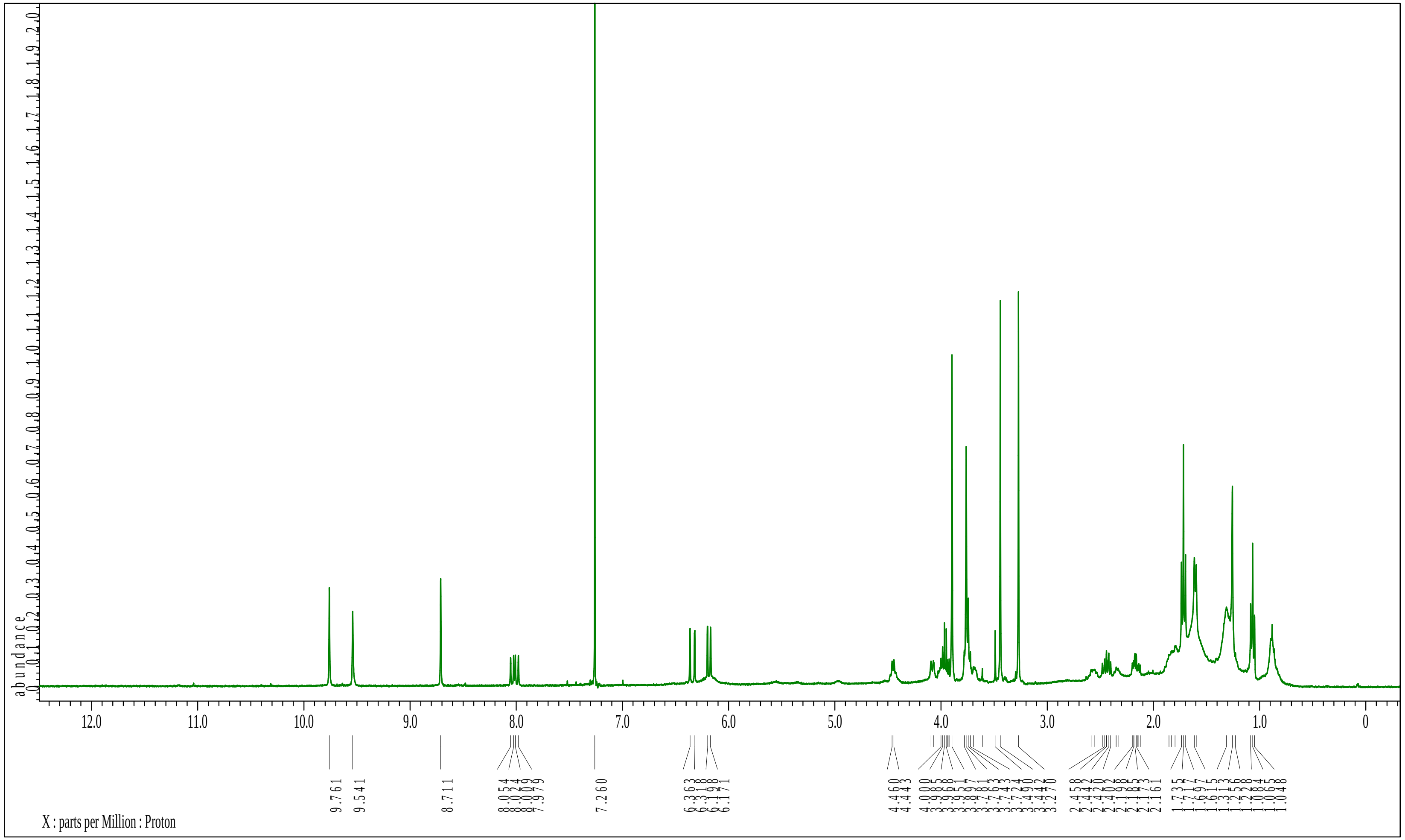

Figure S31. ^{1}H NMR spectrum of **5** (Recorded in $CDCl_3$)

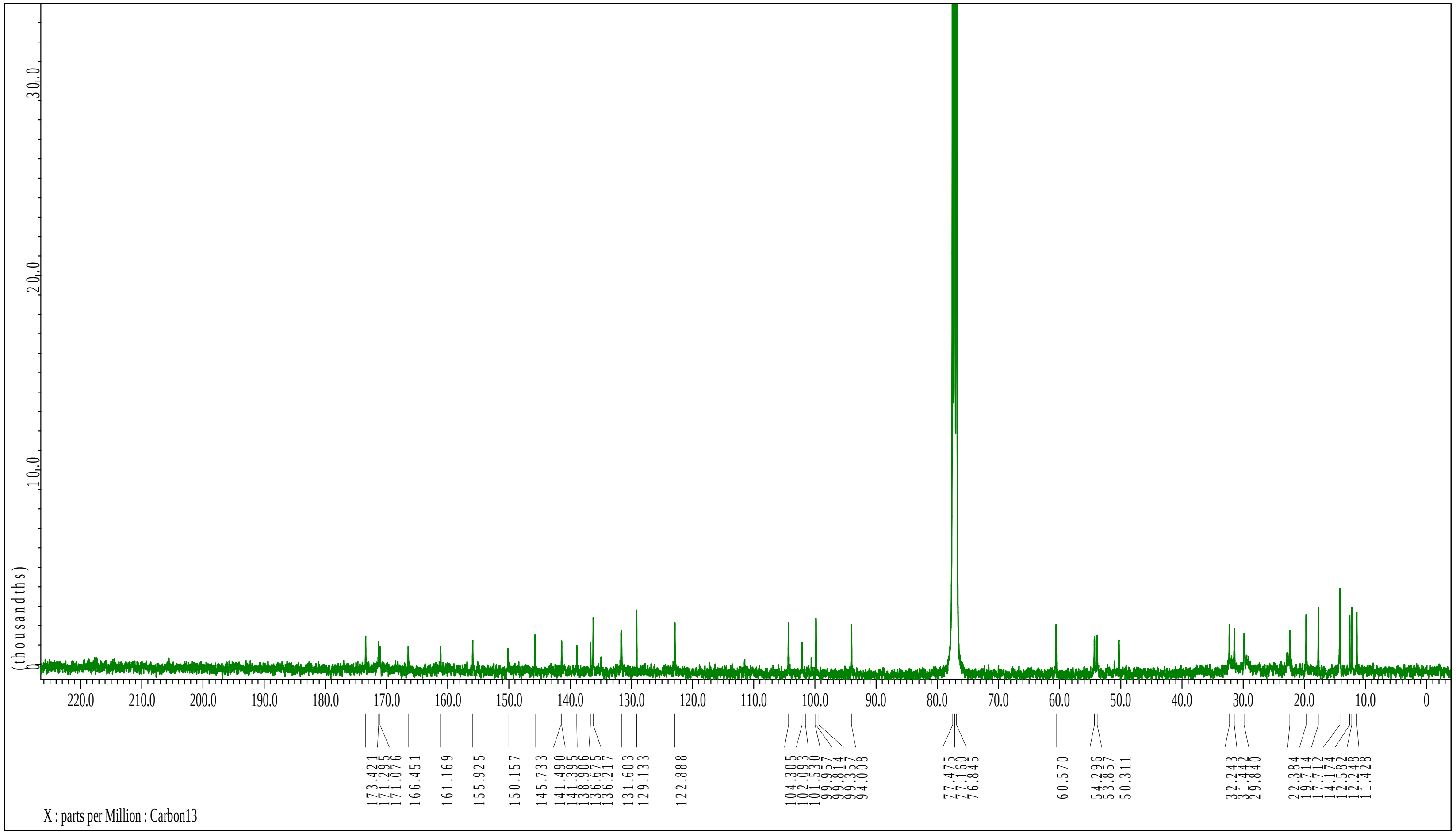

Figure S32. ^{13}C NMR spectrum of **5** (Recorded in $CDCl_3$)

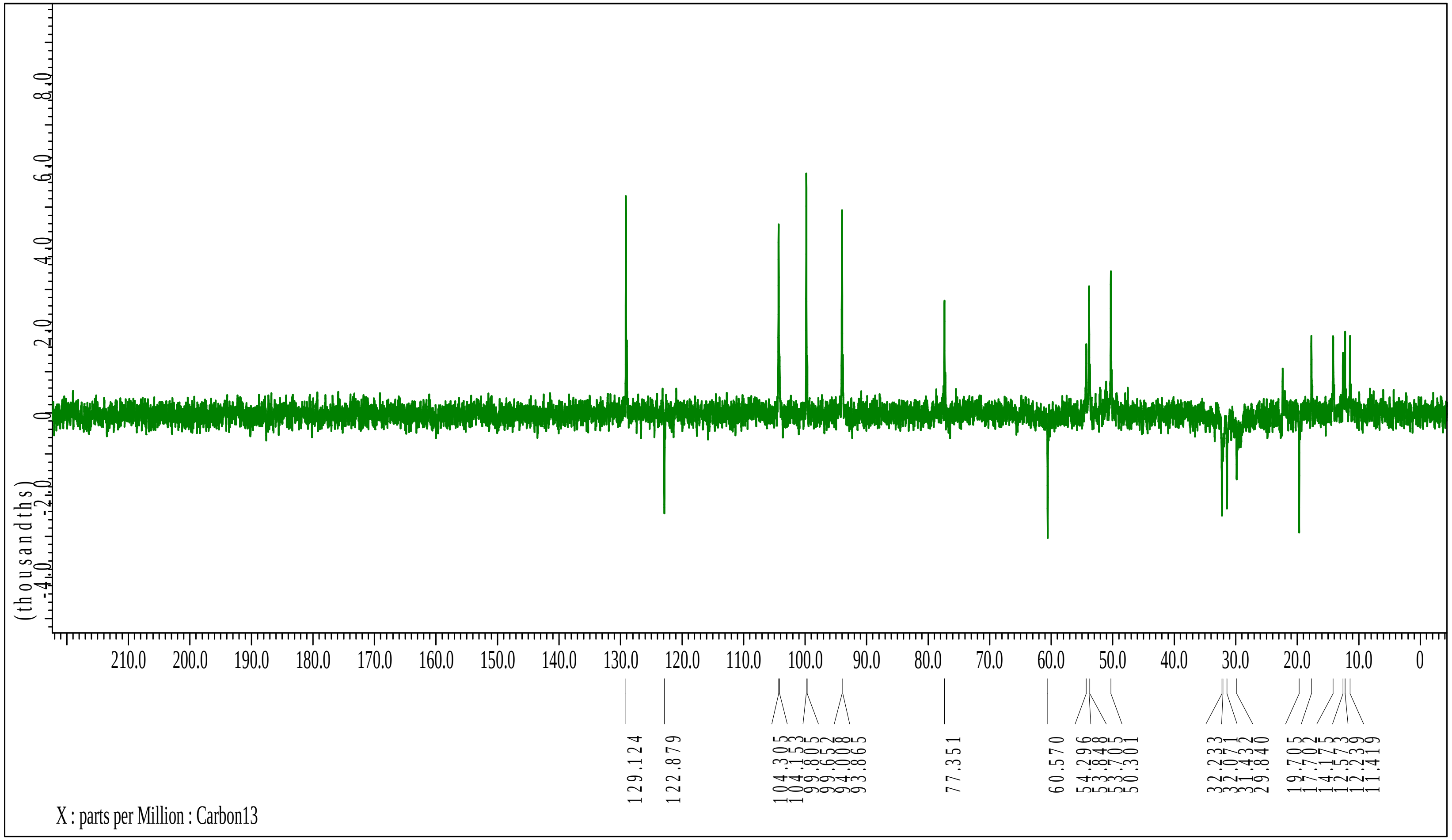

Figure S33. DEPT spectrum of **5** (Recorded in $CDCl_3$)

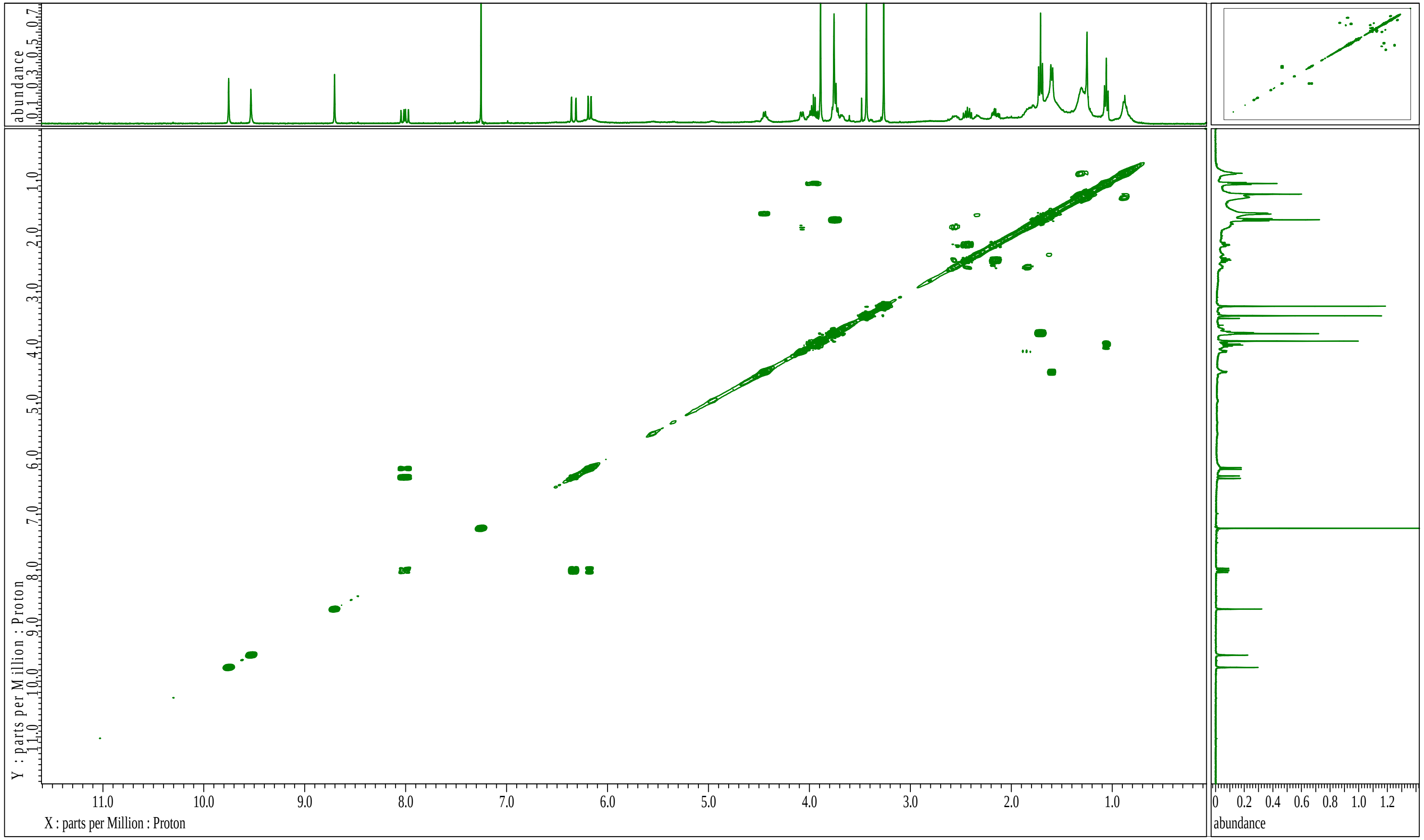

Figure S34. COSY spectrum of **5** (Recorded in $CDCl_3$)

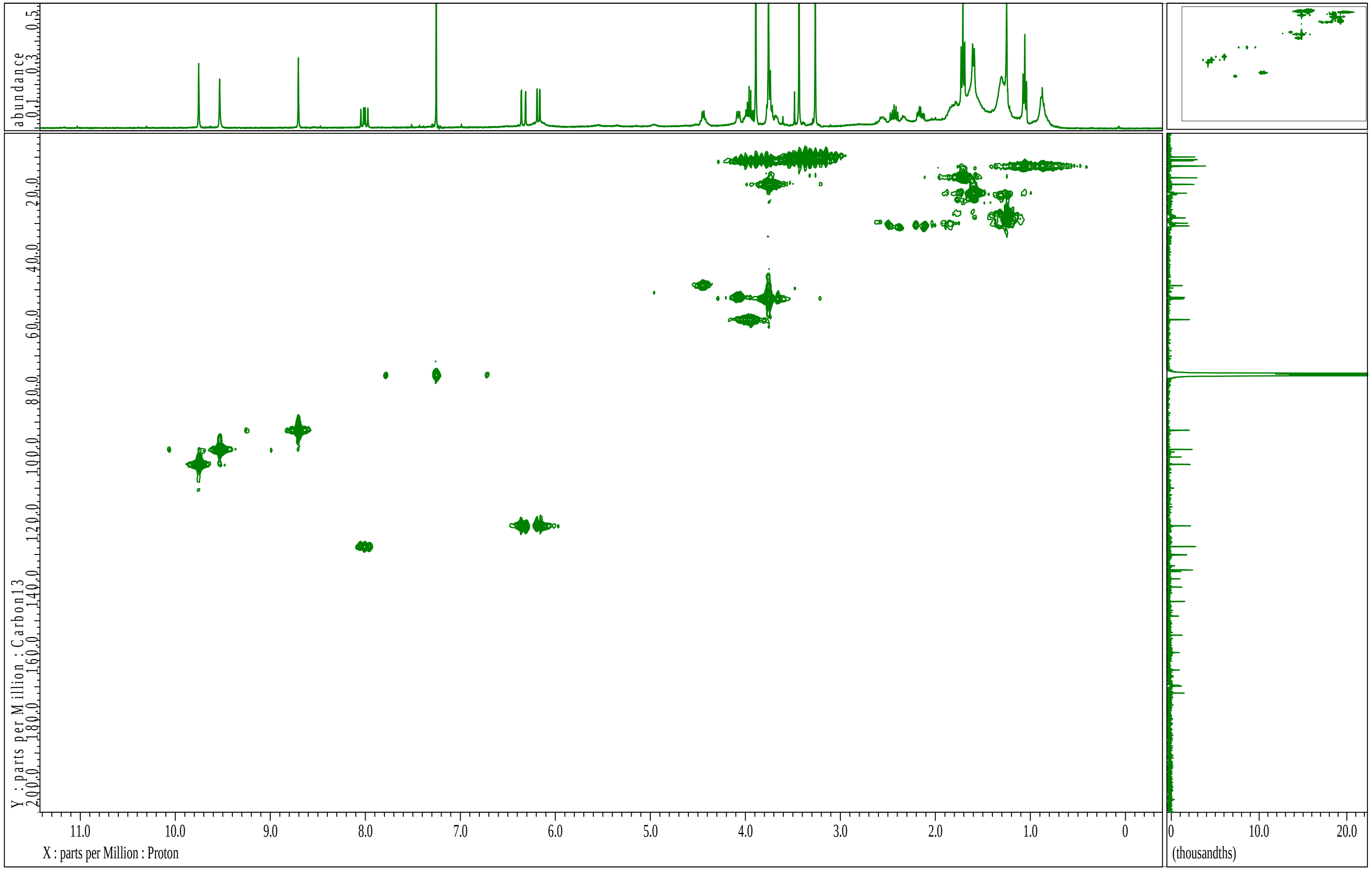

Figure S35. HMQC spectrum of **5** (Recorded in $CDCl_3$)

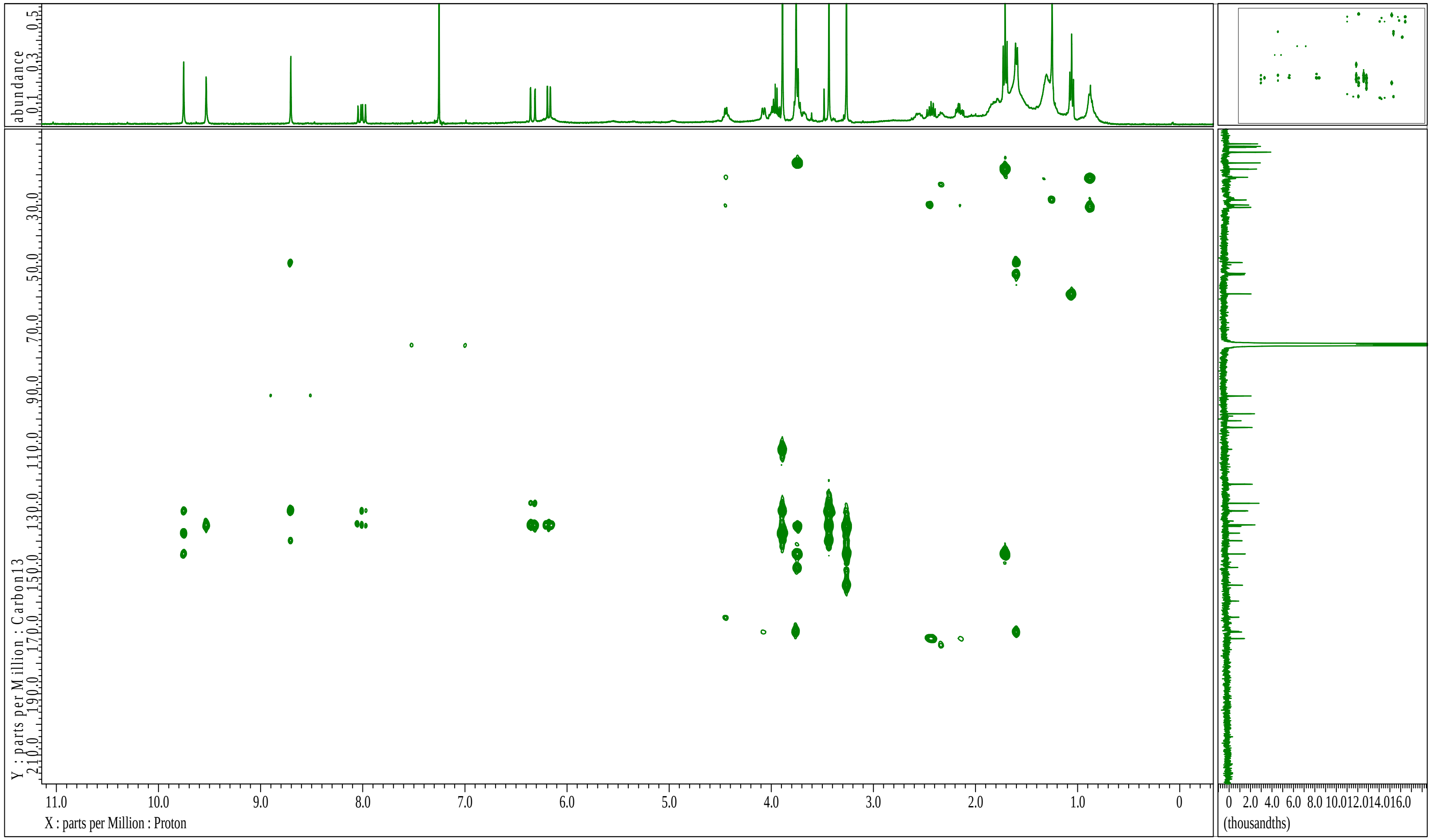

Figure S36. HMBC spectrum of **5** (Recorded in $CDCl_3$)

6. Spectroscopic data for compound 6

Elemental composition	Theoretical m/z	Observed m/z	Error (ppm / mDa)
$C_{22}H_{30}O_4$	358.2144	358.2155	- 3.04 / - 1.09
	359.2217	359.2228	- 3.1 / - 1.11

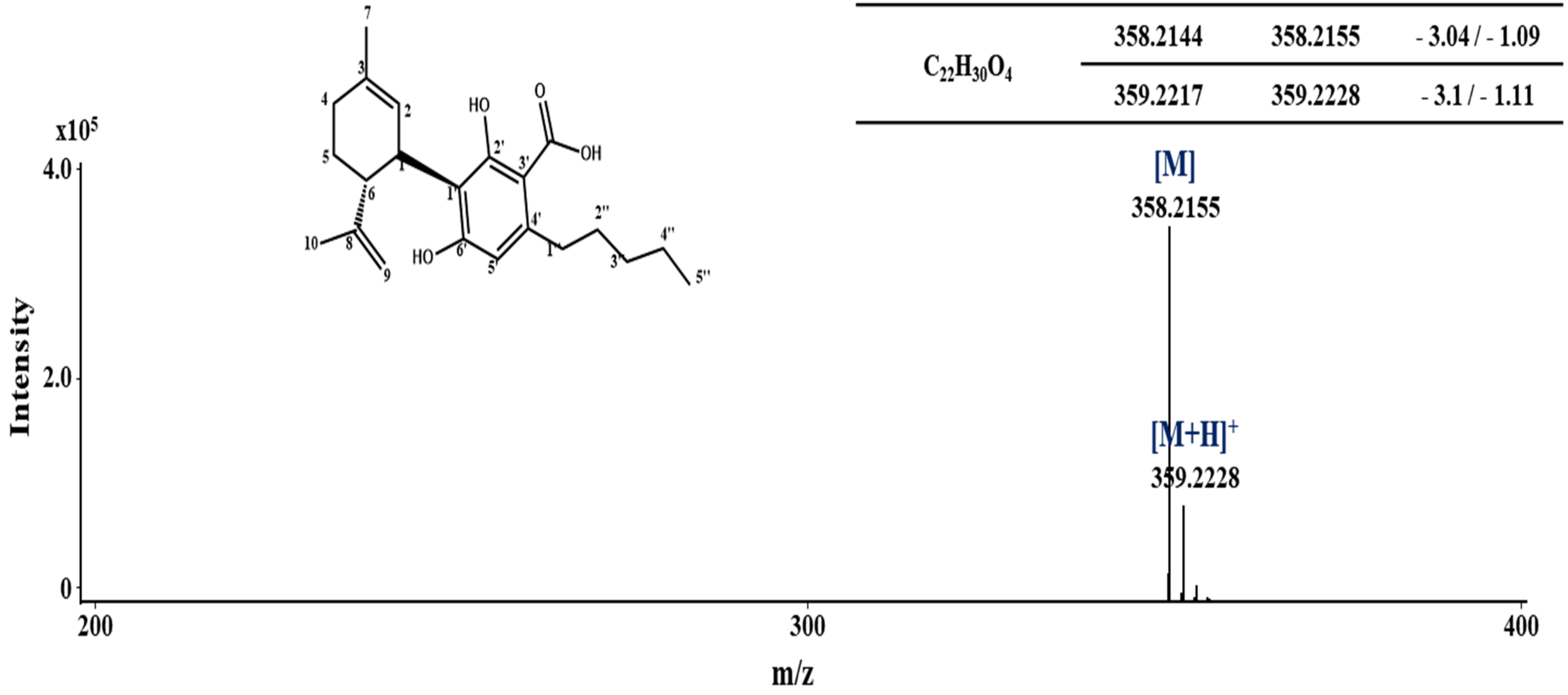

Figure S37. MS spectrum of **6**

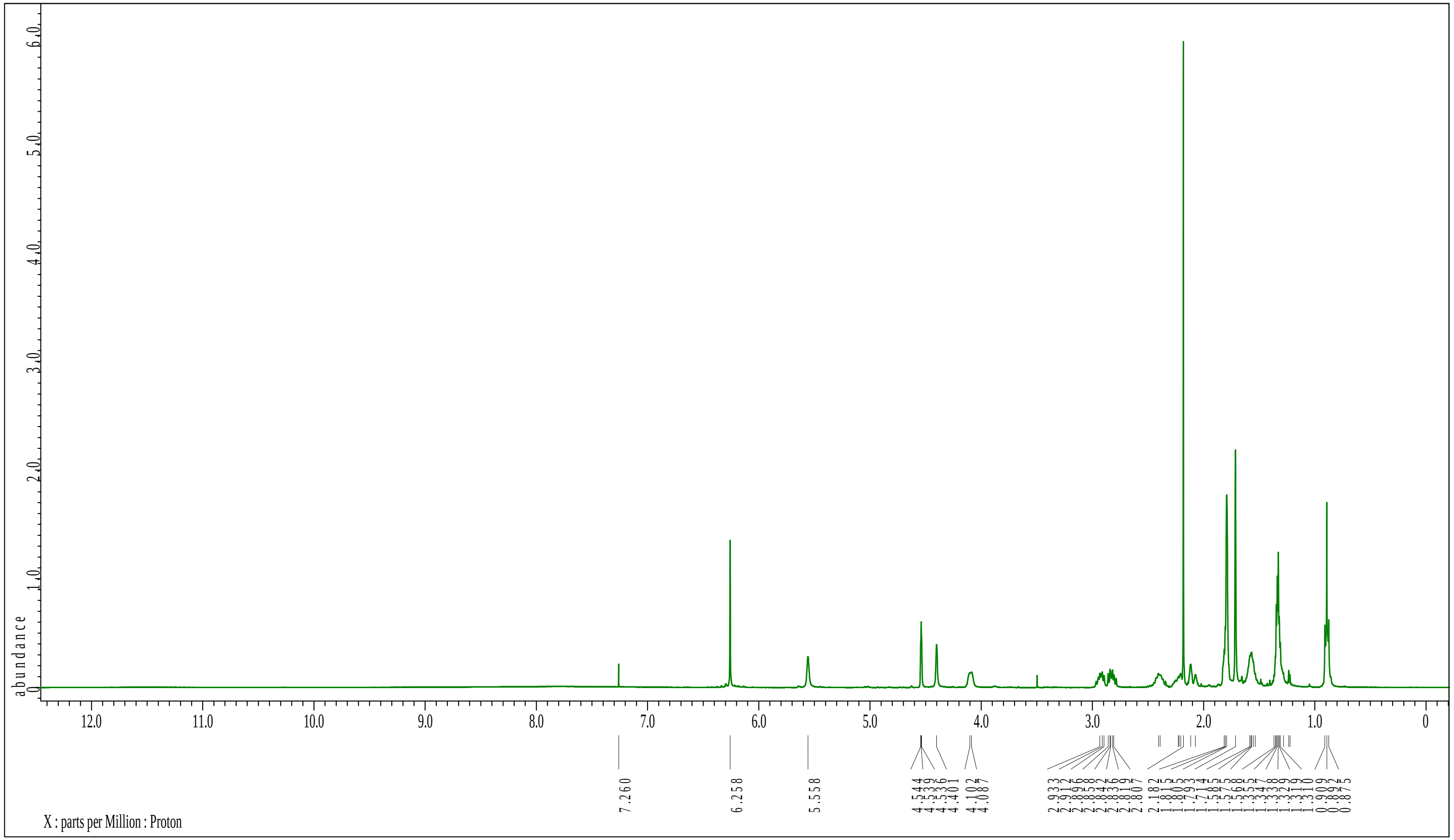

Figure S38. ^{1}H NMR spectrum of **6** (Recorded in $CDCl_3$)

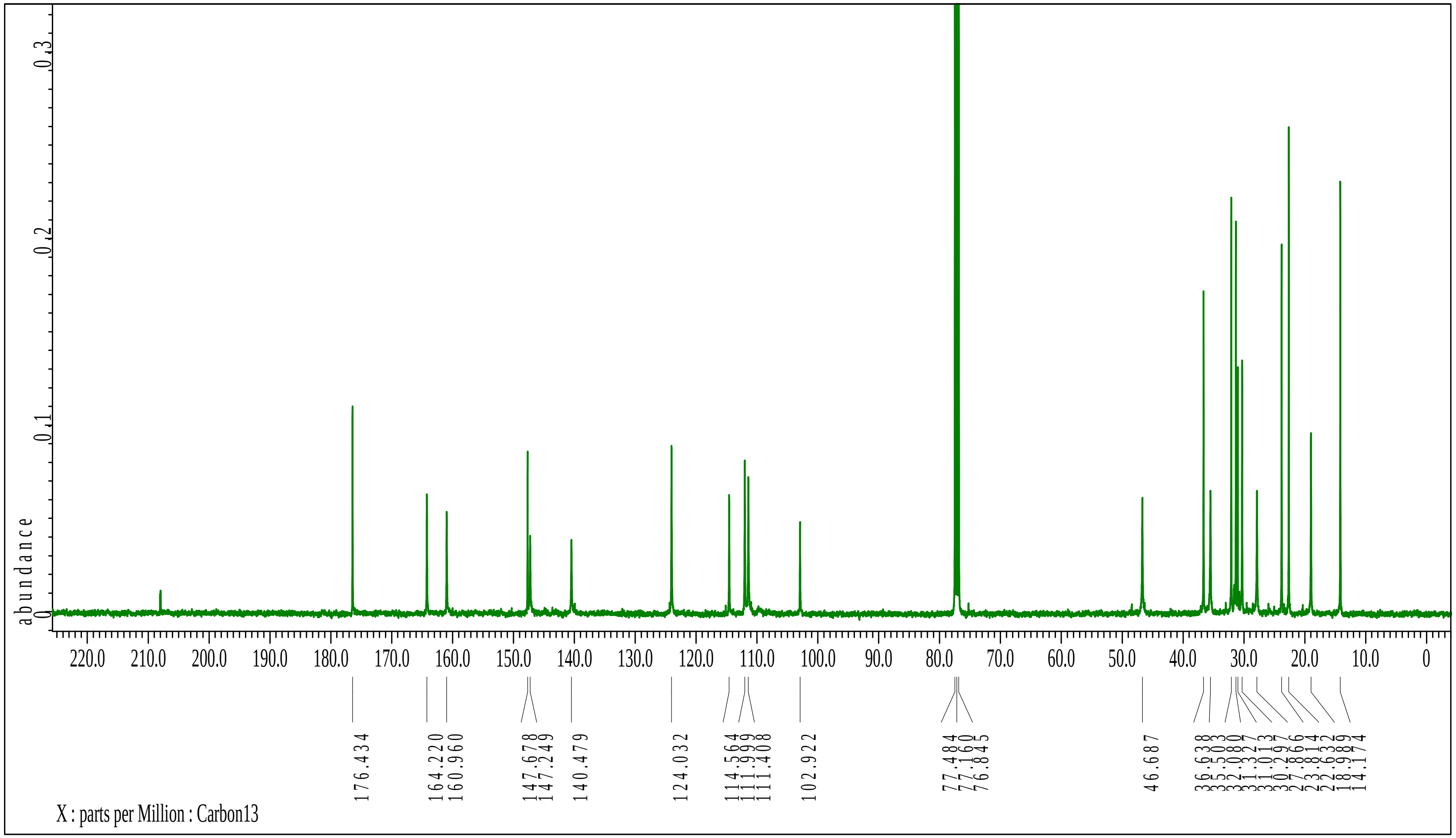

Figure S39. ^{13}C NMR spectrum of **6** (Recorded in $CDCl_3$)

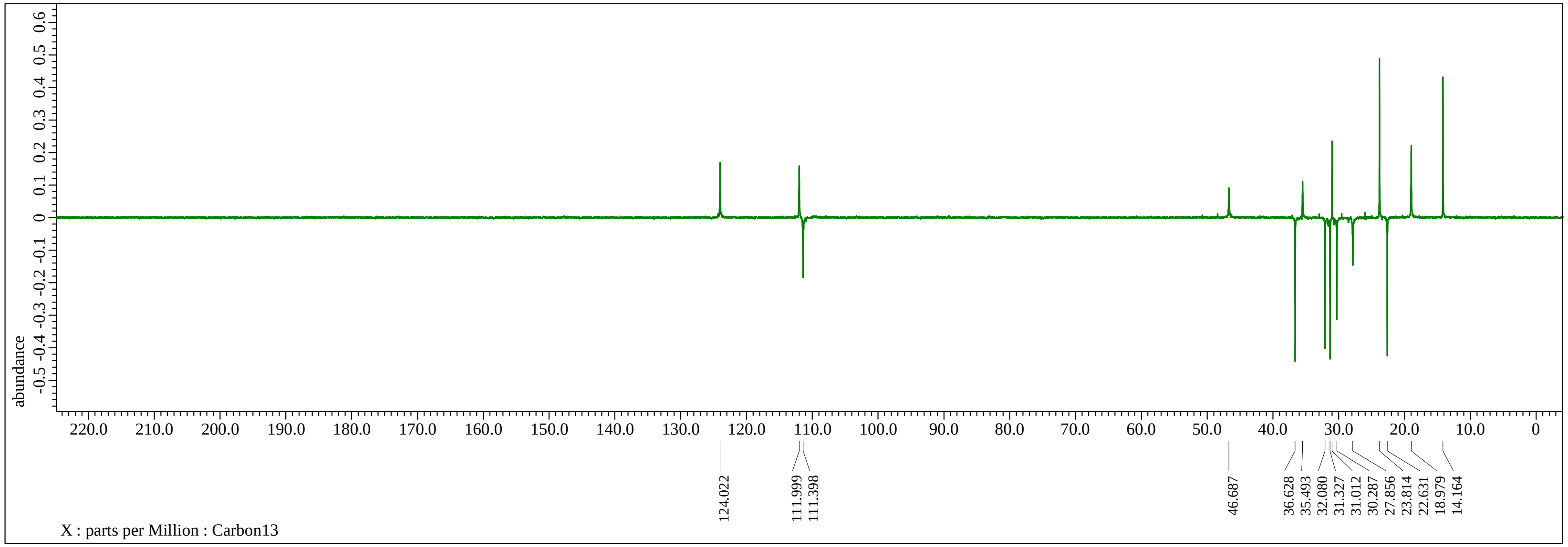

Figure S40. DEPT spectrum of **6** (Recorded in $CDCl_3$)

7. Spectroscopic data for compound 7

Elemental composition	Theoretical m/z	Observed m/z	Error (ppm / mDa)
$C_{23}H_{32}O_4$	373.2373	373.2368	1.44 / 0.54

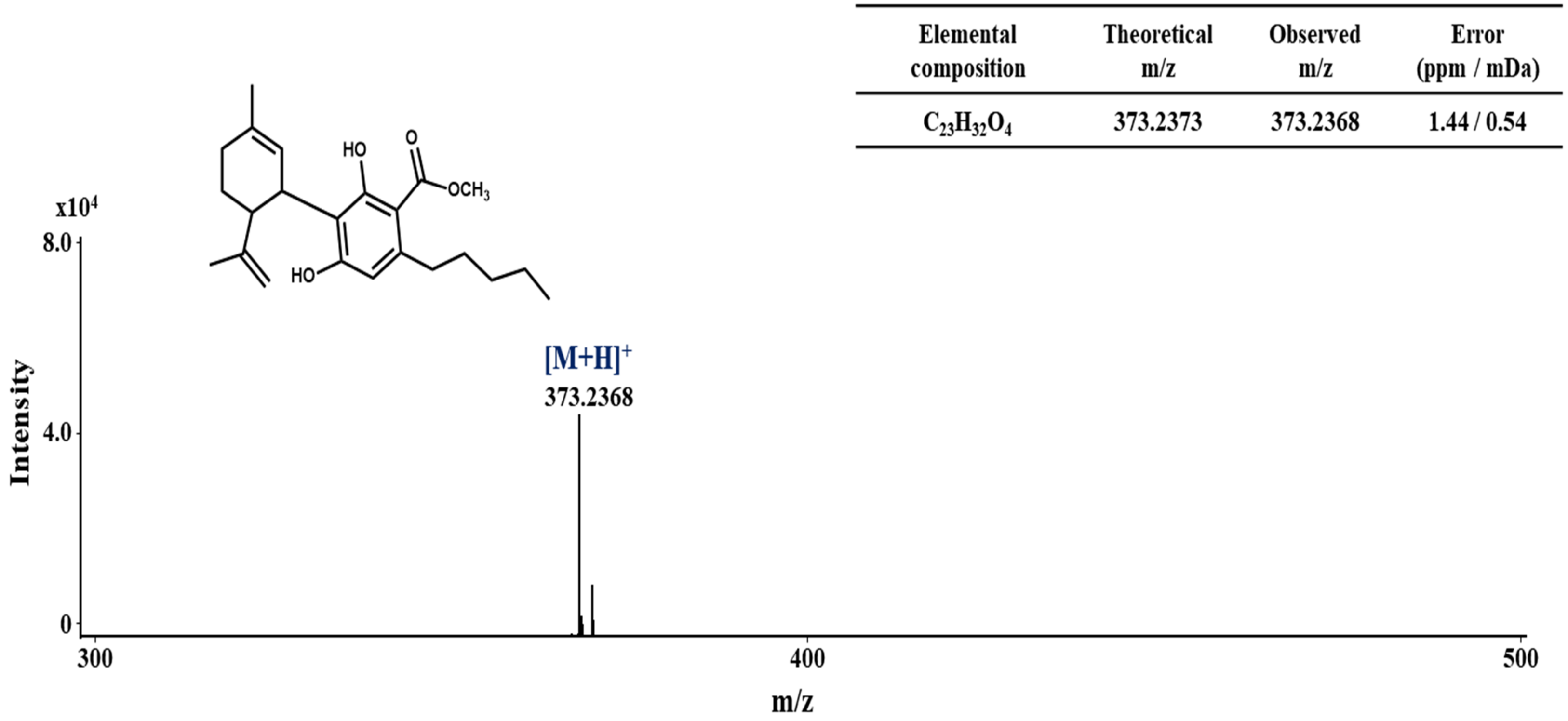

Figure S41. MS spectrum of **7**

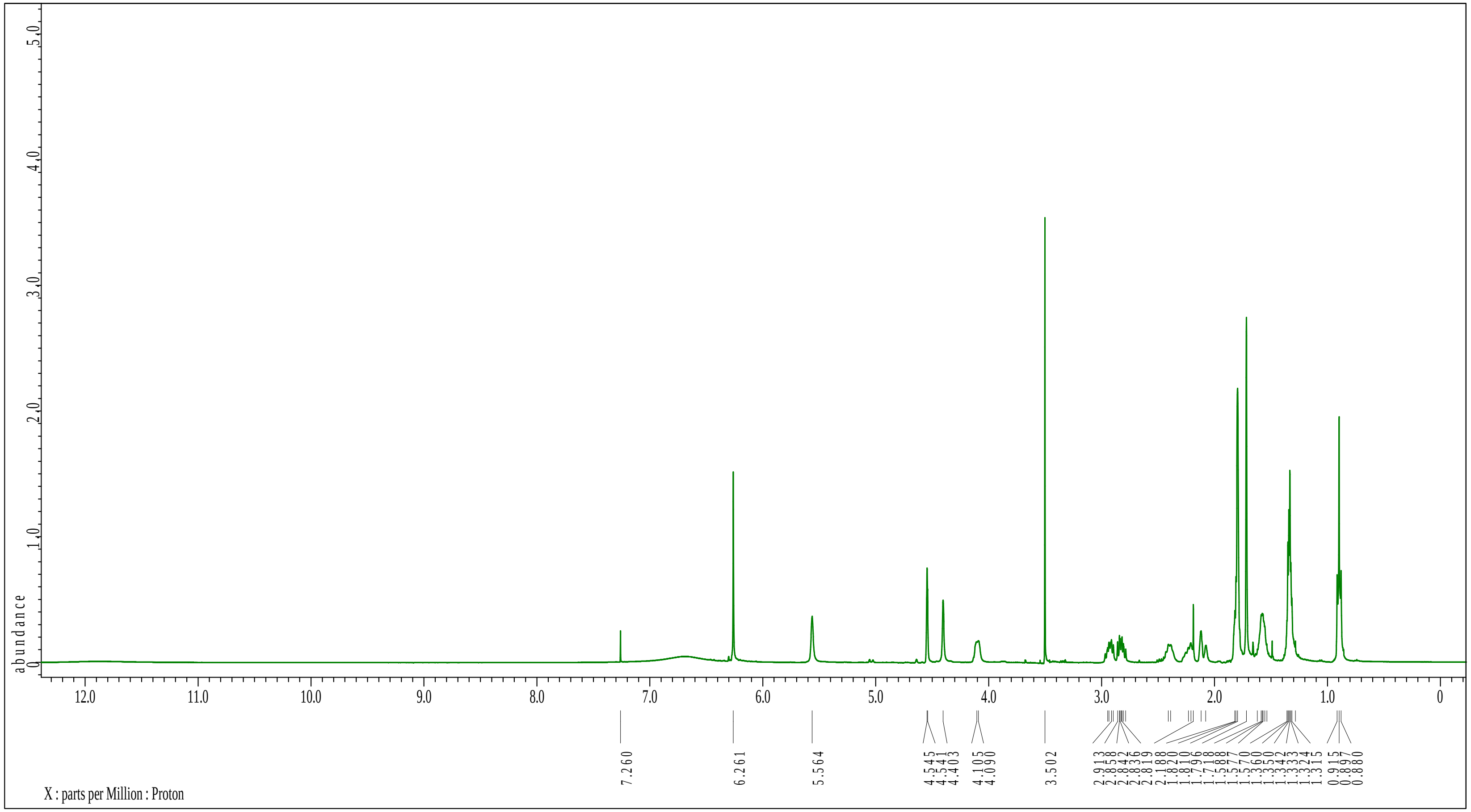

Figure S42. ^{1}H NMR spectrum of **7** (Recorded in $CDCl_3$)

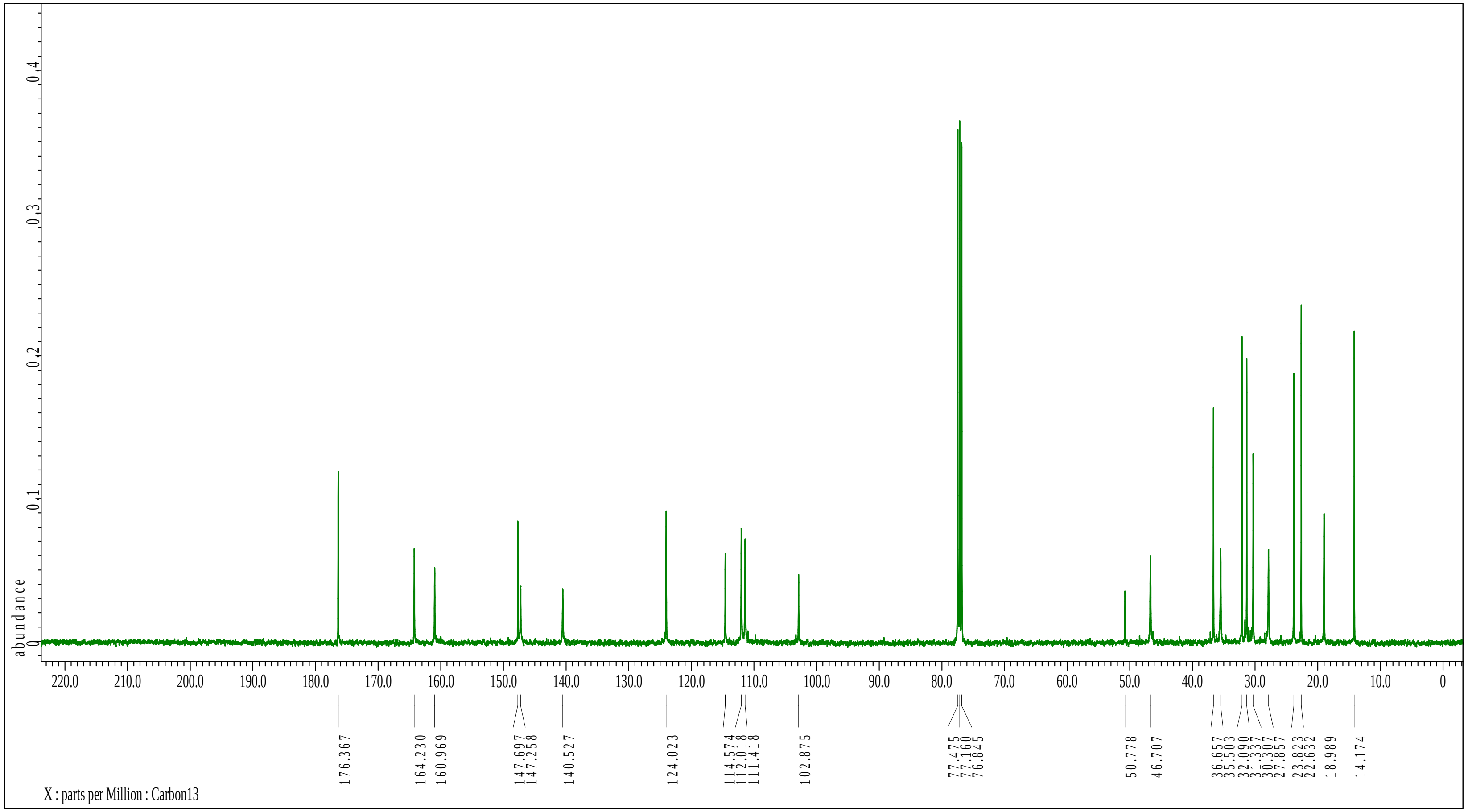

Figure S43. ^{13}C NMR spectrum of **7** (Recorded in $CDCl_3$)

8. Spectroscopic data for compound 8

Elemental composition	Theoretical m/z	Observed m/z	Error (ppm / mDa)
$C_{21}H_{30}O_2$	315.2319	315.2326	-2.36 / -0.74

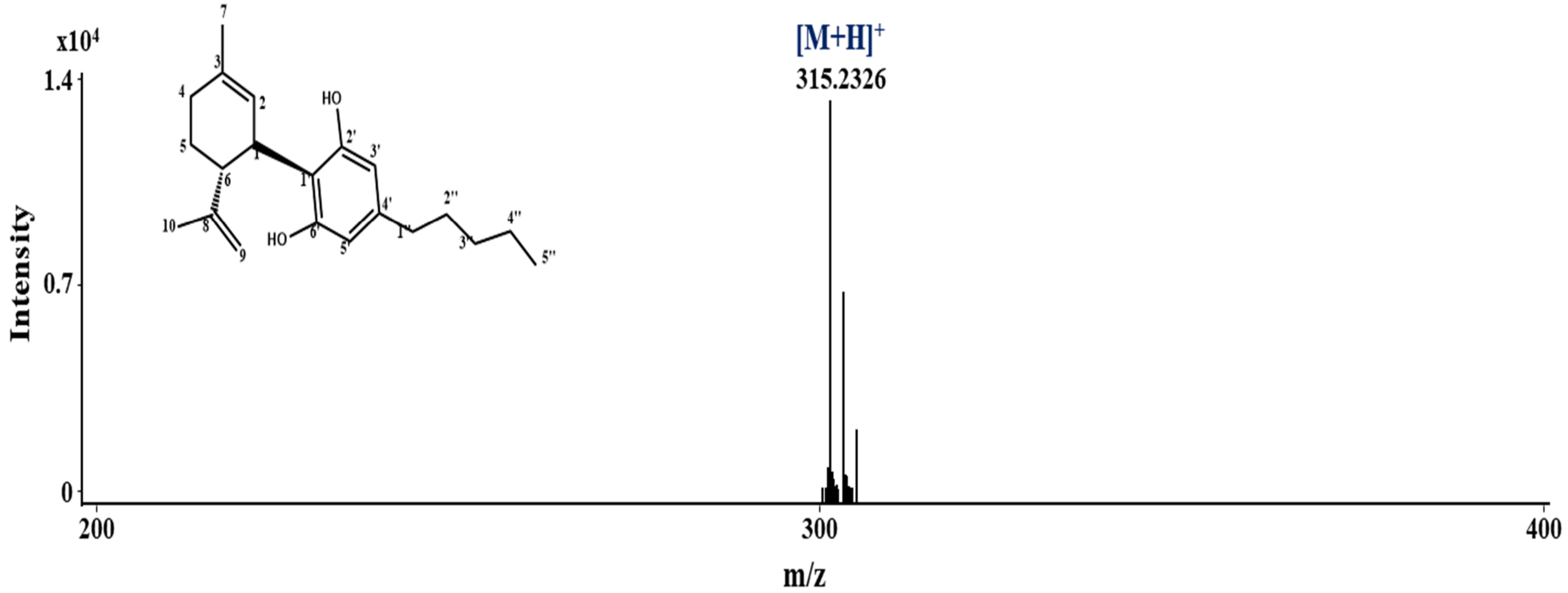

Figure S44. MS spectrum of **8**

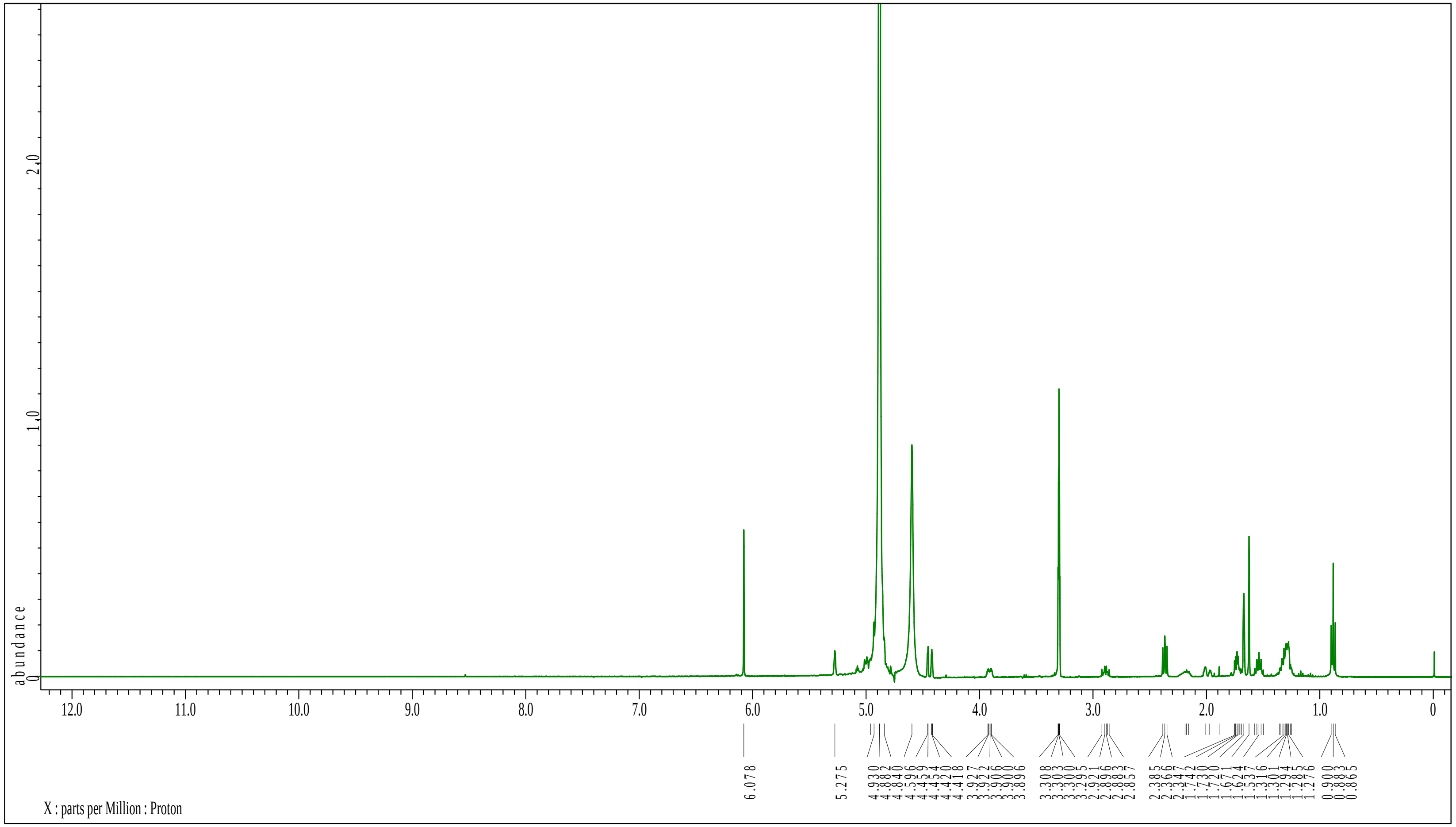

Figure S45. ^{1}H NMR spectrum of **8** (Recorded in CD_3OD)

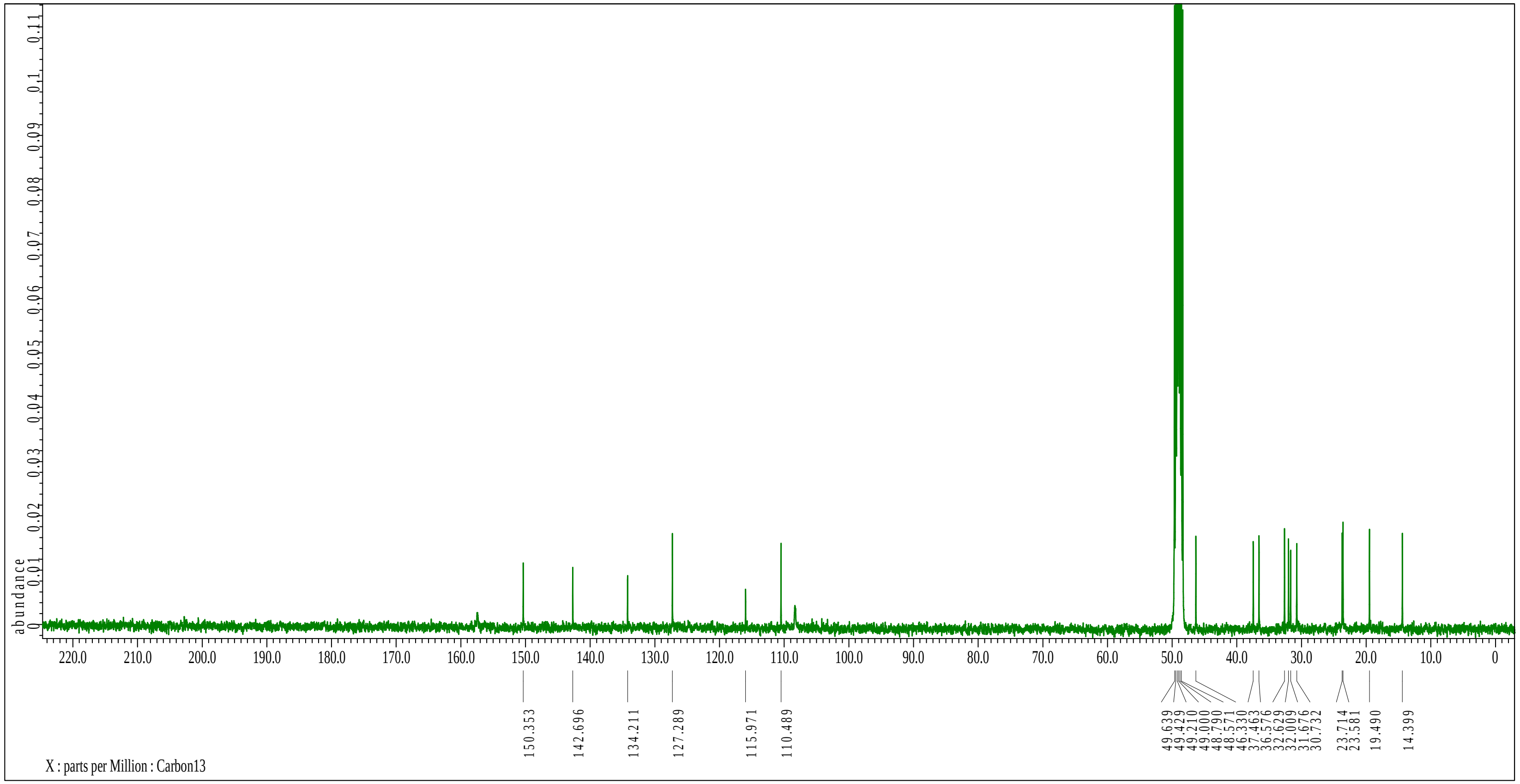

Figure S46. ^{13}C NMR spectrum of **8** (Recorded in CD_3OD)

9. Spectroscopic data for compound 9

Elemental composition	Theoretical m/z	Observed m/z	Error (ppm / mDa)
$C_{21}H_{30}O_2$	315.2319	315.2233	- 4.58 / - 1.44

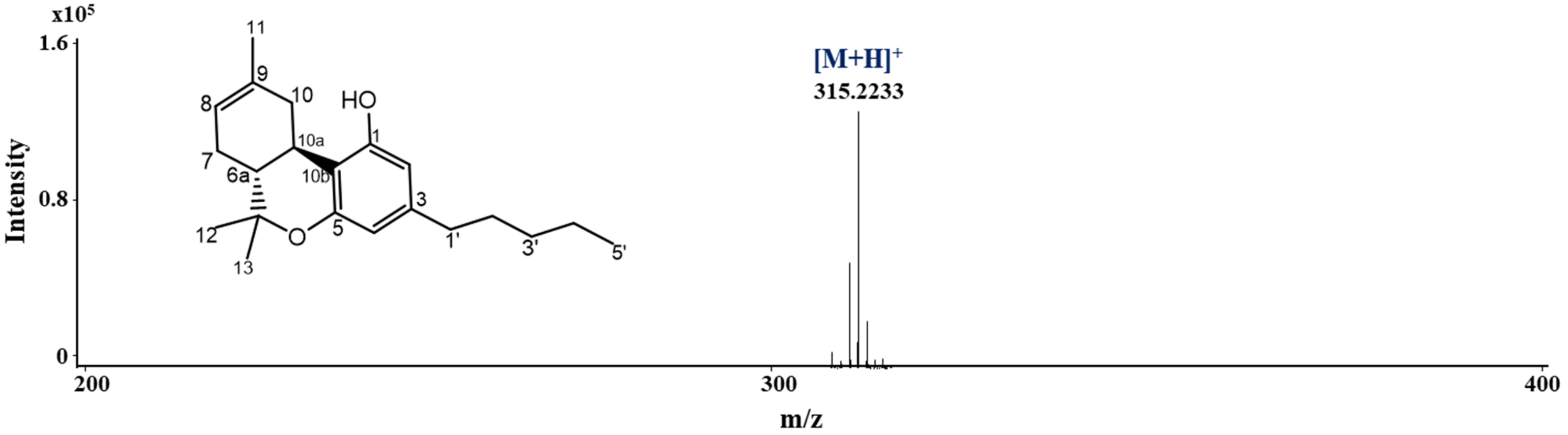

Figure S47. MS spectrum of **9**

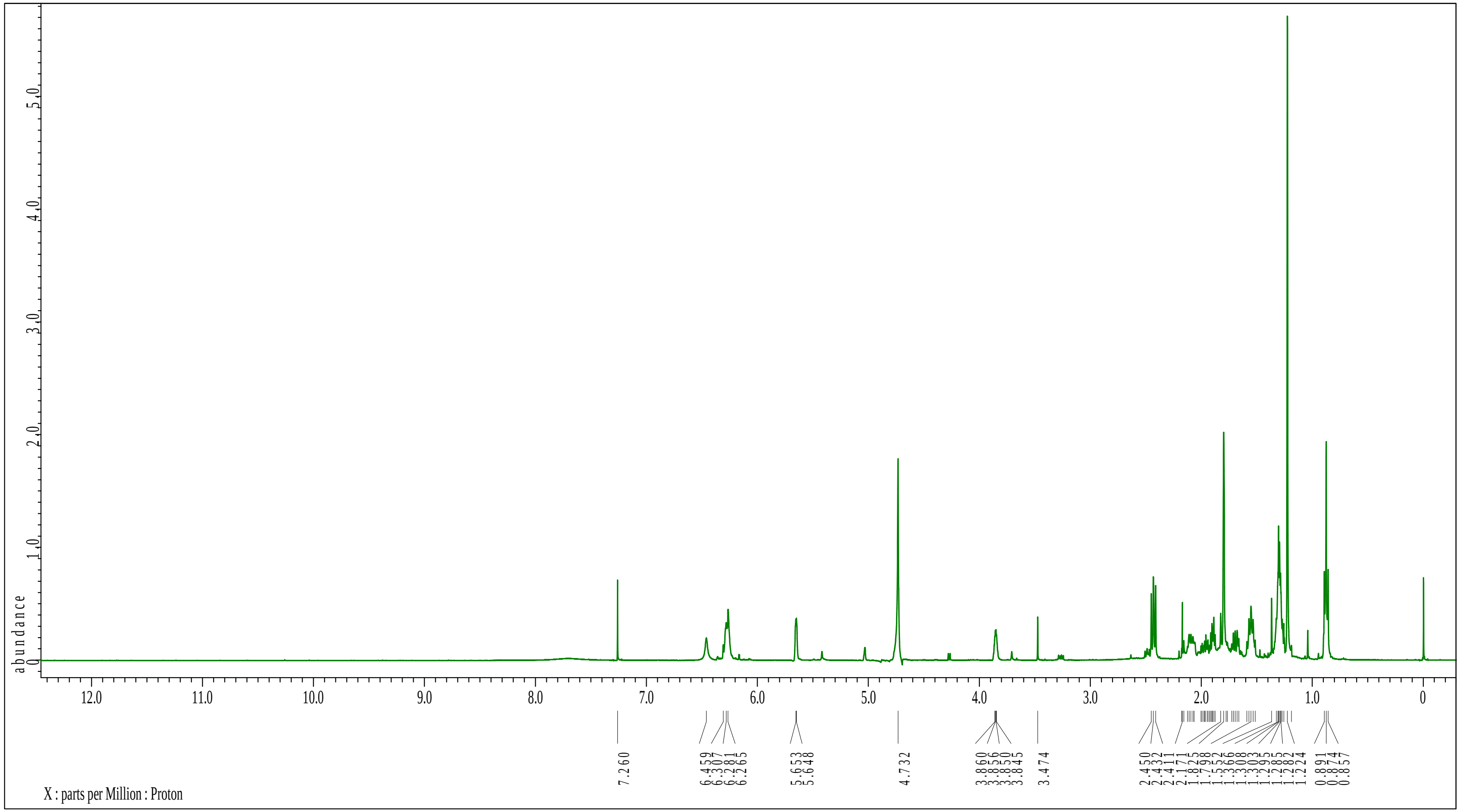

Figure S48. ^{1}H NMR spectrum of **9** (Recorded in $CDCl_3$)

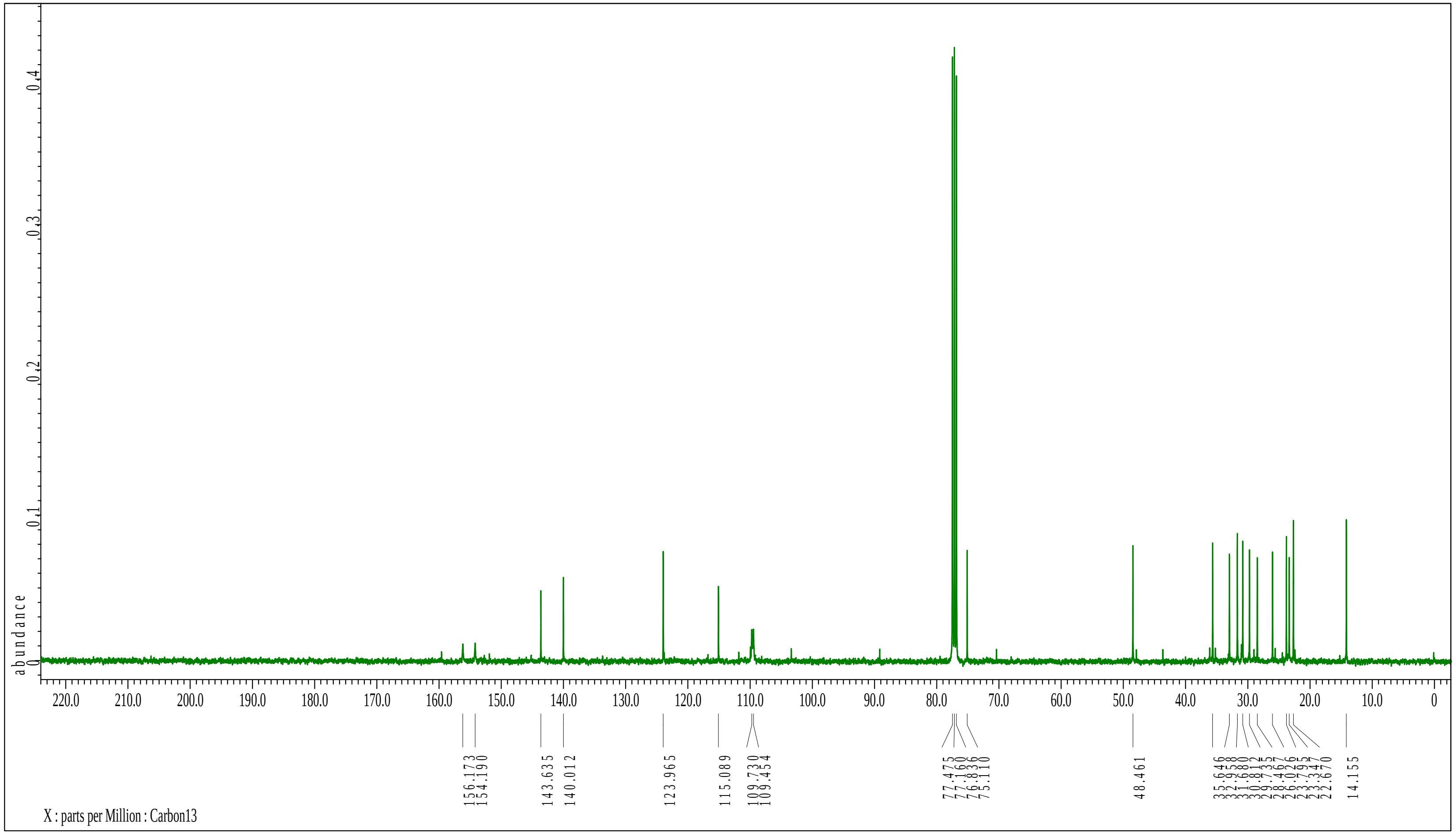

Figure S49. ^{13}C NMR spectrum of **9** (Recorded in $CDCl_3$)

10. Spectroscopic data for compound 10

Elemental composition	Theoretical m/z	Observed m/z	Error (ppm / mDa)
$C_{21}H_{30}O_2$	315.2319	315.2305	- 0.14 / - 0.04

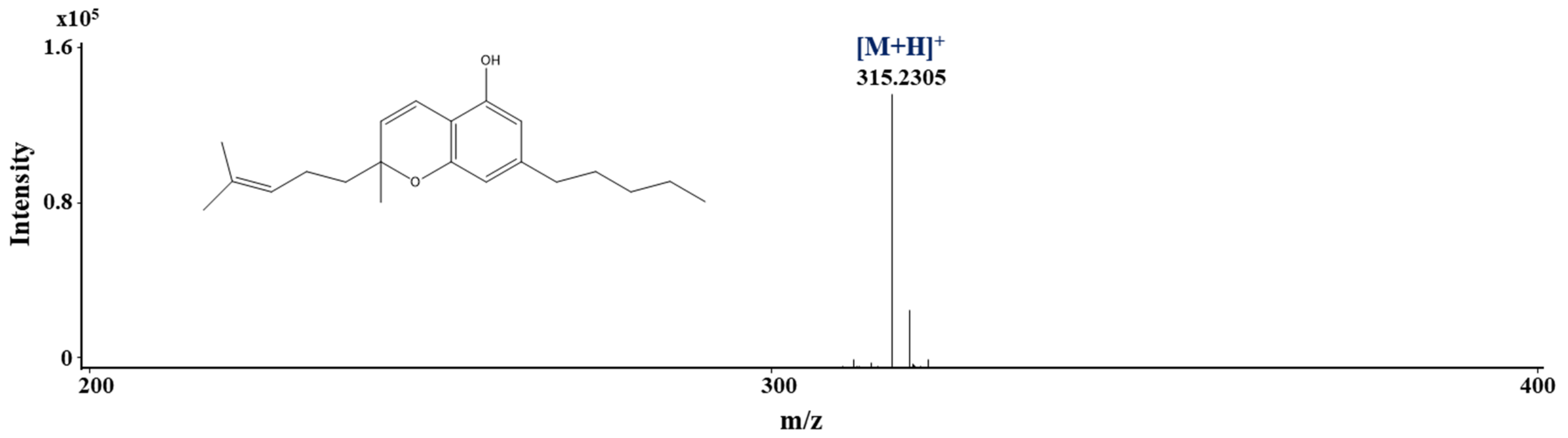

Figure S50. MS spectrum of **10**

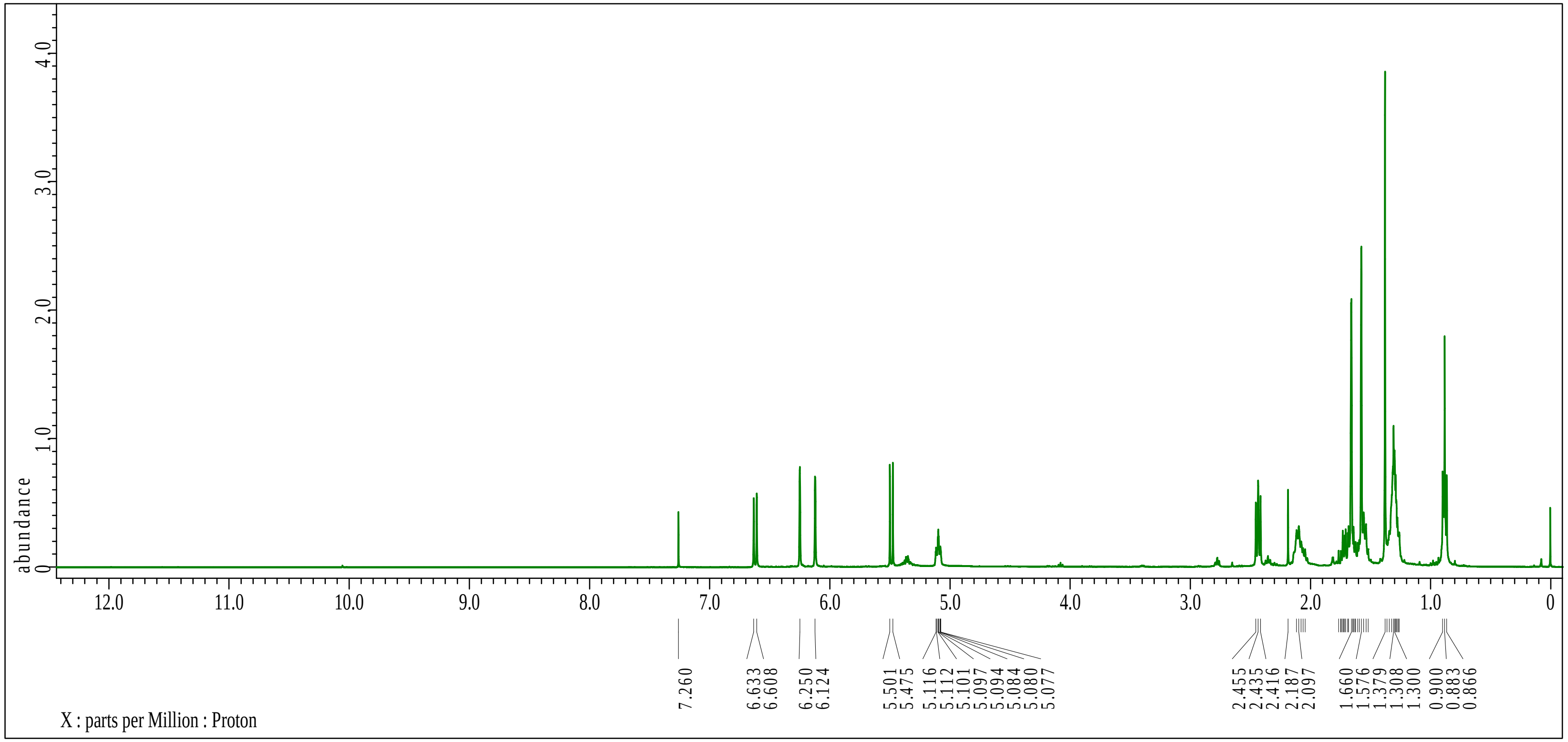

Figure S51. ^{1}H NMR spectrum of **10** (Recorded in $CDCl_3$)

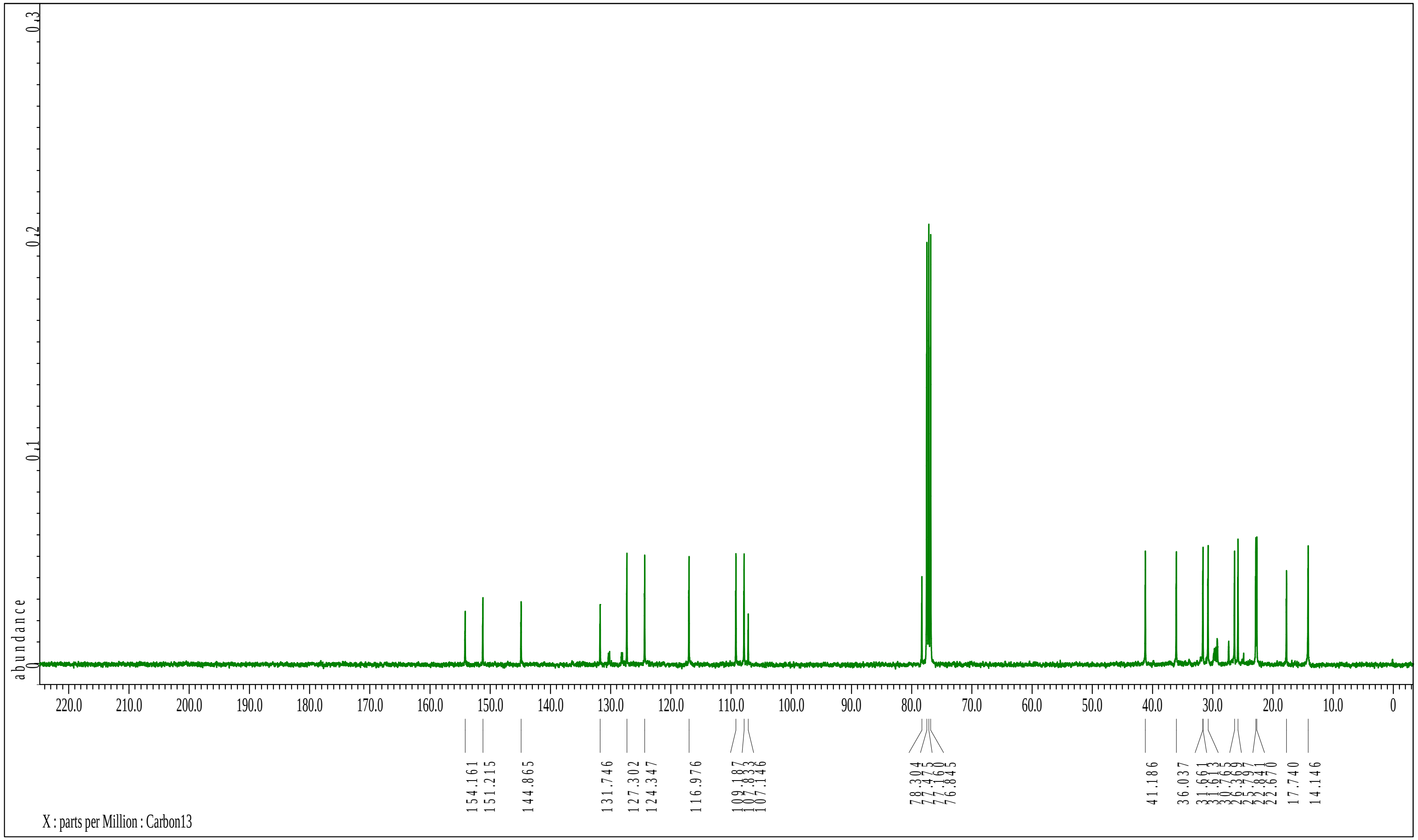

Figure S52. ^{13}C NMR spectrum of **10** (Recorded in $CDCl_3$)

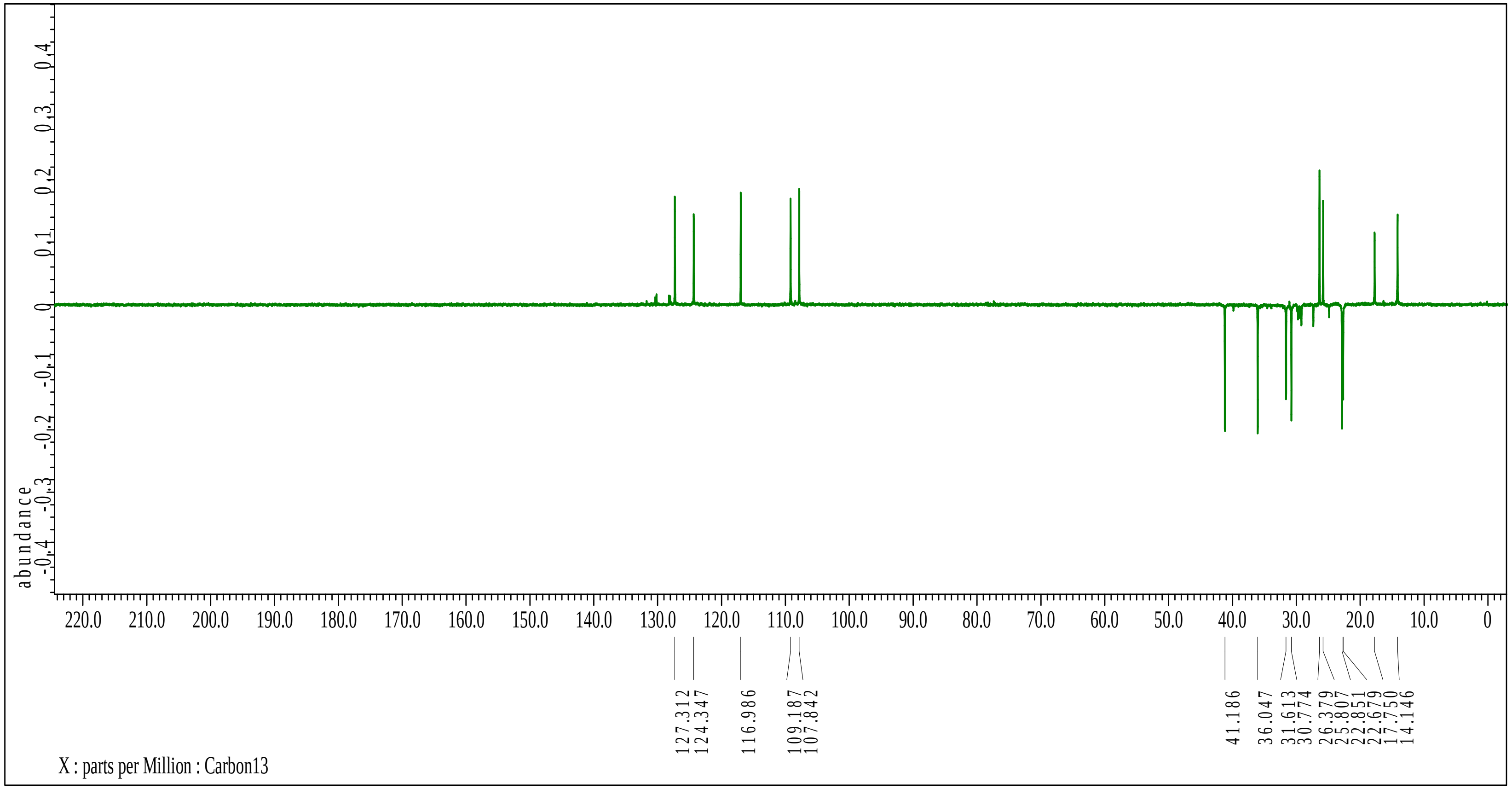

Figure S53. DEPT spectrum of **10** (Recorded in $CDCl_3$)

11. Spectroscopic data for compound 11

Elemental composition	Theoretical m/z	Observed m/z	Error (ppm / mDa)
$C_{21}H_{32}O_4$	349.2373	349.2370	0.96 / 0.34

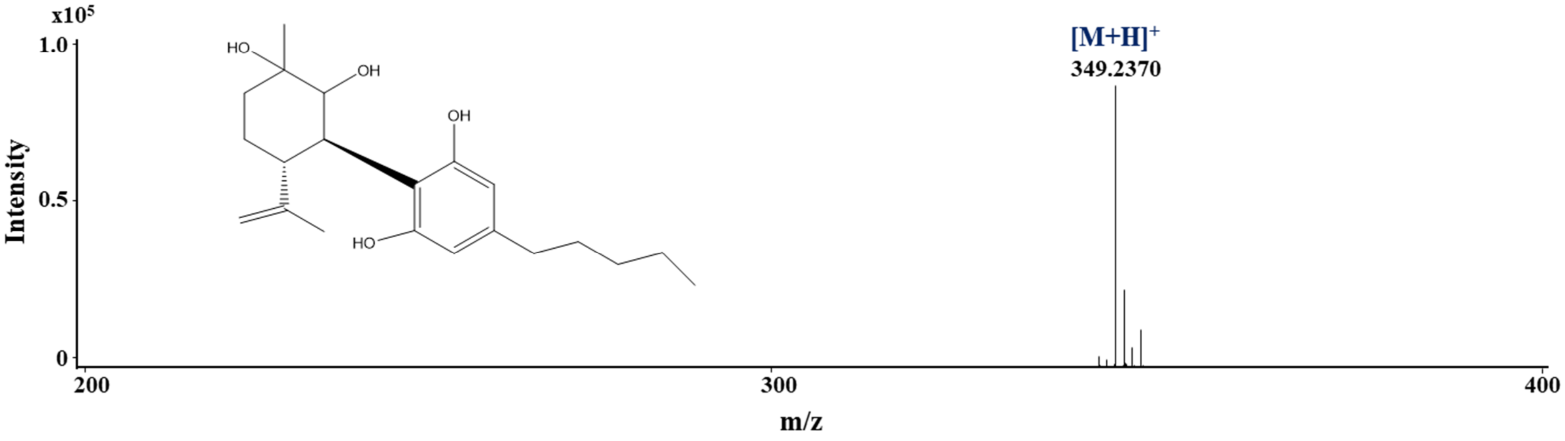

Figure S54. MS spectrum of **11**

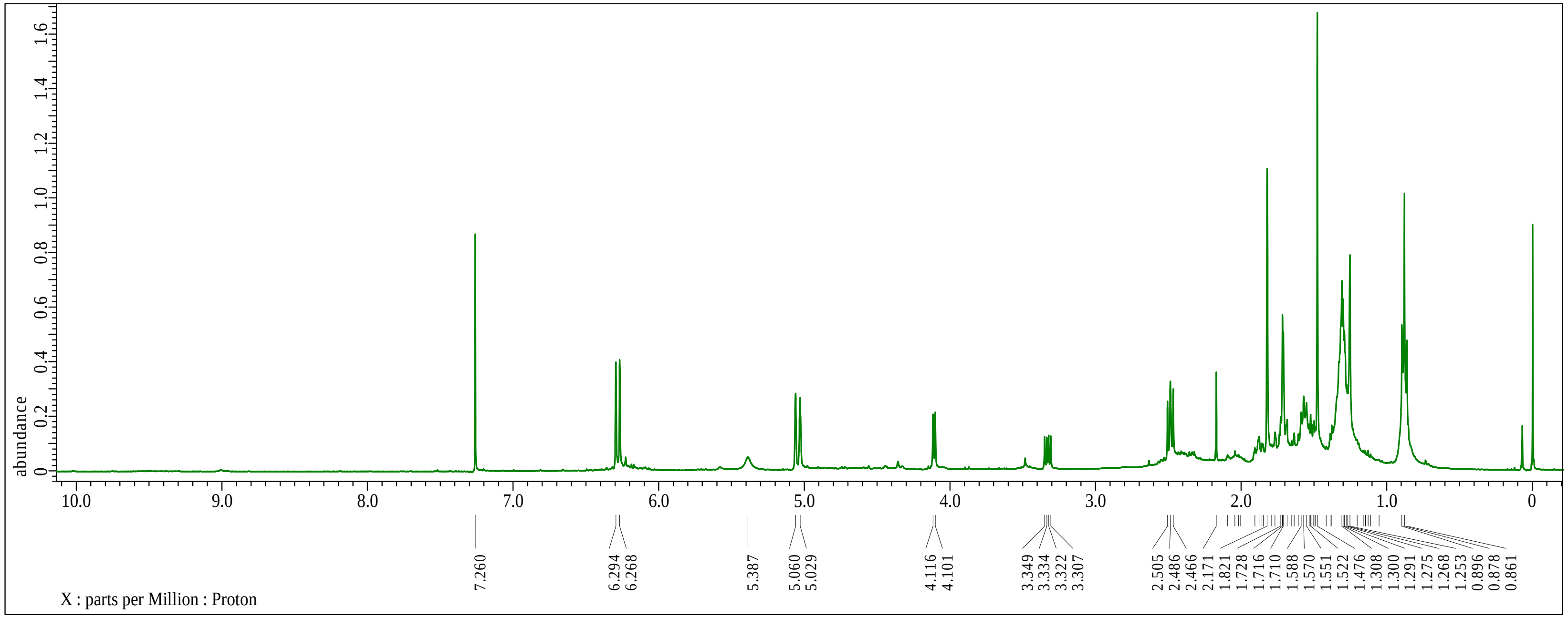

Figure S55. ^{1}H NMR spectrum of **11** (Recorded in $CDCl_3$)

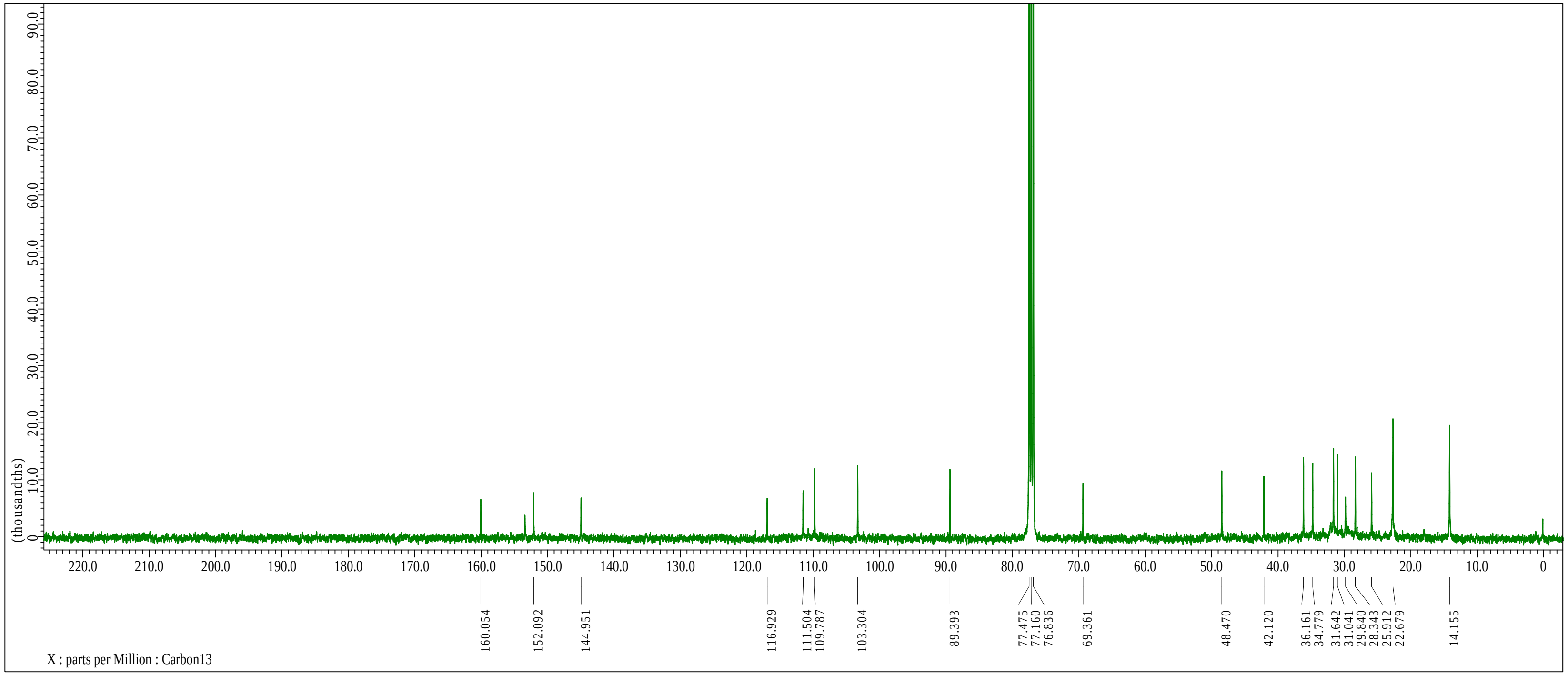

Figure S56. ^{13}C NMR spectrum of **11** (Recorded in $CDCl_3$)

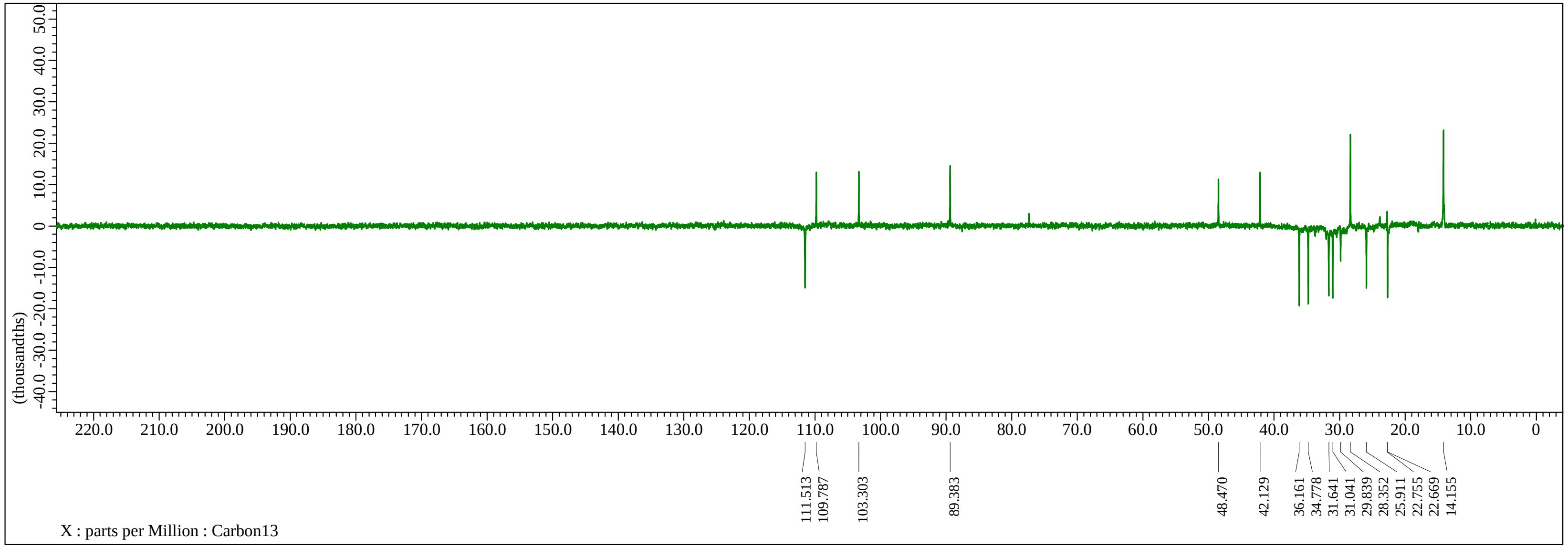

Figure S57. DEPT spectrum of **11** (Recorded in $CDCl_3$)
